# Design and Structural Transformations of Zinc(II) Knotted Cage Frameworks

**DOI:** 10.1002/anie.202519491

**Published:** 2025-10-16

**Authors:** Yuchong Yang, Sabrina Y. Hu, Tanya K. Ronson, Paula C.P. Teeuwen, Sudhakar Gaikwad, Andrew W. Heard, David J. Wales, Jonathan R. Nitschke

**Affiliations:** ^1^ Yusuf Hamied Department of Chemistry University of Cambridge Cambridge CB2 1EW United Kingdom

**Keywords:** Host–guest chemistry, Interwoven structures, Metal‐organic cages, Self‐assembly, Subcomponent exchange

## Abstract

Interwoven architectures feature in biomolecules such as proteins, DNA, and RNA. However, the high‐yielding syntheses and controlled structural transformations of artificial interwoven structures, particularly those exhibiting intricate topologies and bifurcated strands, remain a challenge. In this study, we present a rational design strategy that harnesses the rigidity of tailored ligands in combination with zinc coordination to direct the self‐assembly process, thus enabling the controlled synthesis of covalently linked trefoil perplexane and trefoil tetrahedral knotted cage frameworks. We further elucidate the role of structural rigidity in governing framework transformations, demonstrating two‐way interconversion between interwoven and non‐interwoven architectures, accompanied by tunable guest encapsulation and release. Notably, the incorporation of peripheral crosslinkers was found to lock the cage conformation, thereby regulating its guest binding properties. In addition, the sequence of subcomponent addition was found to be critical to the product outcome: Initial construction of a covalently‐linked knotted cage framework stabilizes kinetic intermediates during self‐assembly, providing access to distinct products.

## Graphical abstract

Systematic control over coordination environments and subcomponent sequences enabled the formation and stabilization of trefoil perplexanes and trefoil tetrahedral knotted cages. Peripheral crosslinkers diminished framework stability to tune host–guest behavior and dynamic exchange, while mechanical entanglement modulated self‐sorting and guest binding, offering routes to future functional designs.

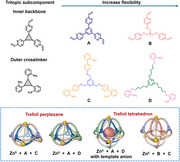

## Introduction

Interwoven structures, such as molecular links and knots, are formed by biological macromolecules, including DNA,^[^ ^1^ ^]^ RNA,^[^ ^2^ ^]^ and proteins,^[^ ^3^ ^]^ where they play crucial roles in biological function and structural stability. Inspired by these natural systems, the synthesis of synthetic interwoven topologies has emerged as a central pursuit in supramolecular chemistry.^[^ ^4^, ^5^, ^6^ ^]^ A key innovation in the use of metal‐ion templation for artificial trefoil knot synthesis was reported by Sauvage and coworkers,^[^ ^7^ ^]^ marking a milestone in the field. Since then, progress has been made in the construction of topologically complex structures, including the use of interwoven grid scaffolds,^[^ ^8^, ^9^ ^]^ hydrophobic‐effect‐driven assembly,^[^ ^10^ ^]^ and anion^[^ ^11^ ^]^ and metal‐ion^[^ ^12^, ^13^, ^14^ ^]^ templation. These approaches have enabled the construction of an increasingly diverse set of artificial interwoven structures, ranging from catenanes^[^ ^15^, ^16^, ^17^, ^18^, ^19^, ^20^, ^21^ ^]^ to molecular knots,^[^ ^5^, ^22^, ^23^, ^24^, ^25^, ^26^, ^27^ ^]^ Borromean rings,^[^ ^28^, ^29^, ^30^, ^31^, ^32^ ^]^ and more complex topologies.^[^ ^33^, ^34^, ^35^, ^36^ ^]^ Many of these architectures show promise for a variety of applications,^[^ ^37^ ^]^ such as ion transport,^[^ ^38^ ^]^ catalysis,^[^ ^39^ ^]^ and the development of materials with useful mechanical and dynamic properties.^[^ ^40^, ^41^, ^42^, ^43^, ^44^, ^45^ ^]^ These achievements underscore the growing relevance of topological control in the design of functional molecular systems.

Metal–organic cages exhibit well‐defined polyhedral geometries^[^ ^46^, ^47^, ^48^, ^49^ ^]^ and have been explored for applications^[^ ^50^, ^51^, ^52^ ^]^ that include chemical separation and purification,^[^ ^53^, ^54^, ^55^ ^]^ drug delivery,^[^ ^56^, ^57^, ^58^ ^]^ catalysis,^[^ ^59^, ^60^, ^61^, ^62^, ^63^, ^64^, ^65^, ^66^, ^67^ ^]^ light harvesting systems,^[^ ^68^, ^69^, ^70^ ^]^ and stabilization of reactive intermediates.^[^ ^71^, ^72^ ^]^ Recently, our group developed two interwoven structures, corresponding to a trefoil perplexane and a trefoil tetrahedron, via a metal–organic cage exterior crosslinking strategy.^[^ ^73^ ^]^ This approach facilitated the efficient assembly of complex topologies, suggesting that further elaboration of ligand design could enable the synthesis of new interwoven structures. Strategies such as employing rigid linkers,^[^ ^74^ ^]^ introducing sterically demanding substituents,^[^ ^75^ ^]^ or applying template effects^[^ ^76^ ^]^ have been developed to address this challenge. Building on these approaches, we hypothesized that tuning the flexibility of face‐capping subcomponents could provide an alternative means of controlling assembly outcomes, a strategy supported by theoretical considerations of entropic changes that demonstrate rigidity‐dependent structural bias. Modulating the flexibility of the face‐capping subcomponents could also enable finer control over transformations between structures as well as over their functional properties.

**Figure 1:**
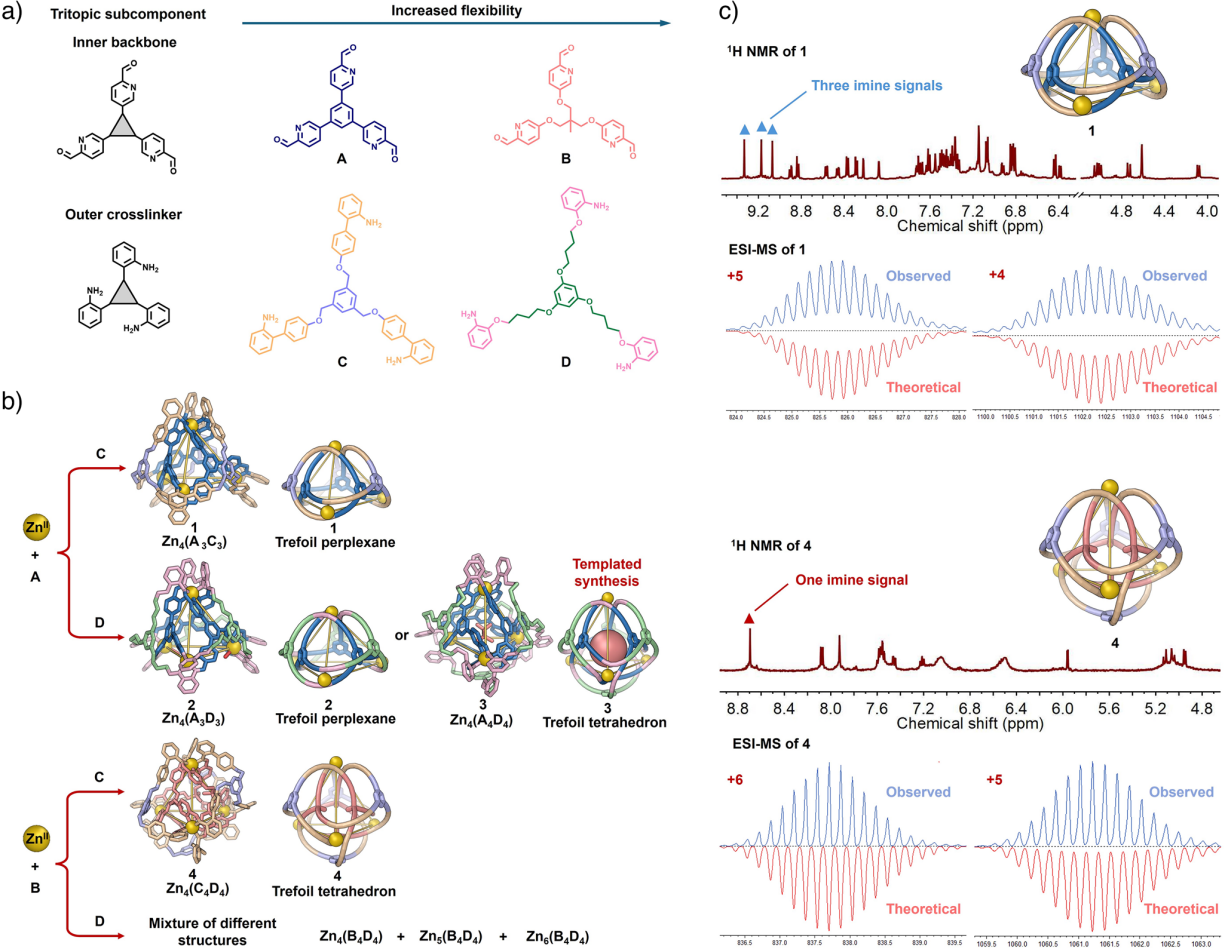
a) Two tris(formylpyridine) (**A** and **B**) and two trianiline (**C** and **D**) subcomponents for the construction of knotted cage frameworks; b) subcomponent self‐assembly of trefoil perplexanes **1** and **2**, and trefoil tetrahedra **3** and **4**; c) ^1^H NMR spectra (500 MHz, CD_3_CN, 298 K) and ESI‐MS of **1** (top) and **4** (bottom).

In this study, a series of tritopic subcomponents exhibiting varying degrees of conformational flexibility were designed: two tritopic formylpyridines (**A** and **B**) and two tris(aniline)s (**C** and **D**), as shown in Figure 1. The different combinations of these subcomponents with each other revealed how they influence the formation of interwoven architectures and the tendencies of these structures to undergo transformation. The structural rigidity and steric demands of individual building blocks were found to modulate the interconversion between discrete assemblies, as well as influence their capacities for guest binding.

## Results and Discussion

Rigid subcomponent **A** (3 equiv.) reacted with moderately flexible subcomponent **C** (3 equiv.) and zinc(II) bis(trifluoromethanesulfonimide) (triflimide or Tf_2_N^–^, 4 equiv.) in acetonitrile at 130 °C in a microwave reactor for 4 h to produce assembly **1** (Figure 1) in 85% yield. Product **1** was characterized by nuclear magnetic resonance (NMR) spectroscopy and electrospray ionization mass spectrometry (ESI‐MS), as shown in Figures S6–S15. Diffusion‐ordered spectroscopy (DOSY) showed that all ^1^H NMR signals exhibited the same diffusion coefficient, indicating that they belonged to a single species (Figure S13) with a solvodynamic radius of 13 Å. The Nuclear Overhauser Effect spectroscopy (NOESY) ^1^H NMR spectrum revealed correlations between the protons on the central phenyl ring of subcomponent **C** and the outward‐facing pyridyl protons of subcomponent **A**, suggesting a double‐layered structural organization (Figure S10).

**Figure 2:**
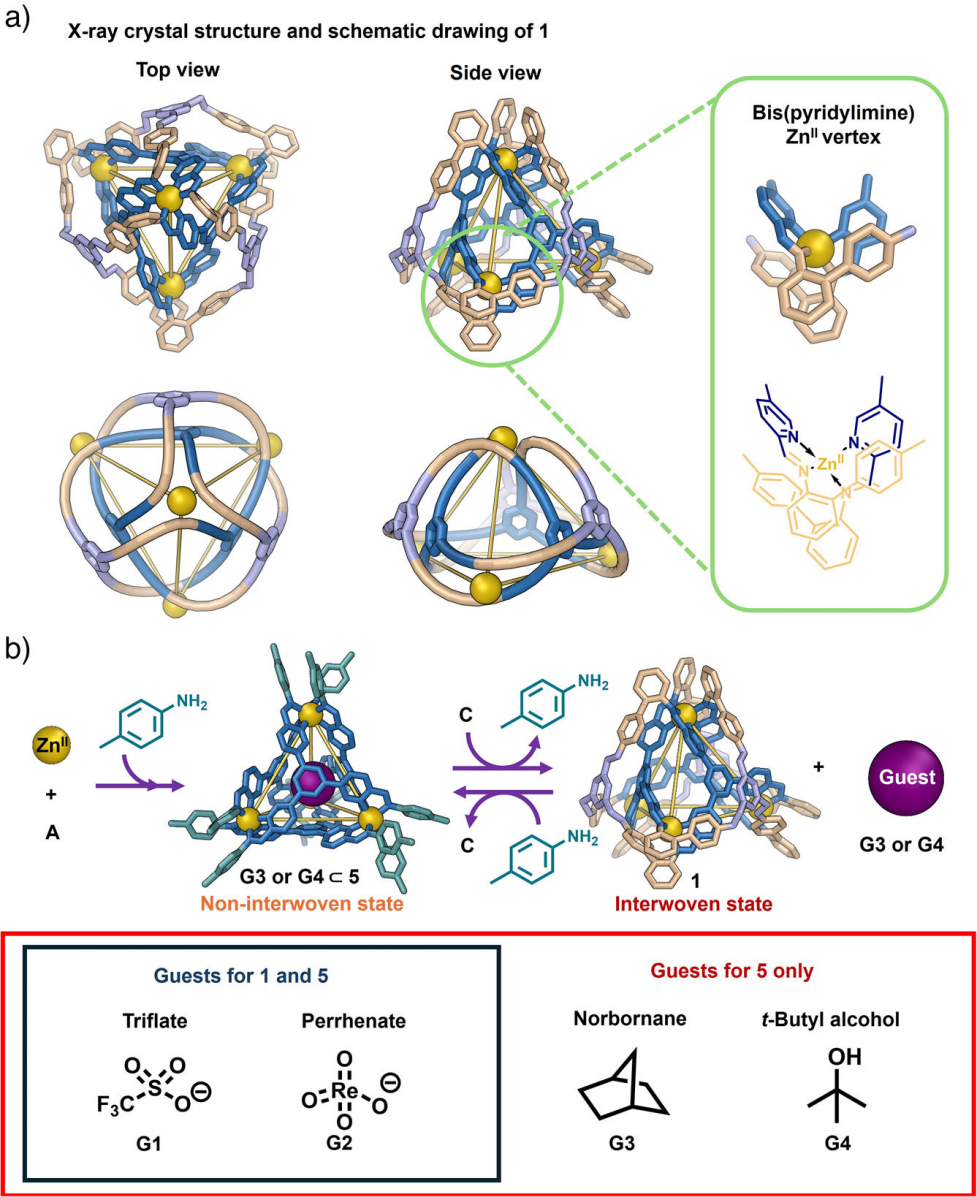
a) X‐ray crystal structure of **1**. Top and side views of **1** are shown to illustrate its topology. Disorder, hydrogen atoms, counterions and solvent molecules of crystallization are omitted for clarity. The structure of **1** contains three distorted tetrahedral bis(pyridylimine) vertices bound to zinc, and one octahedral tris(pyridylimine) vertex, lending it crystallographic *C*_3_ point group symmetry; b) schematic representation of the bidirectional structural transformation between **1** and **5** with controlled guest release, and a list of guests encapsulated within **1** and **5**.

Single‐crystal X‐ray diffraction analysis performed at Diamond Light Source^[^ ^77^ ^]^ revealed a double‐layered trefoil perplexane architecture for **1**, with a single bifurcating ligand strand interwoven around four templating metal centers (Figure 2a). Perplexane **1** incorporates four metal centers with the same handedness, and exhibits crystallographic *C*_3_ symmetry, consistent with the observation of three imine resonances in the ^1^H NMR spectrum (Figure S11). The Zn^II^–Zn^II^ distances ranged from 11.2–11.5 Å. The four Zn^II^ centers include one octahedral tris(pyridylimine) Zn^II^ vertex and three distorted tetrahedral bis(pyridylimine) Zn^II^ vertices. The steric hindrance imparted by the *ortho*‐phenyl group on subcomponent **C**, along with the molecular strain arising from the rigidity of the inner ligands, is inferred to account for the distorted tetrahedral bis(pyridylimine) Zn^II^ vertices. This coordination mode stands in contrast to the octahedral bis(pyridylimine) Zn^II^ trefoil perplexane vertices observed previously.^[^ ^75^ ^]^ The degree of tetrahedral distortion was quantified using the bond angle variance (σ^2^).^[^ ^78^ ^]^ As illustrated in Figure S63, the calculated σ^2^ value of 852 deg^2^ reflects a highly distorted tetrahedral coordination environment.

We explored controlled weaving through subcomponent exchange, developing a bidirectional transformation between interwoven and non‐interwoven structures. Trialdehyde **A** assembled with *p*‐toluidine and zinc(II) triflimide to yield previously‐reported Zn^II^_4_L_4_ tetrahedron **5**.^[^ ^79^ ^]^ Following the addition of *p*‐toluidine (6 equiv. per imine) to **1** and heating to 120 °C for 2 h under microwave irradiation, subcomponent **C** was displaced by *p*‐toluidine, leading to the formation of tetrahedron **5** (Figure S38). Conversely, adding **C** (12 equiv. per cage) to **5** at 120 °C for 2 h under microwave irradiation resulted in the complete displacement of *p*‐toluidine, thus forming the interwoven framework **1** (Figure S39). This bidirectional structural transformation differs markedly from the unidirectional interconversions between metal–organic cages that have been reported before.^[^ ^79^ ^]^

In previous cases,^[^ ^79^ ^]^ the addition of a multitopic amine led to irreversible monoamine displacement, driven by the higher stability imparted by the chelate effect. We attribute the counterintuitive ability of this system to undergo bidirectional transformation to two key factors. First, the inherent rigidity of both subcomponents **A** and **C** minimizes conformational mismatch during structural transformations and thereby avoids byproduct formation.^[^ ^73^ ^]^ Second, steric hindrance at the distorted tetrahedral bis(pyridylimine) Zn^II^ vertices is inferred to diminish the intrinsic stability of the interwoven framework. The distorted Zn^II^ tetrahedral coordination geometry not only reflects strain and weakened metal–ligand orbital overlap, but also generates labile coordination sites that facilitate ligand dissociation and exchange, rendering the Zn^II^ centers thermodynamically and kinetically less stable. As a consequence, the energetic difference between the interwoven **1** and non‐interwoven **5** cage architectures is reduced, facilitating bidirectional structural transformations.

Hosts **1** and **5** exhibited distinct guest‐binding behaviors. As previously reported, both anionic (triflate **G1**, and perrhenate **G2**) and neutral guests (norbornane **G3**, and *t*‐butyl alcohol **G4**) were encapsulated within the cavity of **5** (Figure 2b) in slow exchange on the ^1^H NMR chemical shift timescale.^[^ ^79^ ^]^ During titrations, shifting NMR signals indicated fast‐exchange binding of the anionic guests **G1** and **G2** within **1** (Figures S41 and 43). However, no changes to the ^1^H NMR spectrum of **1** were observed following the addition of the neutral guests **G3** and **G4** (Figure S40), which was attributed to the lower degree of enclosure of **1**, one face of which is uncovered by ligands. This structural feature of **1** likely facilitates the binding of larger anionic guests, such as Tf_2_N^–^, relative to **5**, while also promoting rapid anionic guest exchange on the NMR timescale and diminishing the binding affinity toward neutral guests.

The crystal structure of **1** showed that a Tf_2_N^–^ counter ion also bound within its basket cavity (Figure S61). The competitive binding constants of **G1** and **G2** to **1** were calculated via NMR titrations to be (4.7 ± 0.1) × 10^2^ M^−1^ and (8.9 ± 0.9) × 10^2^ M^−1^, respectively, with guest‐binding ratios of 1:1 (Figures S42 and S44). The competitive binding of Tf_2_N^−^ anions could also play a role in the lack of binding observed for the neutral guests **G3** and **G4**.

Synthetic receptors may exhibit stimuli‐responsive behaviors that allow reorganization of their structures to facilitate guest uptake and release.^[^ ^50^, ^80^, ^81^ ^]^ The addition of *p*‐toluidine to **1** in the presence of unbound **G3** or **G4** prompted such a structural reorganization, which resulted in the formation of **5**, which then encapsulated **G3** or **G4**. ^1^H NMR spectra indicated the formation of the host–guest complex **G3**⊂**5** or **G4**⊂**5** (Figures S46 and S49), as also confirmed by ESI‐MS (Figures S47 and S50). In contrast, the addition of tritopic subcomponent **C** to **G3**⊂**5** or **G4**⊂**5** prompted the reverse transformation, which resulted in the formation of **1** and release of **G3** or **G4**, as confirmed by ^1^H NMR (Figures S45 and S48).

As reported,^[^ ^73^ ^]^ subcomponent **A** (3 equiv.), flexible exterior crosslinking subcomponent **D** (3 equiv.), and Zn^II^ (4 equiv.) assembled into a trefoil perplexane framework. In contrast, the presence of excess (20 equiv.) TfO^−^ promoted the exclusive formation of the trefoil tetrahedron. This outcome is attributed to a shift in the thermodynamic landscape of the assembly process, where TfO^−^ acts as a templating agent to direct the formation of the closed structure. A comparison of the crystal structures of perplexanes **2** ^[^ ^73^ ^]^ and **1** reveals that the molecular strain at the three bis(pyridylimine) vertices is markedly reduced, with Zn^II^ centers adopting an octahedral configuration, which contrasts with the distorted tetrahedral coordination observed in **1** (Figure S62). We hypothesized that increasing the flexibility of the exterior crosslinking moieties could allow more effective relief of strain at the Zn^II^ vertices and alleviate spatial constraints on the arrangement of internal ligands, thereby facilitating the construction of topologically interwoven architectures. Although the incorporation of a flexible outer crosslinker renders the formation of a trefoil tetrahedron feasible, the construction of such a topology in this system still necessitates a templating guest. Therefore, achieving template‐free assembly of the trefoil tetrahedron remained a significant challenge, warranting further optimization of ligand design.

**Figure 3:**
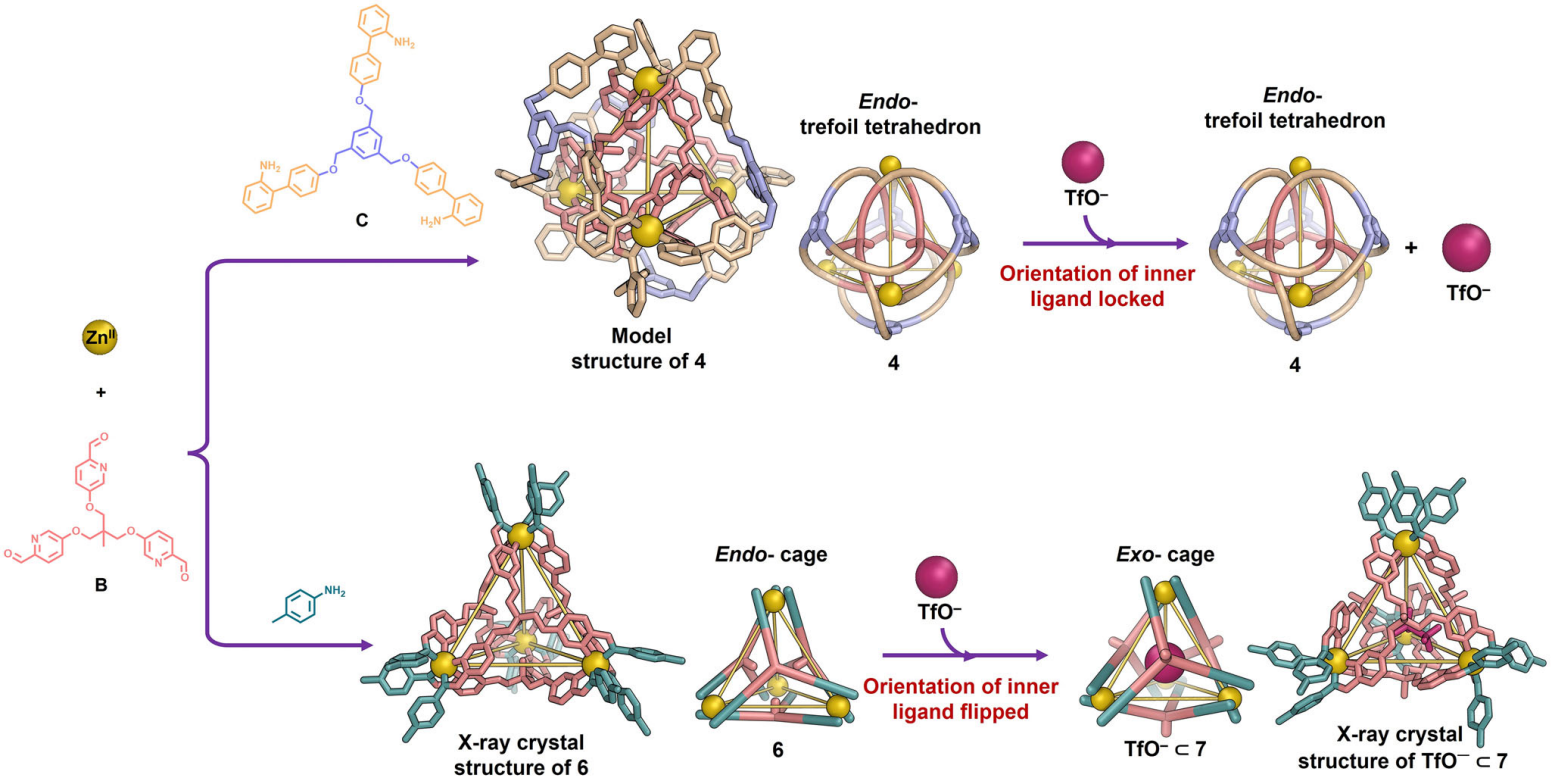
**Top**, the exterior crosslinking of **4** prevents its faces from inverting, which would be required for the binding of triflate. **Bottom**, the methyl groups of the **B** residues within **6** invert from inward‐facing to outward‐facing in order to provide sufficient room to bind triflate. The X‐ray crystal structure of **6**, TfO^–^⊂**7**, and the GFN2‐xTB^[^ ^82^ ^]^ optimized structure of **4** are each shown in both stick and schematic representations.

A more conformationally flexible tritopic formylpyridine subcomponent **B** (Figure 3) was therefore prepared. Exterior crosslinking subcomponent **C** assembled with zinc(II) triflimide and subcomponent **B**, affording template‐free synthesis of trefoil tetrahedron **4**, as confirmed by ^1^H NMR spectroscopy and ESI‐MS (Figures S16–S25). The ^1^H NMR and ^1^H‐^13^C HSQC spectra of **4** revealed a single imine resonance, consistent with a highly symmetric structure (Figure S21). Its ^1^H NOESY spectrum exhibited through‐space correlations between the central moieties of subcomponents **B** and **C**, suggesting a double‐layered architecture (Figure S20). ESI‐MS analysis confirmed the formation of a Zn_4_(**B**_4_ **C**_4_) assembly, with the same stoichiometry as trefoil tetrahedron **3**. The DOSY NMR spectrum of **4** (Figure S23) revealed an identical diffusion coefficient for all ^1^H signals, corresponding to a solvodynamic radius of 14 Å. This value was consistent with the geometry‐optimized structure of **4**, obtained at the GFN2‐xTB level^[^ ^82^ ^]^ (Figure S23).

The reaction of *p*‐toluidine with subcomponent **B** and zinc(II) triflimide yielded tetrahedral Zn^II^_4_L_4_ cage **6**, where the central methyl substituents are directed toward the internal cavity. This work thus builds upon previous studies^[^ ^83^ ^]^ in which this structural form was not accessible under the reported conditions. The formation and geometry of this assembly were confirmed by single‐crystal X‐ray diffraction, ESI‐MS, and NMR spectroscopy, as shown in Figures 3 and S26–S35. Following the addition of TfO^–^ (8 equiv.), the ligands underwent adaptive conformational inversion, resulting in an outward reorientation of the methyl groups to enable guest encapsulation, forming TfO^–^⊂**7**, as shown in Figures 3, S51, and S52. The formation of host–guest complex TfO^–^⊂**7** was confirmed by ^19^F NMR spectroscopy, which showed two distinct signals for free and encapsulated TfO^–^(Figure S52). The reported X‐ray crystal structure of TfO^–^⊂**7** ^[^ ^83^ ^]^ is shown in Figure 3.

The same anionic guest, TfO^–^ (16 equiv.), was added to a solution of **4**. However, the exterior crosslinking of the trefoil tetrahedron framework prevented its internal ligands from undergoing adaptive conformational inversion. Thus, the added triflate did not result in the binding of this guest within **4** (Figures S53 and S54), even after heating at 50 °C for 12 h, with all four methyl substituents remaining oriented toward the internal cavity. Crosslinking thus deactivated the modulation of ligand conformational dynamics that enabled guest binding within the cage framework.

The self‐assembly behavior of the two most flexible subcomponents, **B** and **D**, was also investigated, as shown in Figure 1b. Upon reaction with zinc(II) triflimide, **B** and **D** produced a complex mixture of products, as reflected in an NMR spectrum that exhibited multiple sharp and broad signals (Figure S36). ESI‐MS revealed the presence of at least three distinct assemblies (Figure S37), suggesting that the simultaneous incorporation of both flexible subcomponents may lead to different crosslinked configurations, as opposed to a single well‐defined structure.

**Figure 4:**
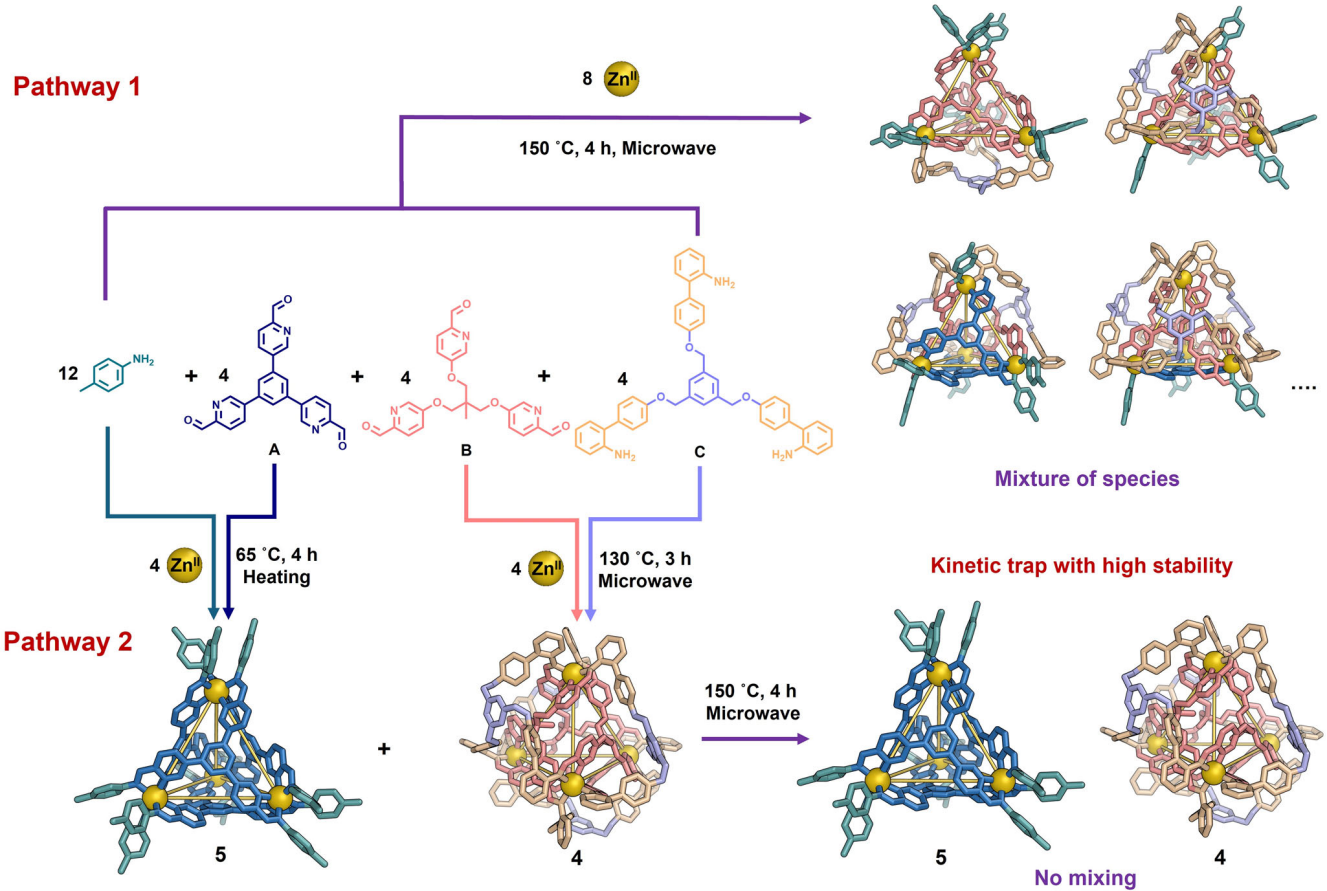
Schematic illustration showing how different assembly pathways lead to distinct products. As shown in **pathway 1**, following the mixture of the four subcomponents shown with Zn^II^, a diverse mixture of species is formed. **Pathway 2** illustrates that structures **4** and **5** did not exchange subcomponents once formed, indicating that they are kinetically locked.

We explored whether the sequence of subcomponent assembly could influence the product outcome, particularly given our observation that exterior crosslinking may enhance the stability of the cage framework. As shown in Figure 4 pathway 1, when subcomponents **A**, **B**, **C**, and *p*‐toluidine were mixed with Zn^II^ and subjected to microwave irradiation at 150 °C for 4 h, a complex mixture with both sharp and broad signals was observed in the NMR spectrum, indicative of a structurally heterogeneous product. ESI‐MS analysis revealed four distinct species corresponding to a mixture of heteroleptic structures (Figures 4, S55, and S56).

In contrast, as shown in Figure 4 (pathway 2), when **4** and **5** were preassembled from the same set of subcomponents and subsequently subjected to identical microwave conditions (150 °C, 4 h), these two structures were not observed to exchange subcomponents (Figure S57). Notably, under microwave heating at 150 °C for 12 h, **5** decomposed, whereas **4** remained (Figure S58). In a control experiment, the mixture of tetrahedral cages **5** and **6** exposed to microwave heating at 150 °C for 4 h led to the complete decomposition of **6** (Figure S59). We further examined whether **4** exhibits greater structural stability than its congener **6** by testing its resistance to the chemical stimulus DMSO, which can competitively coordinate to Zn^II^ centers and disrupt the framework. As shown in Figure S60, the addition of 45 µL of deuterated DMSO to **6** led to its complete decomposition. However, its knotted counterpart **4** remained entirely intact upon exposure to the same amount of DMSO. These findings indicate that the interweaving of **4** not only enhanced the structural stability of the cage framework, but also effectively locked the system into a kinetic trap. Interweaving thus represents a viable strategy for imposing kinetic control in structurally complex self‐assembled architectures.

## Conclusion

Systematic manipulation of both the metal coordination environment and the subcomponent addition sequence thus enabled precise control over the formation and stabilization of topologically complex structures, including trefoil perplexanes and trefoil tetrahedral knotted cages. Incorporating flexible subcomponents allows more effective relief of strain at the Zn^II^ vertices and reduces steric constraints on ligand arrangement, thereby favoring the formation of tris(pyridylimine) vertices and promoting trefoil tetrahedron assembly. In contrast, the use of more rigid, sterically demanding subcomponents increases vertex strain, favoring bis(pyridylimine) vertices and thus biasing the system toward trefoil perplexane formation. Additionally, the incorporation of peripheral crosslinkers, which are inferred to diminish the intrinsic stability of the interwoven framework, enabled tunable host–guest properties and modulated dynamic exchange processes. Our work thus establishes how mechanical entanglement can modulate the ability of supramolecular systems to rearrange, thus altering their self‐sorting properties along with their abilities to bind guests. Future developments may enable further useful functional alterations to host‐guest systems.

## Author Contributions

J.R.N. and Y.Y. conceived the project and designed the experiments. Y.Y. performed the experiments and analyzed the data. S.Y.H. helped with the large‐scale synthesis of subcomponent C and with manuscript revision. T.K.R. collected and refined the crystal structure. P.C.P.T. and D.J.W. performed the GFN2‐xTB calculations. S.G. helped with the one‐step synthesis of subcomponent **C**. A.W.H. analyzed the structures and edited the manuscript. J.R.N. is the principal investigator.

## Conflict of Interests

The authors declare no conflict of interest.

## Supporting information



Supporting Information

## Data Availability

The data that support the findings of this study are available in the Supporting Information of this article.
