# Supplementary material for: Design and Structural Transformations of Zinc(II) Knotted Cage Frameworks
Source: Angew Chem Int Ed Engl. 2025 Oct 16;64(50):e202519491. doi: 10.1002/anie.202519491 (PMC12684317; doi:10.1002/anie.202519491)
Supplement: Supplementary file 1 — Supporting Information [file ANIE-64-e202519491-s001.docx]

Supporting Information for

Design and Structural Transformations of Zinc(II) Knotted Cage Frameworks

Yuchong Yang, Sabrina Y. Hu, Tanya K. Ronson, Paula C.P. Teeuwen, Sudhakar Gaikwad,

Andrew W. Heard, David J. Wales, Jonathan R. Nitschke*

Yusuf Hamied Department of Chemistry, University of Cambridge, Lensfield Road, Cambridge, UK, CB2 1EW

**Table of Contents**

[1. General 3](#_Toc203726949)

[2. Subcomponent synthesis and characterisation 4](#_Toc203726950)

[3. Self-assembly of coordination architectures 9](#_Toc203726951)

[3.1 Self-assembly of **1** 9](#_Toc203726952)

[3.2 Self-assembly of **2**, **3** and **4** 17](#_Toc203726953)

[3.3 Self-assembly of tetrahedral frameworks **5**, **6** and TfO^–^⊂**7** 25](#_Toc203726954)

[3.4 Self-assembly between Subcomponent **B** and **D** 32](#_Toc203726955)

[4. Structural transformations between 1 and 5 34](#_Toc203726956)

[4.1 Structural transformation from **1** to **5** 34](#_Toc203726957)

[4.2 Structural transformation from **5** to **1** 35](#_Toc203726958)

[5. Host-guest investigations of 1 36](#_Toc203726959)

[5.1 General methods 36](#_Toc203726960)

[5.2 NMR spectra from host-guest investigations 37](#_Toc203726961)

[6. Guest release and encapsulation induced by structural transformations between 1 and 5 41](#_Toc203726962)

[6.1 Release and encapsulation of **G3** via structural transformations between **1** and **5** 41](#_Toc203726963)

[6.2 Release and encapsulation of **G4** via structural transformations between **1** and **5** 43](#_Toc203726964)

[7. Structural transformation of 4 and 6 45](#_Toc203726965)

[7.1 Triflate induced ligand reorientation in **6** 45](#_Toc203726966)

[7.2 Addition of triflate to **4**. 46](#_Toc203726967)

[8. Self-assembly between Subcomponents A, B, C and *p*-toluidine 47](#_Toc203726968)

[9. X-ray Crystallography 52](#_Toc203726969)

[10. References 57](#_Toc203726970)

# General

**Materials and Methods**

Unless otherwise stated, all chemicals were obtained from commercial sources and used as received. Subcomponents **A**,^1^ **B**^2^ and **D**^3^ were prepared according to literature procedures.

**Nuclear Magnetic Resonance (NMR)**

NMR experiments were measured on Bruker AVANCE III and NEO (400 and 500 MHz) spectrometers. Chemical shifts for ^1^H and ^13^C NMR are reported in ppm with residual solvent as reference: Acetonitrile (1.94 ppm for ^1^H, 1.32 ppm for ^13^C). Abbreviations for signal multiplicity of ^1^H NMR spectra are shown as following: s: singlet, d: doublet, t: triplet, dd: doublet of doublets; dt: doublet of triplets; m: multiplet, br: broad.

**Microwave** **Synthesizer**

The microwave-assisted reaction was conducted using a CEM Discover SP synthesizer. After 10 seconds of sonication, the reaction mixture was directly transferred into the microwave synthesizer and heated to the target temperature. Electromagnetic stirring was applied at a medium speed setting. During the reaction, the power output was approximately 90 W, and the pressure reached ~80 psi.

**Mass Spectrometry (MS)**

Low-resolution electrospray ionization mass spectrometry (LR ESI-MS) was undertaken on a Waters XevoTQD (cone voltage 5-20 eV; desolvation temperature 307 K; ionization temperature 325 K), infused from a Harvard syringe pump at a rate of 10 μL∙min^−1^. High-resolution electrospray ionization mass spectrometry (HR ESI-MS) was undertaken on a Waters Synapt G2-Si mass spectrometer.

**Theoretical Calculations**

Semi-empirical quantum mechanical calculations were carried out using the OPTIM^4,5^ program, which contains a wide variety of geometry optimization tools for locating stationary points on potential energy surfaces and calculating reaction pathways. Geometry optimizations were performed on the structures **4** and mixture of species **8-11** in Section 8 using the OPTIM interface to the *xtb* program, which was used to calculate the energy and gradient at the GFN2-xTB level^6,7^ of theory. Implicit MeCN solvation was included for structure **4**. A charge of +8, 0 and 0 was included for structures **4** and **8-11** respectively. The *xtb* accuracy parameter, which determines integral screening thresholds and convergence of the self-consistent charge procedure, was set to 0.1. Input structures for these calculations were produced using the MM3 force field in the SCIGRESS^8^ software (Fujitsu Limited, Tokyo, Japan, 2013) version FJ 2.6 (EU 3.1.9) Build 5996.8255.20141202. Well converged minima were obtained.

# Subcomponent synthesis and characterization


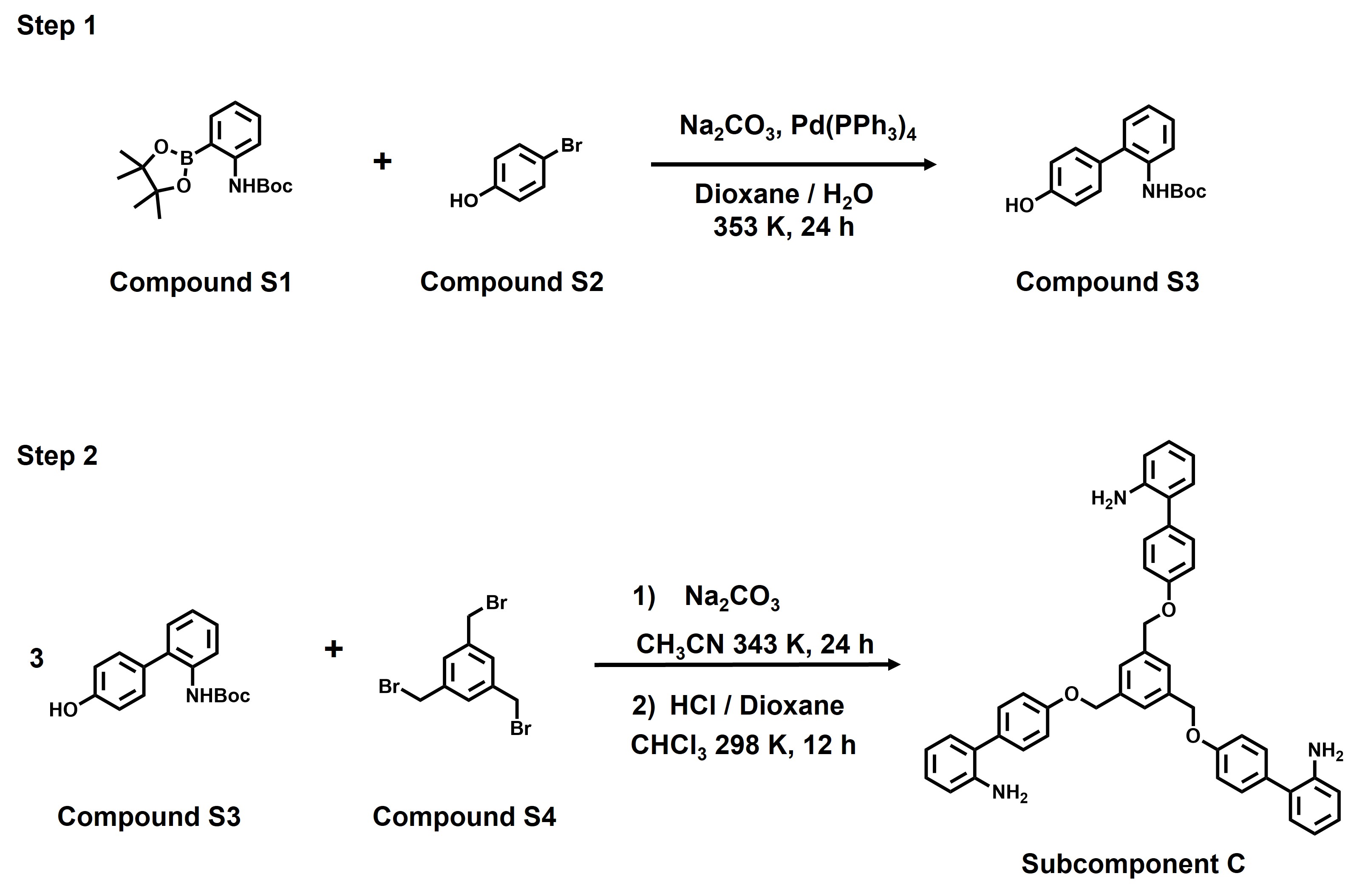


**Scheme S1.** Synthesis of subcomponent **C**.

**Step 1:**

To aSchlenk flaskwere addedcompound **S1** tert-butyl N-[2-(4,4,5,5-tetramethyl-1,3,2-dioxaborolan-2-yl)phenyl]carbamate (1.00 g, 3.1 mmol, 1.0 equiv.), compound **S2** 4-bromophenol (542 mg, 3.13 mmol, 1.0 equiv.), and Na_2_CO_3_ (458 mg, 4.32 mmol, 1.4 equiv.). Then 1,4-dioxane (12 mL) and water (4 mL) were added to the mixture to give a dark yellow suspension. After two freeze-pump-thaw cycles, *tetrakis*(triphenylphosphine)palladium(0) Pd(PPh_3_)_4_ (120 mg, 0.10 mmol, 0.03 equiv.) crystalline yellow powder was carefully added while the mixture was frozen and the reaction system was degassed again. The resulting mixture was then heated at 80 °C under nitrogen for 24 h., cooled to room temperature and the solvent was removed under reduced pressure, and then 30 mL CH_2_Cl_2_ and 30 mL water were added. The aqueous layer was extracted three times with 10 mL CH_2_Cl_2_ and then washed with 40 mL brine. The organic phases were combined and dried over Na_2_SO_4_, filtered, and concentrated under reduced pressure. The crude product was purified by silica gel column chromatography with EtOAc/Hexane (v/v 1:10 to 3:1) to give compound **S3** as a white solid (411 mg, 46%).

**^1^H NMR (700 MHz, CDCl_3_)** δ8.10 (d, *J* = 4.8 Hz, 1H), 7.34 (ddd, *J* = 8.5, 7.5, 1.7 Hz, 1H), 7.27 (d, *J* = 8.5 Hz, 2H), 7.19 (dd, *J* = 7.6, 1.6 Hz, 1H), 7.10 (td, *J* = 7.5, 1.3 Hz, 1H), 6.97 (d, *J* = 8.5 Hz, 2H), 6.51 (s, 1H), 5.05 (s, 1H) , 1.50 (s, 9H).

**^13^C NMR (176 MHz, CDCl_3_)** δ 155.2, 153.0, 135.4, 131.0, 130.7, 130.7, 130.2, 128.1, 123.0, 119.7, 115.9, 80.5, 28.3.

**ESI-MS (CH_3_CN)** *m*/*z*: [M+H]^+^ calc. for 286.14, found 286.14.


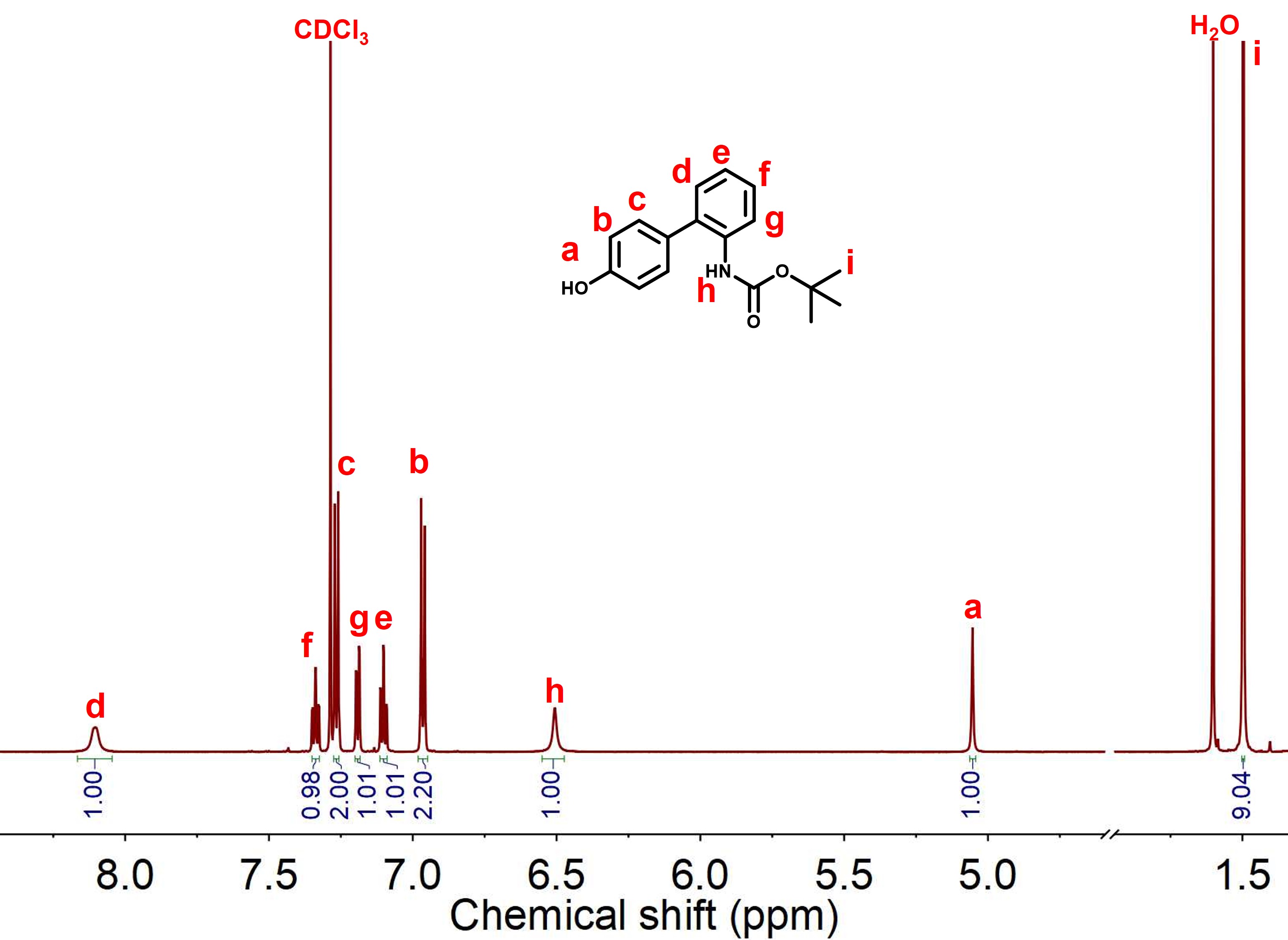


**Figure S1*.*** ^1^H NMR spectrum (700 MHz, 298K, CDCl_3_) of compound **S3**.


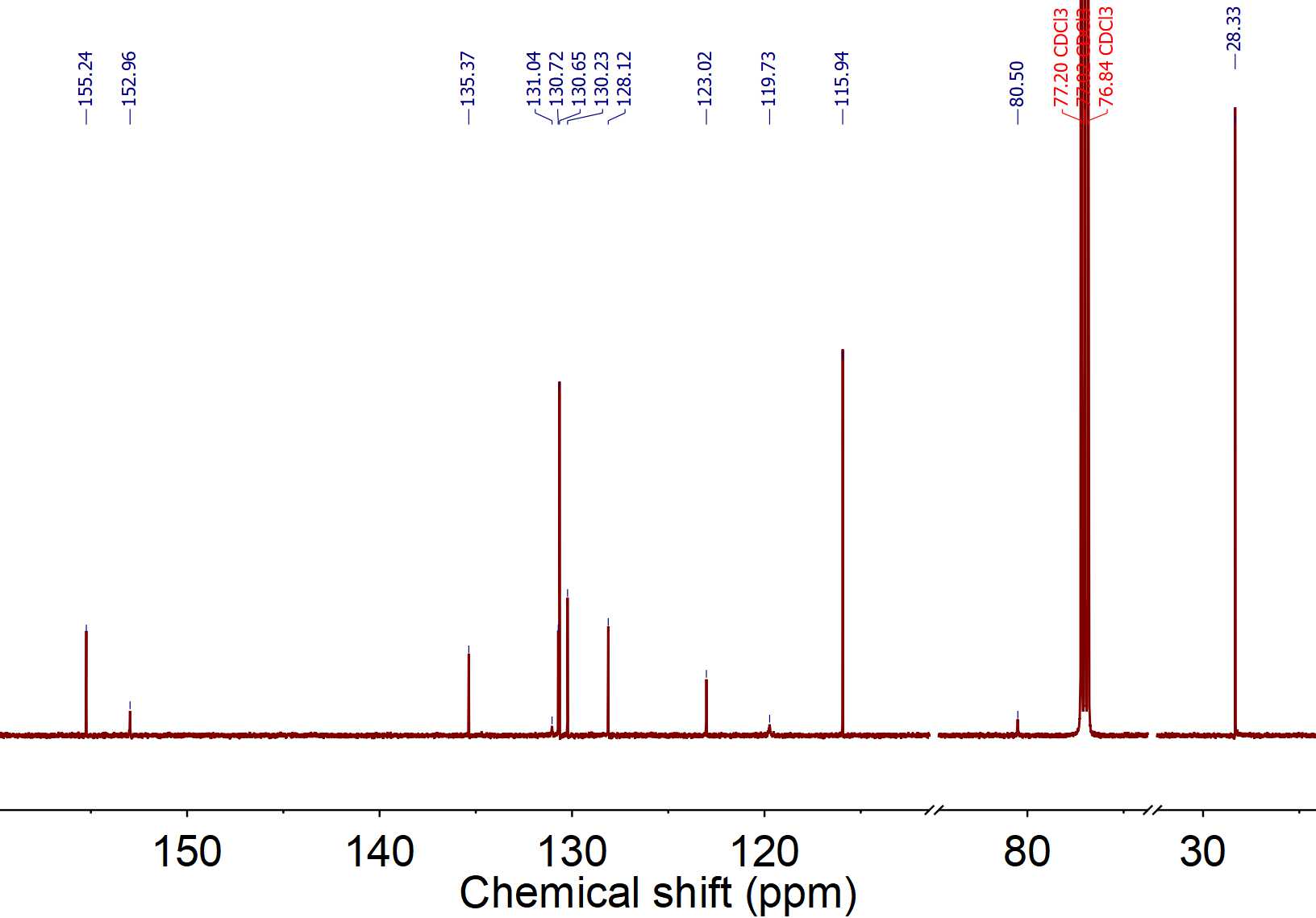


**Figure S2.** ^13^C NMR spectrum (176 MHz, 298K, CDCl_3_) of compound **S3**.

**Step 2:**

To aSchlenk flaskwere addedcompound **S3** (400 mg, 1.41 mmol, 3 equiv.), compound **S4** 1,3,5-tris(bromomethyl)benzene (167 mg, 0.47 mmol, 1 equiv.), and Na_2_CO_3_ (297 mg, 2.8 mmol, 6.0 equiv.). Then acetonitrile (20 mL) was added to the mixture. The resulting mixture was then heated at 70 °C for 24 h. The solvent was removed under reduced pressure, and then 80 mL CH_2_Cl_2_ and 50 mL water were added. The aqueous layer was extracted three times with 15 mL CH_2_Cl_2_ and then washed with 40 mL brine. The organic phases were combined and dried over Na_2_SO_4_, filtered, and concentrated under reduced pressure. The crude product was redissolved in a mixture of chloroform (10 mL) and 3 mL of 1 M HCl in 1,4-dioxane. The solution was stirred for 12 hours. Afterward, the reaction mixture was neutralized with NaHCO_3_, followed by the addition of 50 mL CH_2_Cl_2_ and 30 mL water. The organic layer was separated, concentrated under reduced pressure, and recrystallized from CH_2_Cl_2_/hexane to give subcompound **C** as a white solid (235 mg, 75%).

**^1^H NMR (500 MHz, CD_2_Cl_2_)** δ 7.60 (s, 3H), 7.44 – 7.40 (d, *J* = 8.7 Hz 6H), 7.17 – 7.09 (m, 12H), 6.83 – 6.76 (m, 6H), 5.22 (s, 6H), 3.81 (s, 6H).

**^13^C NMR (126 MHz, CD_2_Cl_2_)** δ 157.9, 143.9, 138.1, 132.3, 130.3, 130.2, 128.1, 127.0, 126.1, 118.3, 115.3, 115.1, 69.8.

**ESI-MS (CH_3_CN)** *m*/*z*: [M+H]^+^ calc. for, 670.31, found 670.31.


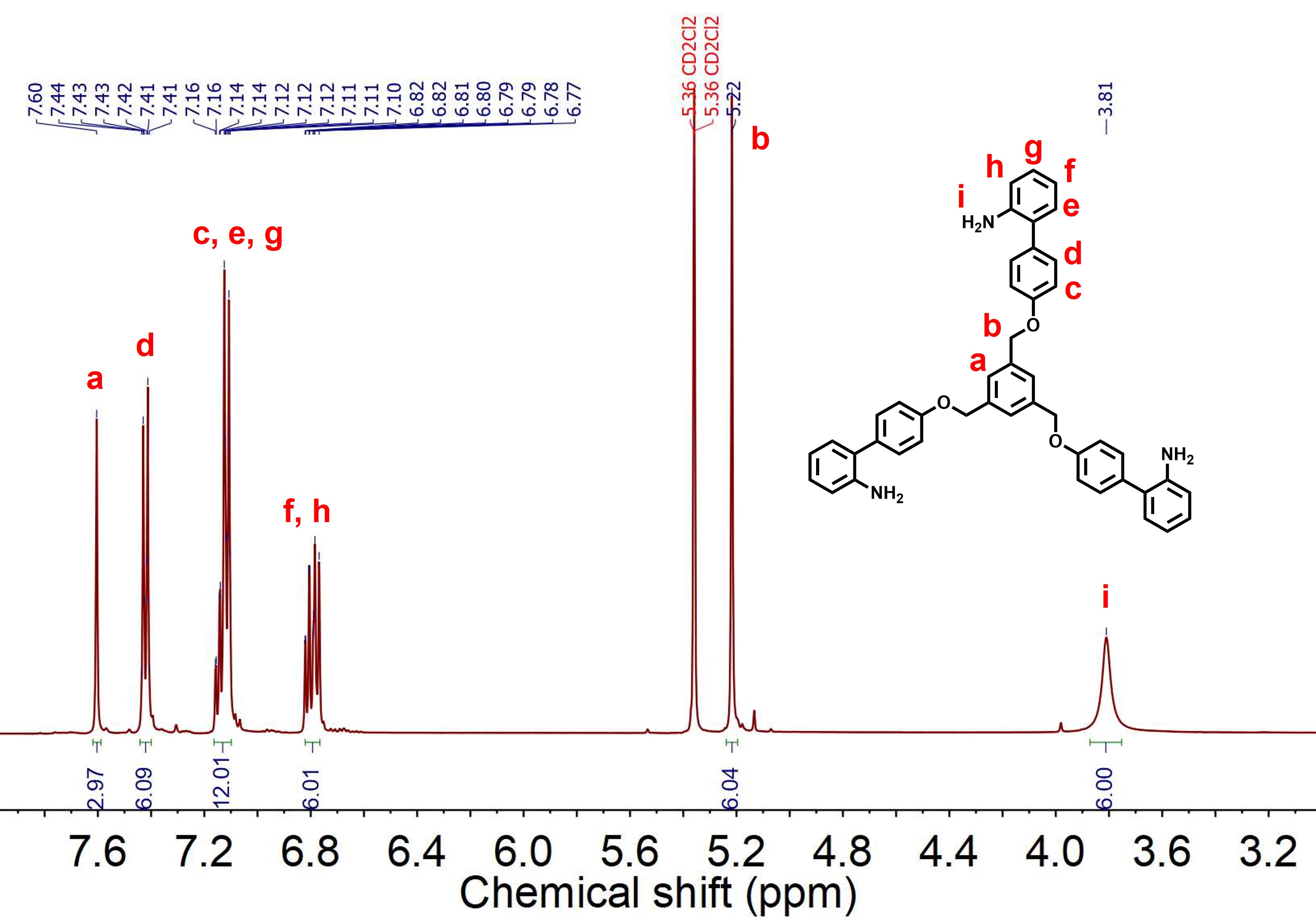


**Figure S3.** ^1^H NMR spectrum (500 MHz, 298K, CD_2_Cl_2_) of subcomponent **C**.


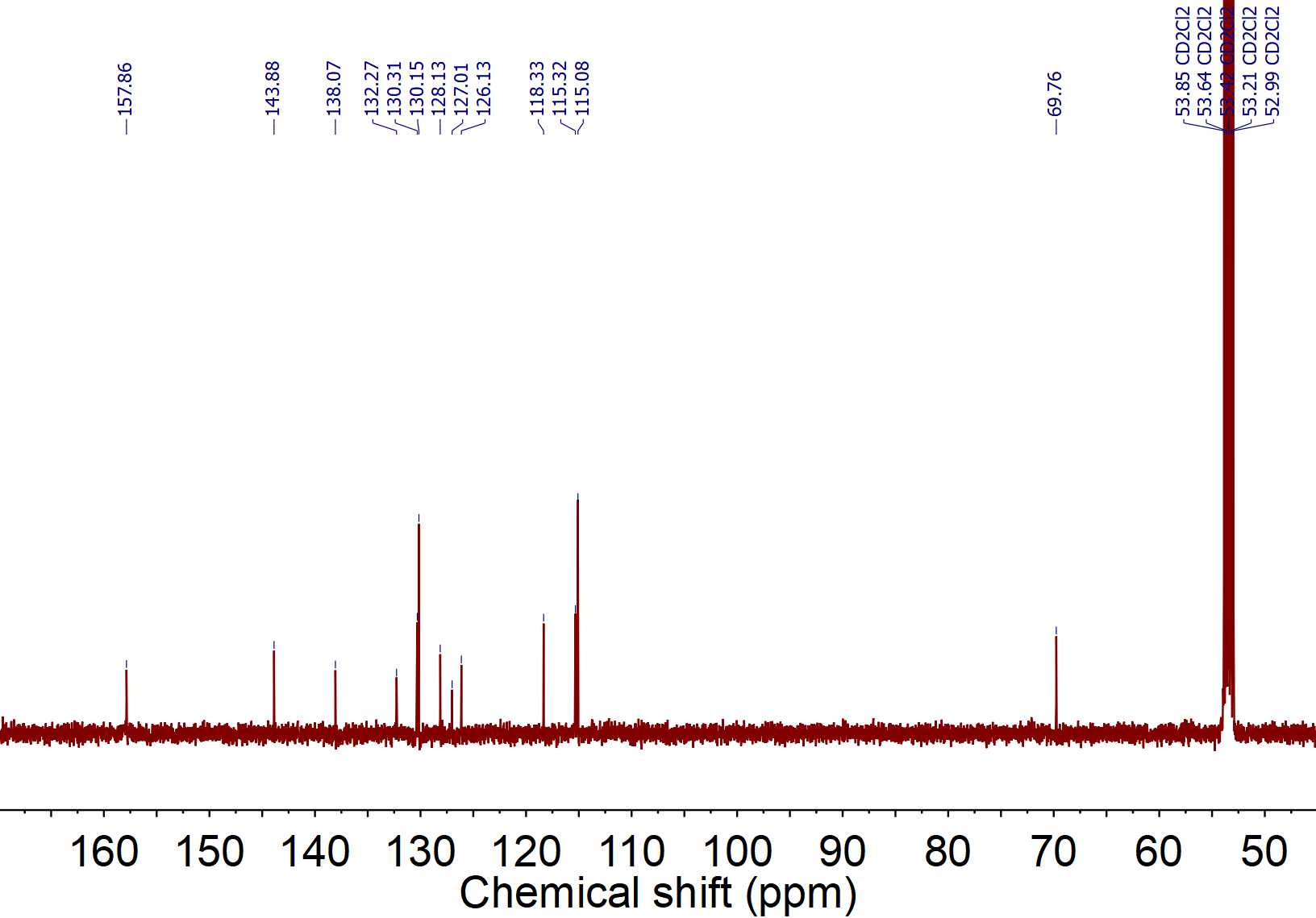


**Figure S4.** ^13^C NMR spectrum (126 MHz, 298K, CD_2_Cl_2_) of subcomponent **C**.


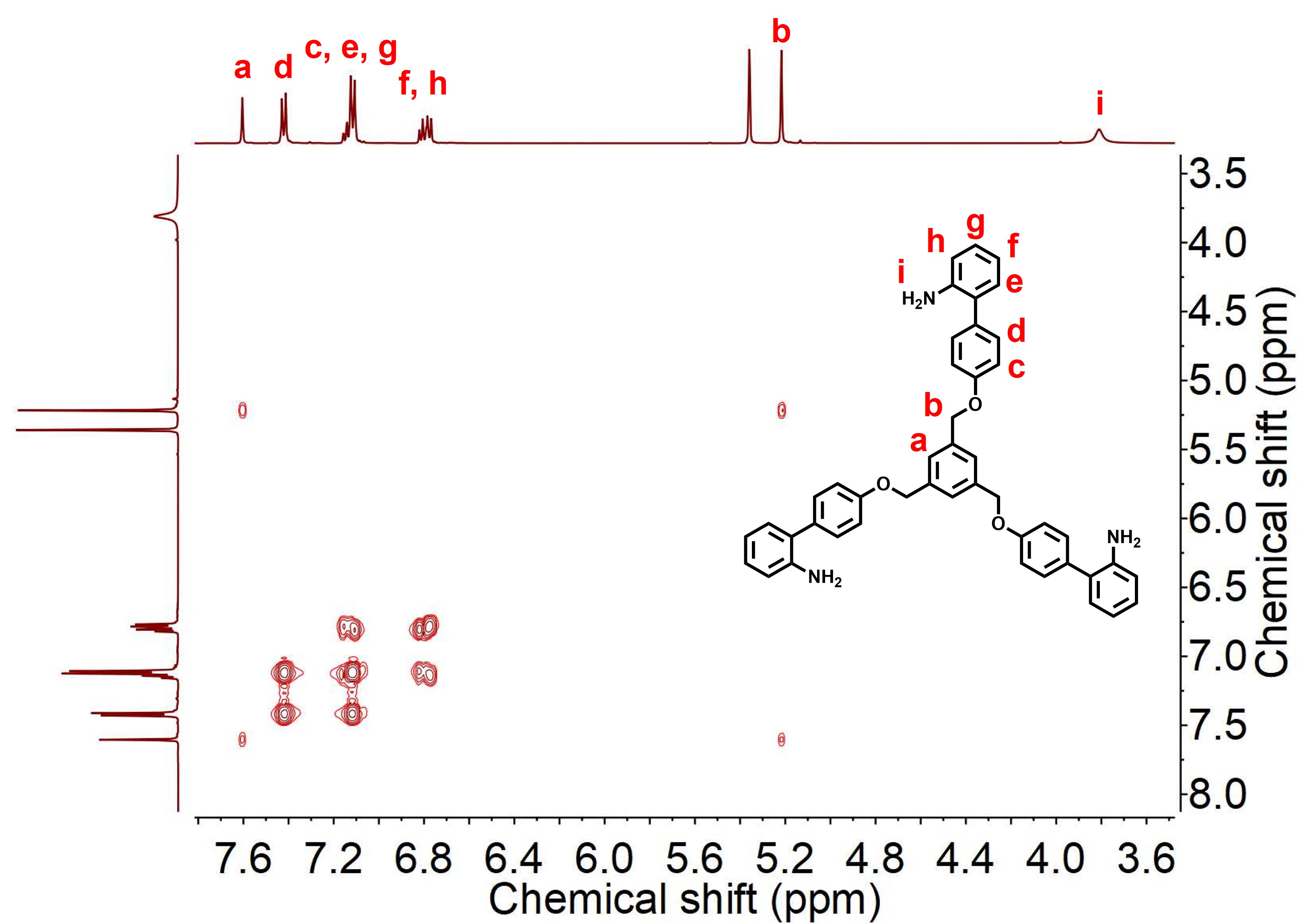


**Figure S5**. ^1^H-^1^H COSY NMR spectrum (500 MHz, 298K, CD*_2_*Cl*_2_*) of subcomponent **C**.

# Self-assembly of coordination architectures

3.1 Self-assembly of **1**


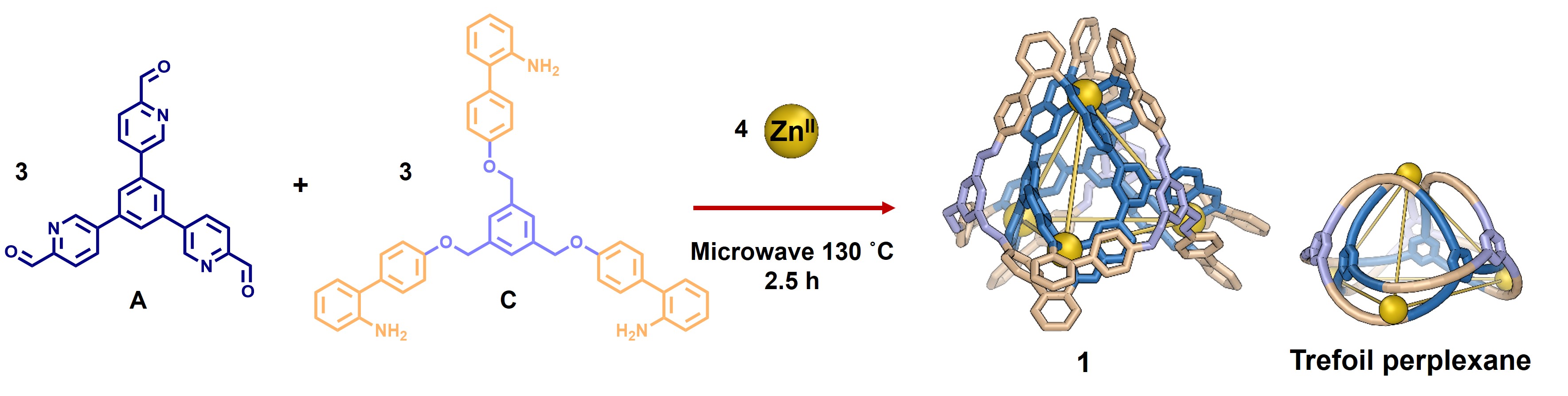


**Scheme S2.** Construction of **1**

Subcomponent **A** (5.0 mg, 12.7 µmol, 1.0 equiv.), Subcomponent **C** (8.52 mg, 12.7 µmol, 1.0 equiv.) and zinc(II) bis(trifluoromethylsulfonyl)imide (11.1 mg, 17.8 µmol, 1.4 equiv.) were dissolved in acetonitrile (2 mL). The reaction mixture was heated and stirred in microwave reactor for 2.5 h at 130 ˚C. Then the mixture was concentrated to 0.5 mL and diethyl ether (14 mL) was added. The precipitate was collected by centrifugation and washed two times with excess diethyl ether to give **1** in 85 % yield.

**^1^H NMR (500 MHz, CD_3_CN)** δ9.33 (s, 3H), 9.17 (s, 3H), 9.07 (s, 3H), 8.90 (dd, *J* = 8.1, 2.0 Hz, 3H), 8.83 (d, *J* = 8.2 Hz, 3H), 8.57 (dd, *J* = 8.0, 2.2 Hz, 3H), 8.46 (dd, *J* = 8.5, 2.3 Hz, 3H), 8.37 (d, *J* = 8.1 Hz, 3H), 8.29 (d, *J* = 8.3 Hz, 3H), 8.22 (d, *J* = 1.9 Hz, 3H), 8.08 (d, *J* = 2.2 Hz, 3H), 7.74 – 7.59 (m, 15H), 7.55 (d, *J* = 1.7 Hz, 3H), 7.53 – 7.32 (m, 39H), 7.15 (s, 9H), 7.09 – 7.05 (m, 12H), 6.92 (dd, *J* = 8.3, 2.4 Hz, 3H), 6.84 (d, *J* = 8.8 Hz, 6H), 6.82 (d, *J* = 9.0 Hz, 6H), 6.46 – 6.42 (m, 6H), 6.39 (dd, *J* = 7.9, 1.3 Hz, 3H), 5.04 (d, *J* = 12.6 Hz, 3H), 5.00 (d, *J* = 9.6 Hz, 3H), 4.73 (d, *J* = 12.5 Hz, 3H), 4.61 (s, 6H), 4.09 (d, *J* = 9.8 Hz, 3H).

**^13^C NMR (126 MHz, CD_3_CN)** δ 167.2, 162.5, 160.4, 159.4, 159.1, 157.0, 148.1, 146.3, 146.3, 145.8, 145.3, 144.6, 143.9, 143.2, 141.9, 140.7, 140.3, 139.2, 138.5, 137.5, 137.4, 137.0, 136.7, 136.5, 135.5, 135.5, 134.6, 133.4, 133.3, 133.2, 132.5, 131.4, 131.1, 131.0, 130.8, 130.5, 130.2, 129.8, 129.6, 129.1, 128.2, 128.0, 127.9, 127.3, 126.9, 126.3, 126.2, 124.6, 124.3, 122.4, 121.8, 120.9, 119.8 (q, ^1^*J*_CF_ = 320.6 Hz, Tf_2_N^–^), 118.9, 117.3, 115.1, 70.0, 69.7, 69.3.

**^19^F NMR (376 MHz, CD_3_CN)** δ –79.98 (s, CF_3_).

**ESI-MS (CH_3_CN) for 1:** *m*/*z* = 510.0 [**1**·(Tf_2_N^–^)_1_]^7+^,641.7 [**1**·(Tf_2_N^–^)_2_]^6+^,826.1 [**1**·(Tf_2_N^–^)_3_]^5+^,1102.7 [**1**·(Tf_2_N^–^)_4_]^4+^, 1563.9 [**1**·(Tf_2_N^–^)_5_]^3+^.


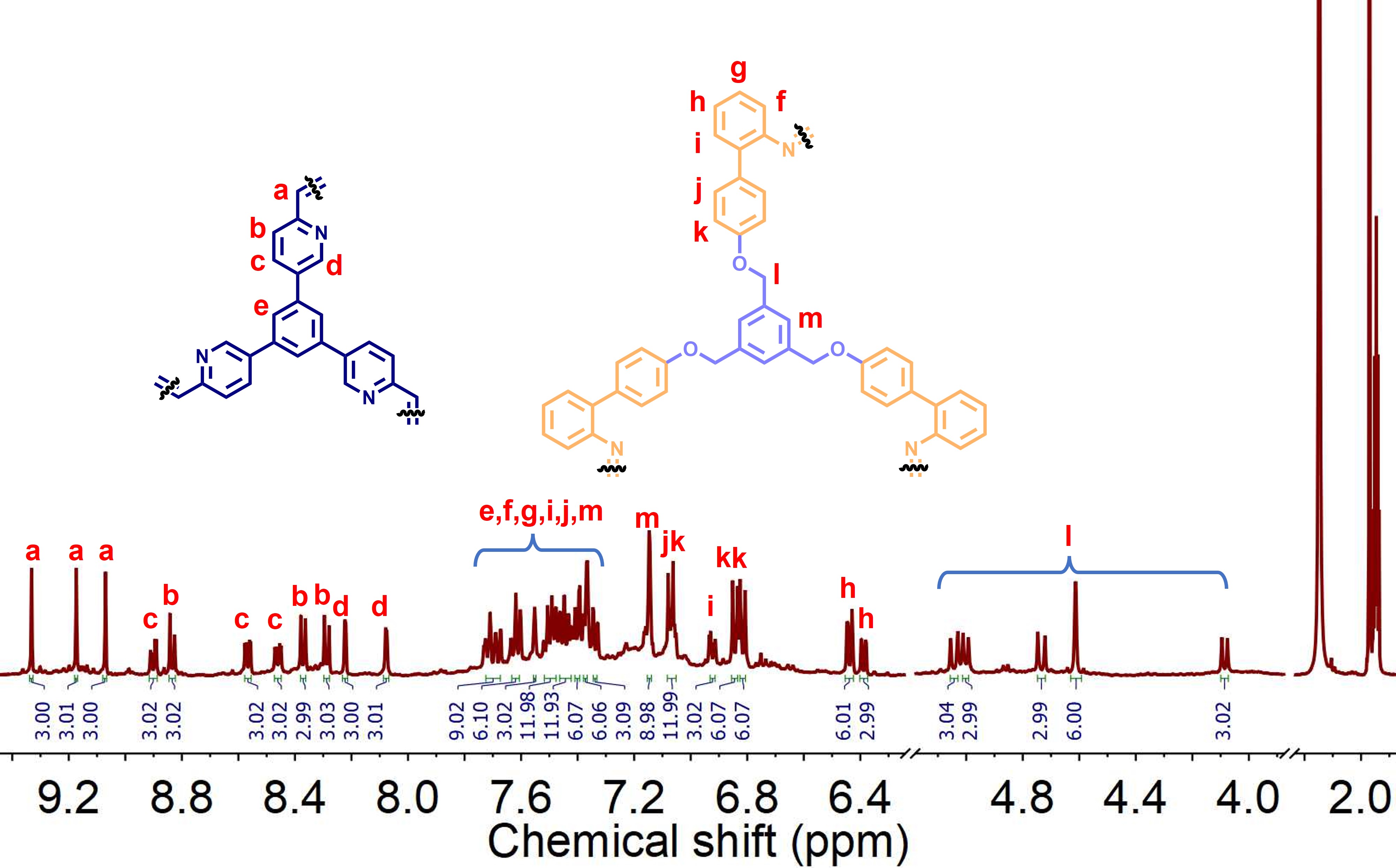


**Figure S6.** ^1^H NMR spectrum (500 MHz, 298 K, CD_3_CN) of **1**.


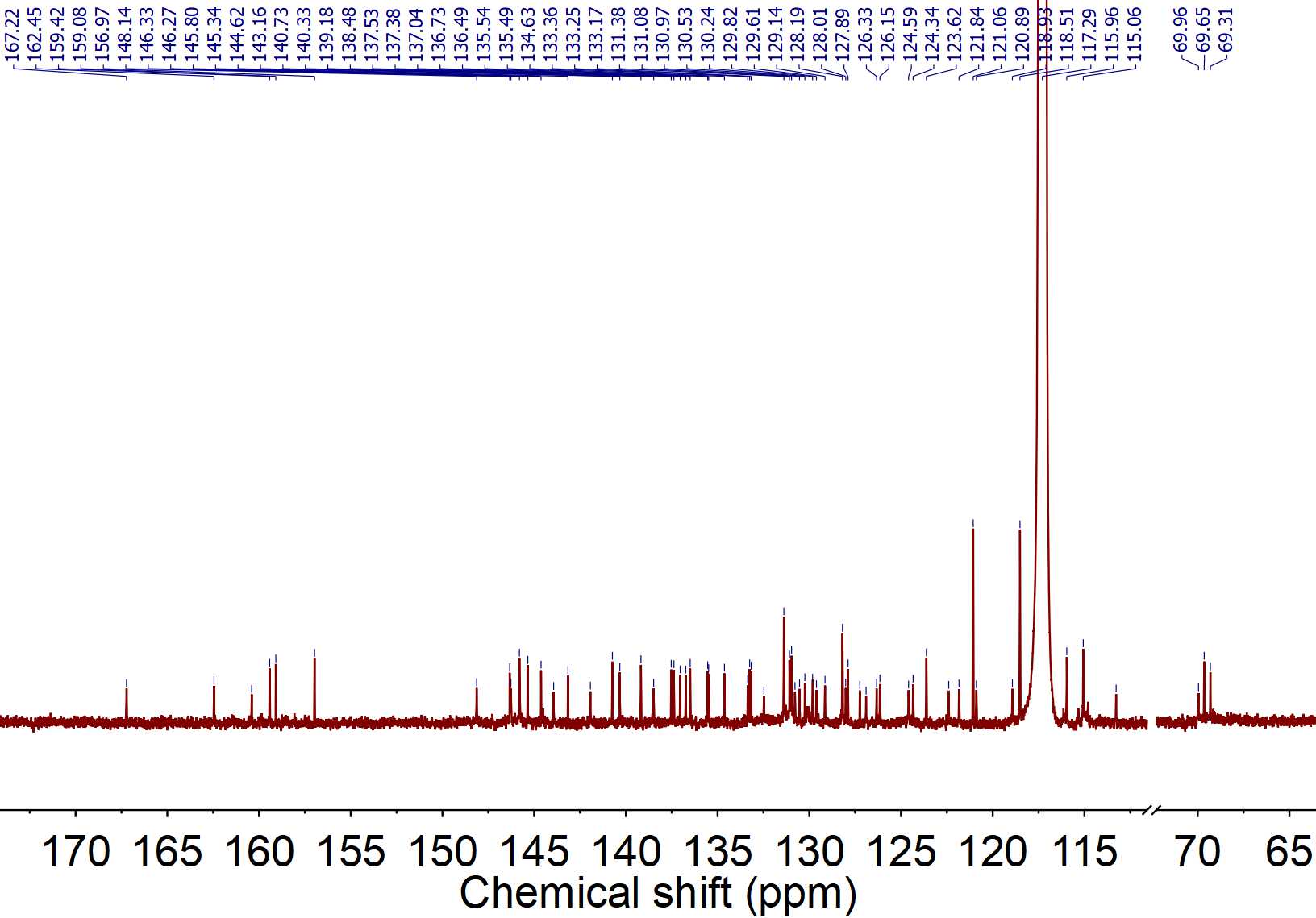


**Figure S7.** ^13^C NMR spectrum (126 MHz, 298 K, CD_3_CN) of **1**.


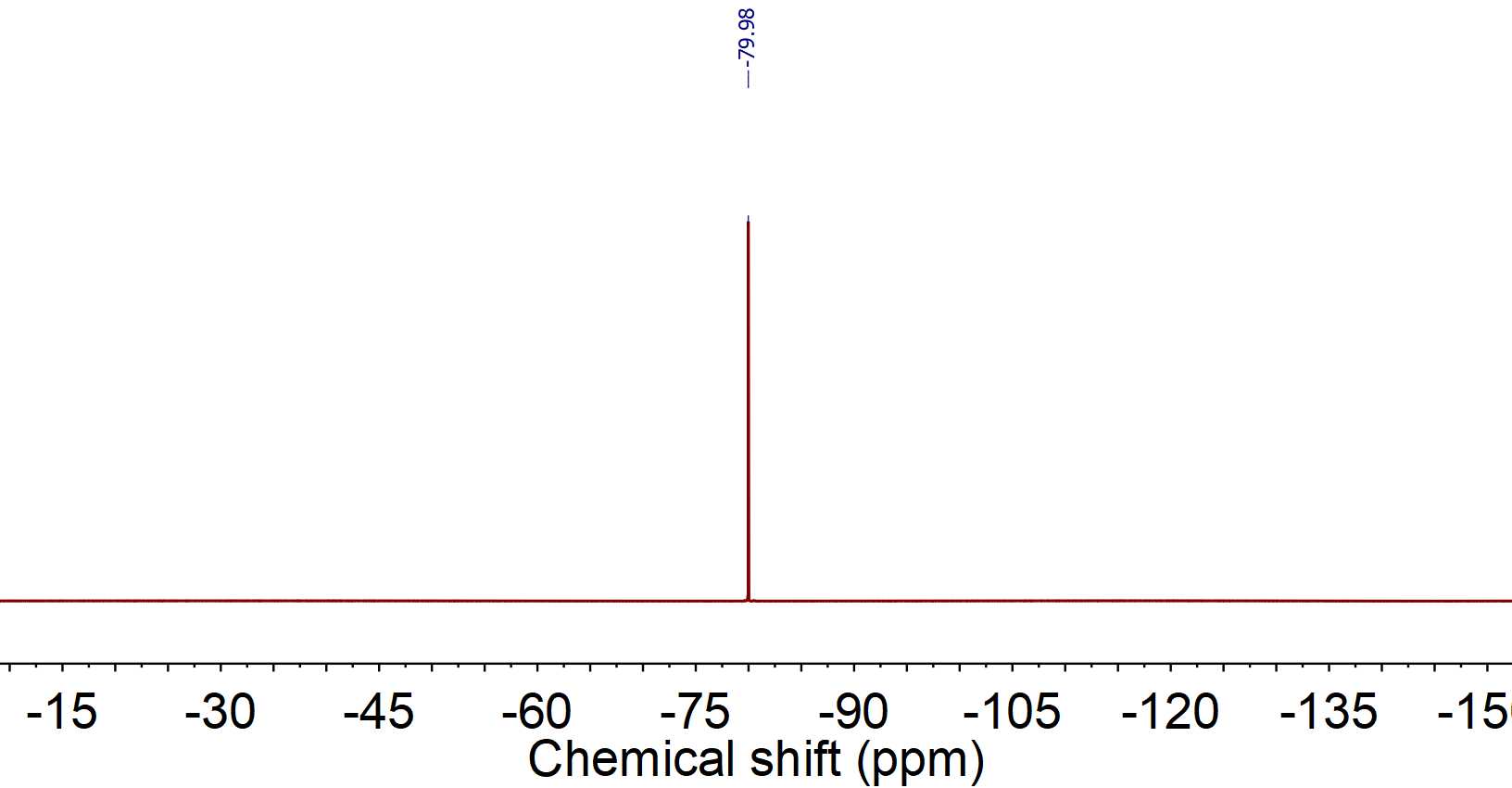


**Figure S8.** ^19^F NMR spectrum (376 MHz, 298 K, CD_3_CN) of **1.** δ –79.98 (s, CF_3_).


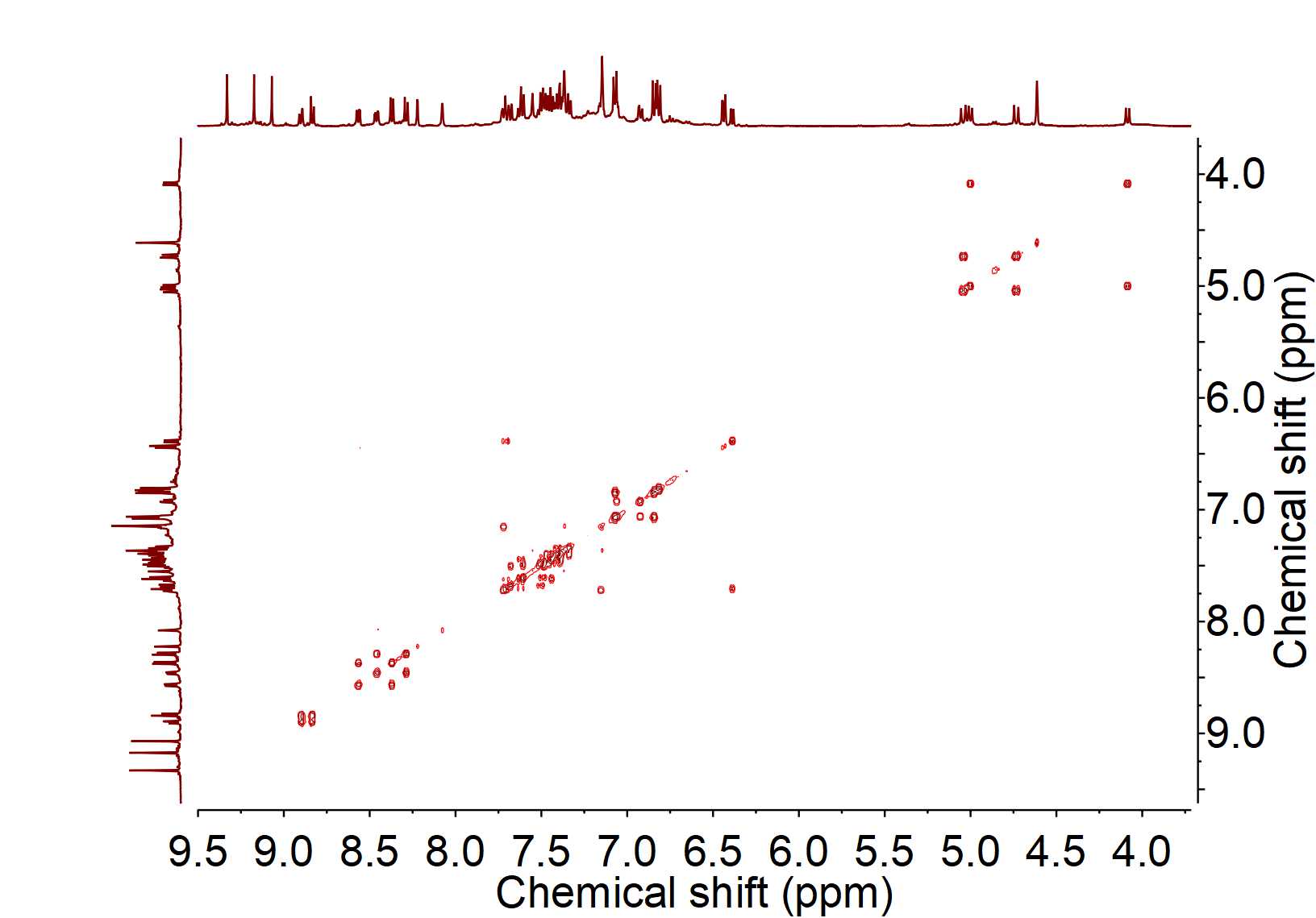


**Figure S9.** ^1^H–^1^H COSY spectrum (500 MHz, 298K, CD_3_CN) of **1**.


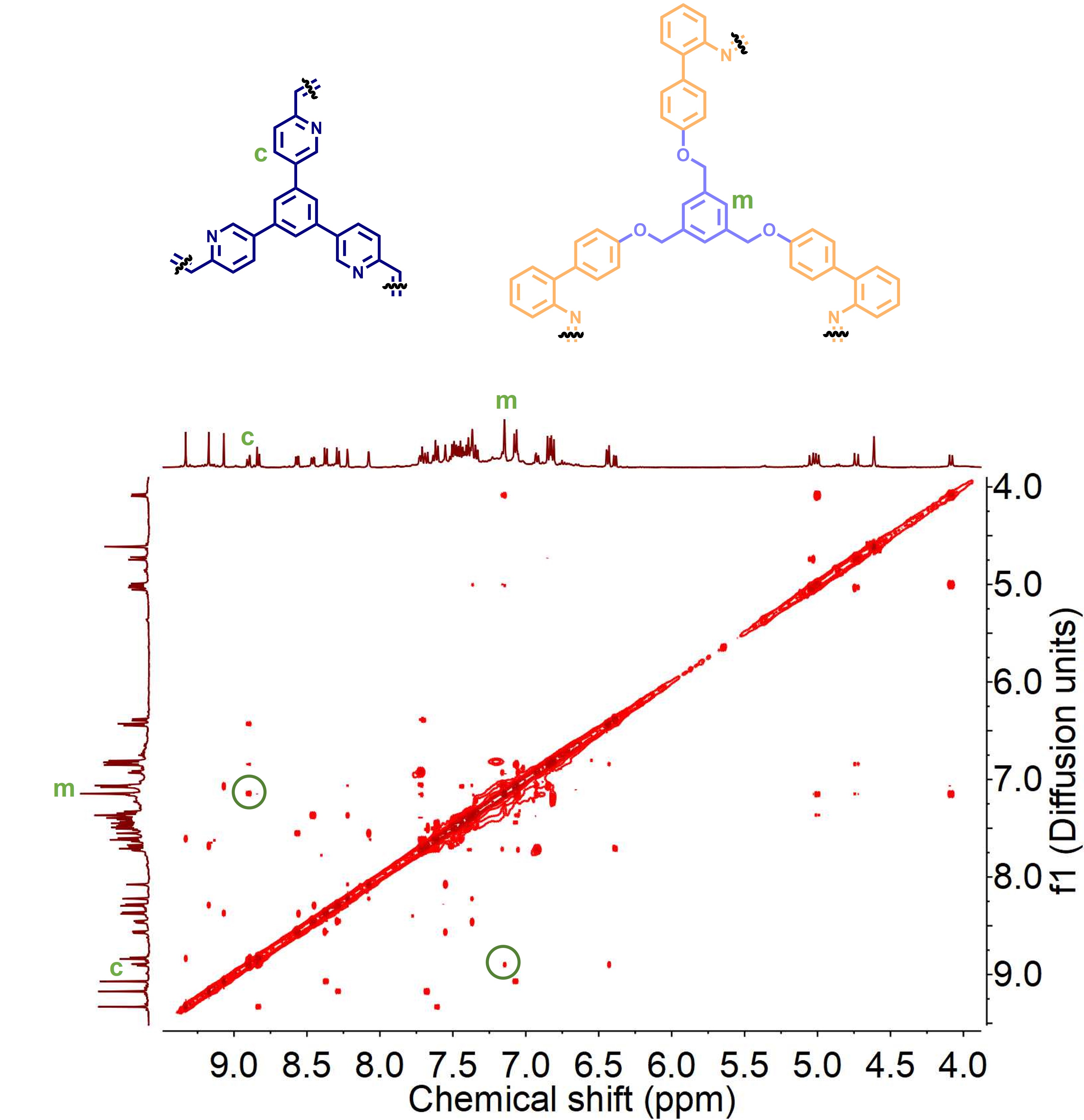


**Figure S10**. ^1^H–^1^H NOESY spectrum (500 MHz, 298K, CD_3_CN) of **1**. The correlation between protons H_c_ and H_m_ was indicated by a green circle.


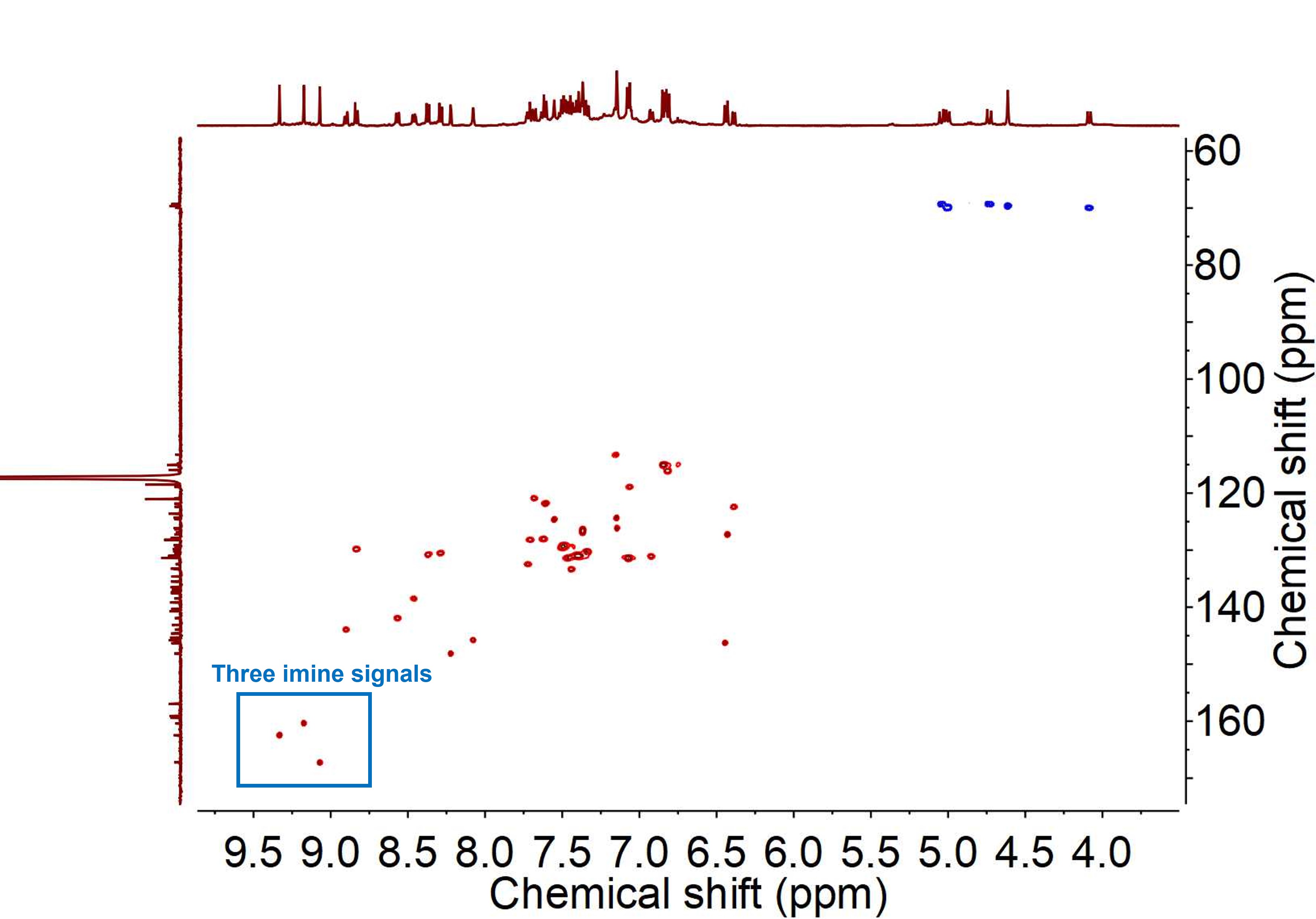


**Figure S11**. ^1^H–^13^C HSQC spectrum (500 MHz, 298K, CD_3_CN) of **1**.


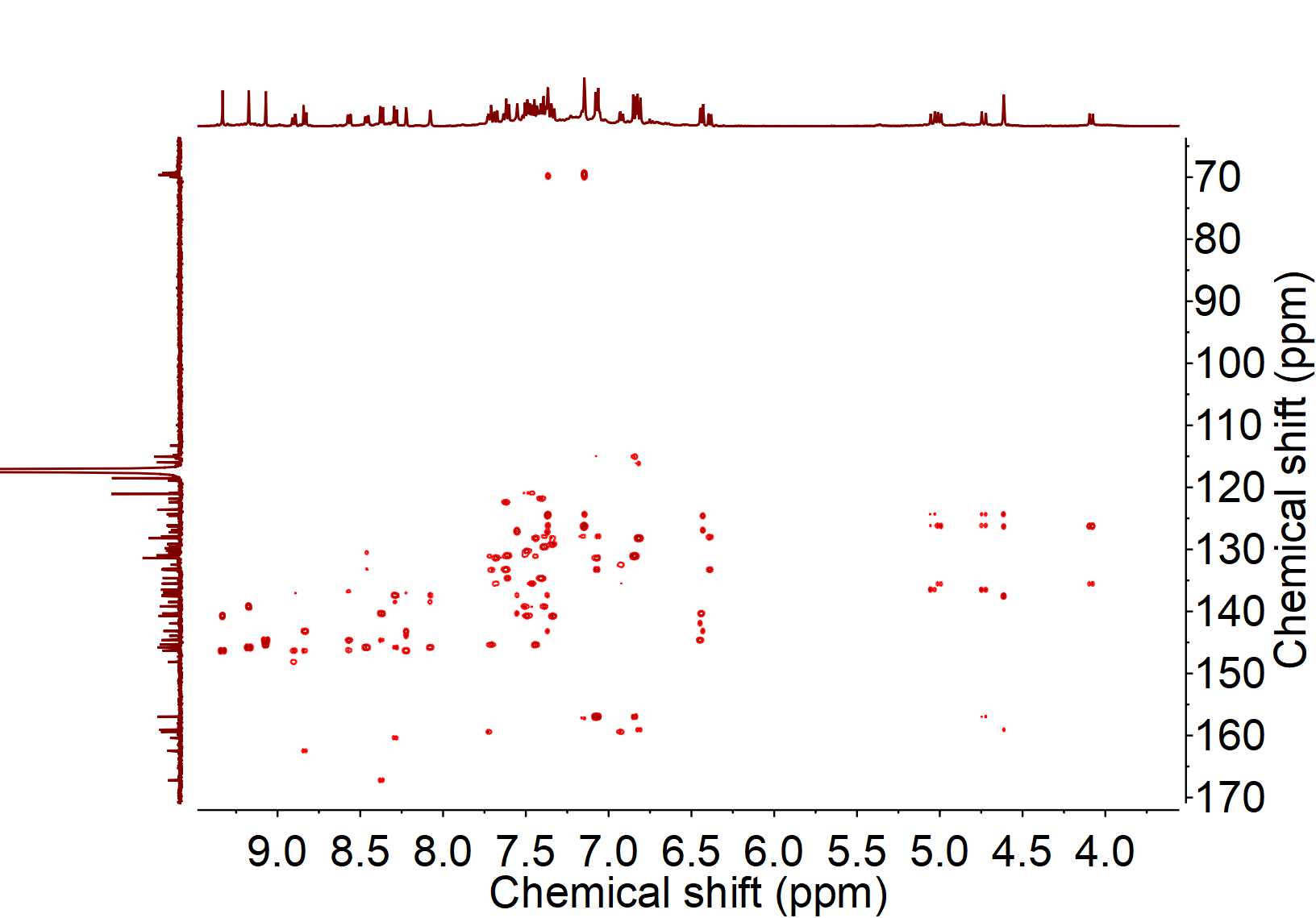


**Figure S12.** ^1^H–^13^C HMBC spectrum (500 MHz, 298K, CD_3_CN) of **1**.


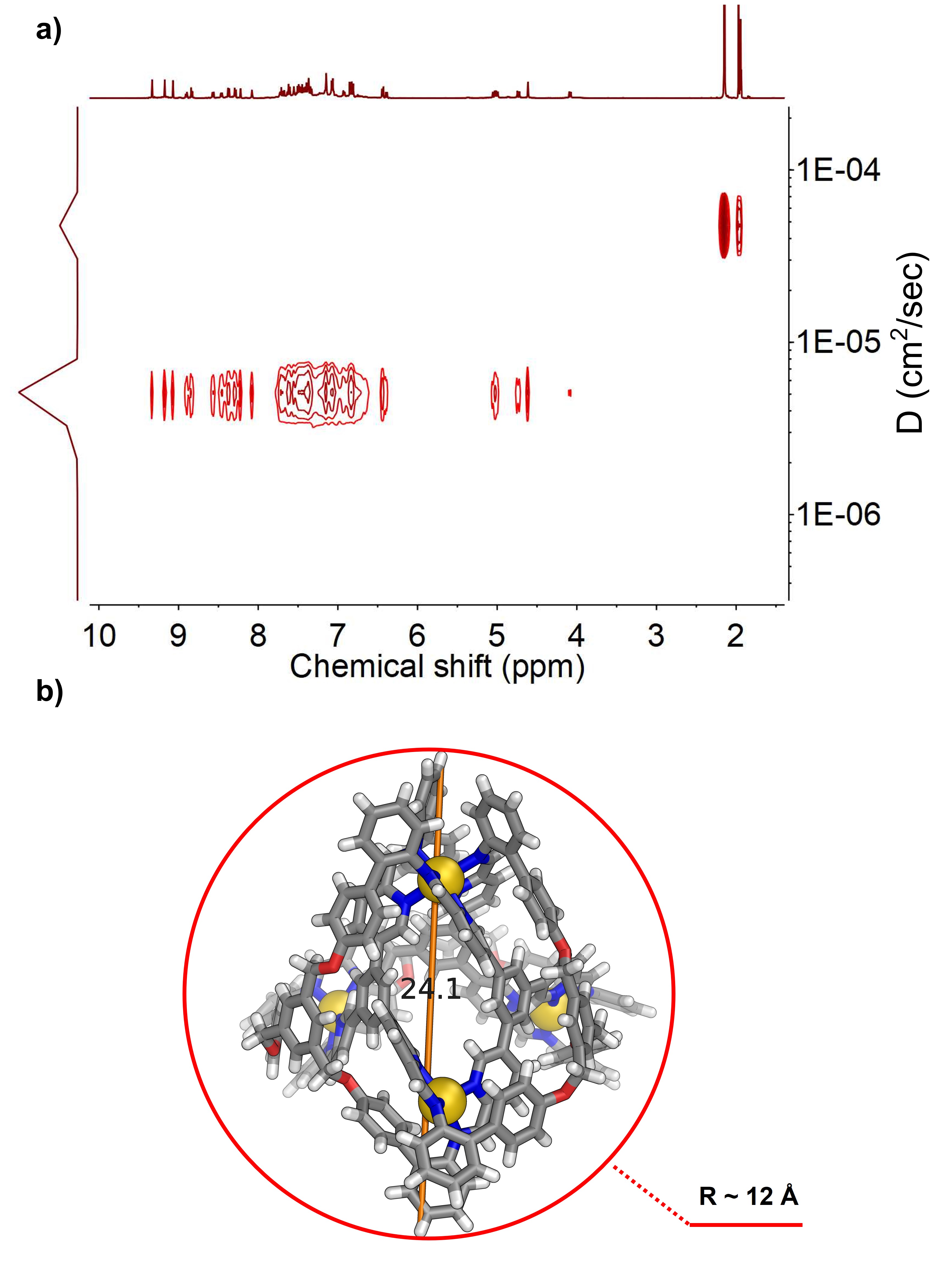


**Figure S13.** **a)** ^1^H DOSY spectrum (400 MHz, 298K, CD_3_CN) of **1**. Diffusion coefficient: D = 4.89 ×10^–10^ m^2^·s^–1^, with solvodynamic radius of 13 Å; **b)** The radius of the crystal structure was measured to be ~12 Å, consistent with the DOSY result obtained for **1**. The reference distance was derived from the obtained single-crystal structure, in which the maximum distance between two protons was measured and taken as the diameter of a spherical model for comparison. Minor differences may occur between the dimensions in the crystal structure and those in solution.


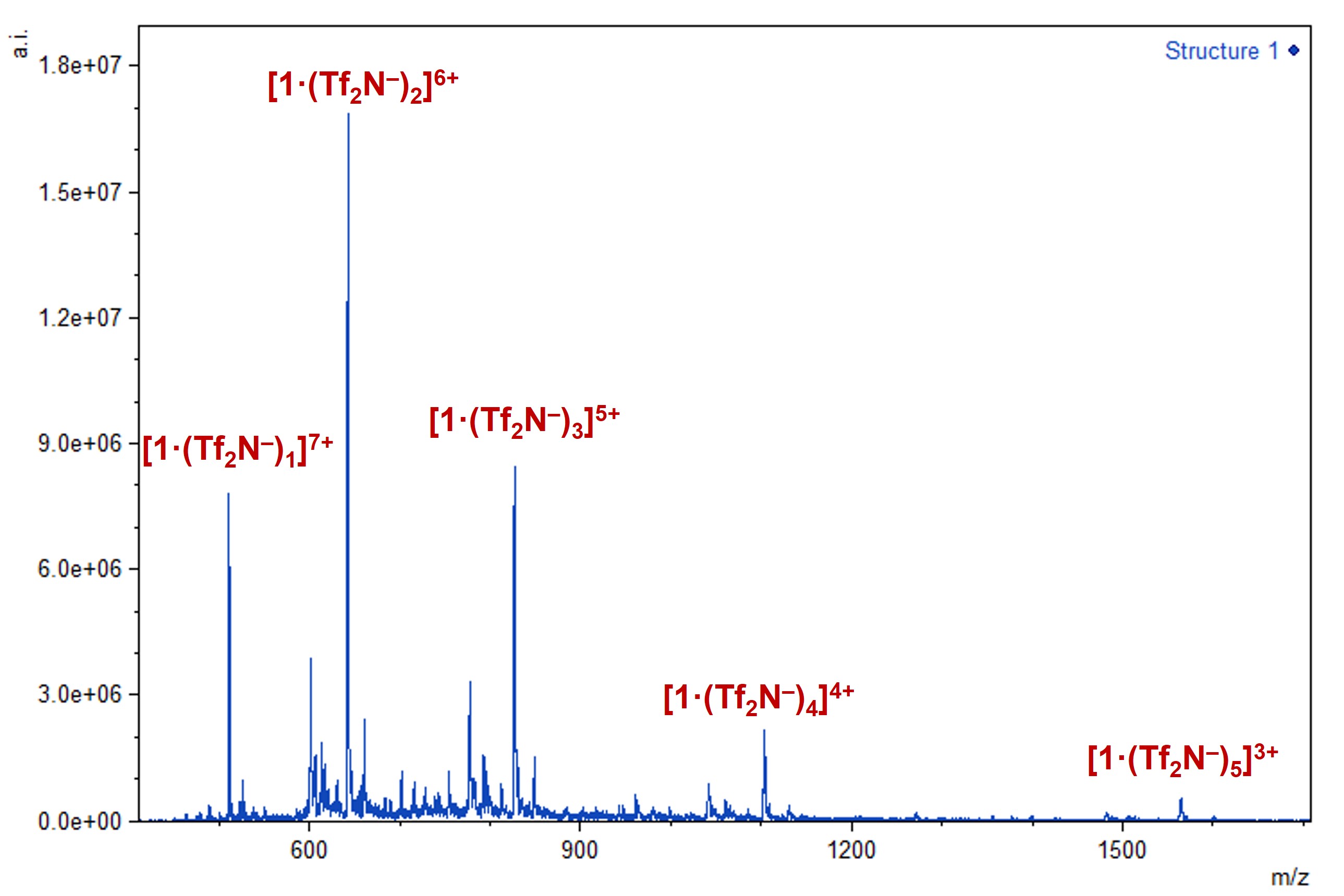


**Figure S14.** Low-resolution ESI-MS spectrum of **1**. *m*/*z* = 510.0 [**1**·(Tf_2_N^–^)_1_]^7+^,641.7 [**1**·(Tf_2_N^–^)_2_]^6+^,826.1 [**1**·(Tf_2_N^–^)_3_]^5+^,1102.7 [**1**·(Tf_2_N^–^)_4_]^4+^, 1563.9 [**1**·(Tf_2_N^–^)_5_]^3+^. Calculated peaks: m/z = 509.8 [**1**·(Tf_2_N^–^)_1_]^7+^,641.6 [**1**·(Tf_2_N^–^)_2_]^6+^,825.9 [**1**·(Tf_2_N^–^)_3_]^5+^,1102.4 [**1**·(Tf_2_N^–^)_4_]^4+^, 1563.3 [**1**·(Tf_2_N^–^)_5_]^3+^.


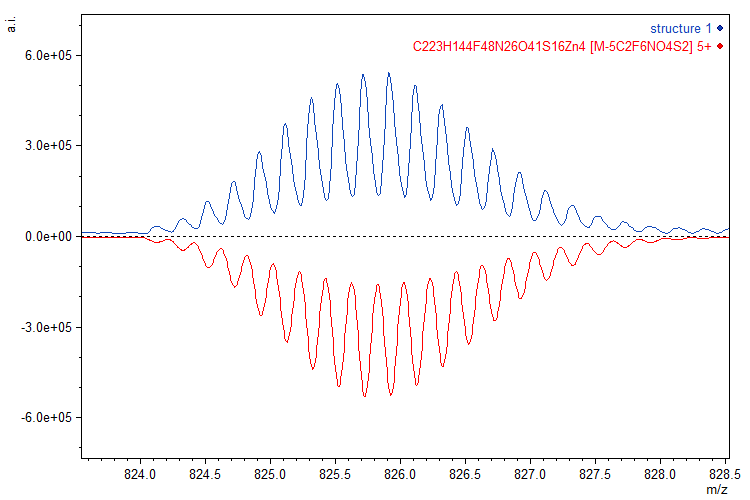


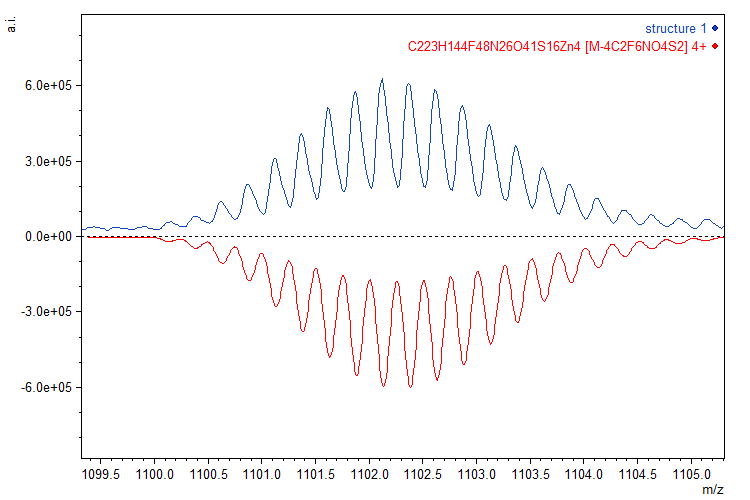


**Figure S15.** High-resolution ESI-mass spectrometry analysis of **1** showing the observed (top blue) and theoretical (bottom red) isotope patterns for the 5+ and 4+ peaks.

3.2 Self-assembly of **2**, **3** and **4**

3.2.1 Self-assembly of **2** and **3**

**2** and **3** with TfO^–^ counterions were constructed following previous literature.^3^ All spectroscopic data matched those previously reported.

3.2.2 Self-assembly of **4**


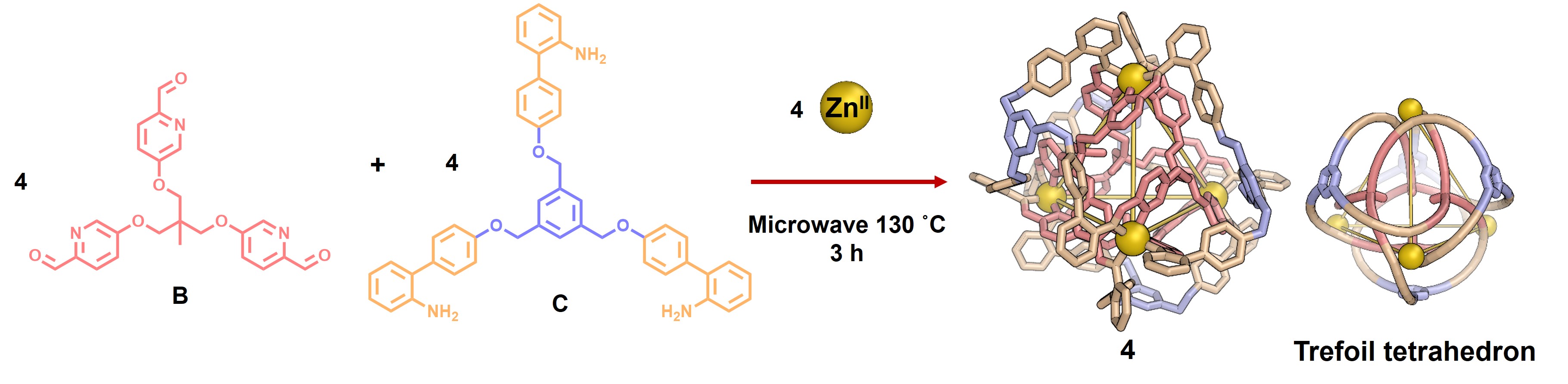


**Scheme S4.** Construction of **4**

Subcomponent **B** (5.0 mg, 11.5 µmol, 4 equiv.) reacted with subcomponent **C** (7.7 mg, 11.5 µmol, 4 equiv.) and zinc(II) bis(trifluoromethanesulfonimide) (7.2 mg, 11.5 µmol, 4 equiv.) in acetonitrile (2 mL) to produce **4**. The reaction mixture was heated and stirred under microwave irradiation for 3 h at 130 ˚C. Then the light-yellow solution was concentrated to 0.5 mL and diethyl ether (14 mL) was added. The precipitate was collected by centrifugation and washed with excess diethyl ether to give **4** in 75 % yield.

**^1^H NMR (500 MHz, CD_3_CN)** δ 8.71 (s, 12H), 8.10 (d, *J* = 8.5 Hz, 12H), 7.94 (s, 12H), 7.60 – 7.54 (m, 24H), 7.47 (dd, *J* = 8.8, 2.7 Hz, 12H), 7.22 (ddd, *J* = 8.0, 7.2, 1.7 Hz, 12H), 7.15 – 6.99 (br, 24H), 6.61 – 6.44 (br, 24H), 5.97 (d, *J* = 2.7 Hz, 12H), 5.14 (d, *J* = 11.6 Hz, 12H), 5.06 (d, *J* = 11.5 Hz, 12H), 4.96 (dd, *J* = 7.9, 1.2 Hz, 12H), 3.73 (d, *J* = 9.0 Hz, 12H), 2.87 (d, *J* = 9.1 Hz, 12H), 0.47 (s, 12H).

**^13^C NMR (126 MHz, CD_3_CN)** δ 167.0, 158.4, 157.3, 146.3, 138.7, 138.7, 138.5, 132.8, 131.5, 131.3, 130.5, 130.1, 129.4, 128.6, 128.0, 126.4, 122.4, 121.7, 119.9 (q, ^1^*J*_CF_ = 318.8 Hz, Tf_2_N^–^), 114.5, 70.7, 68.7, 38.4, 12.5.

**ESI-MS**: *m*/*z*: 558.2 [**4**]^8+^, 678.0 [**4**·(Tf_2_N^–^)_1_]^7+^,837.7 [**4**·(Tf_2_N^–^)_2_]^6+^,1061.2 [**4**·(Tf_2_N^–^)_3_]^5+^,1396.5 [**4**·(Tf_2_N^–^)_4_]^4+^.


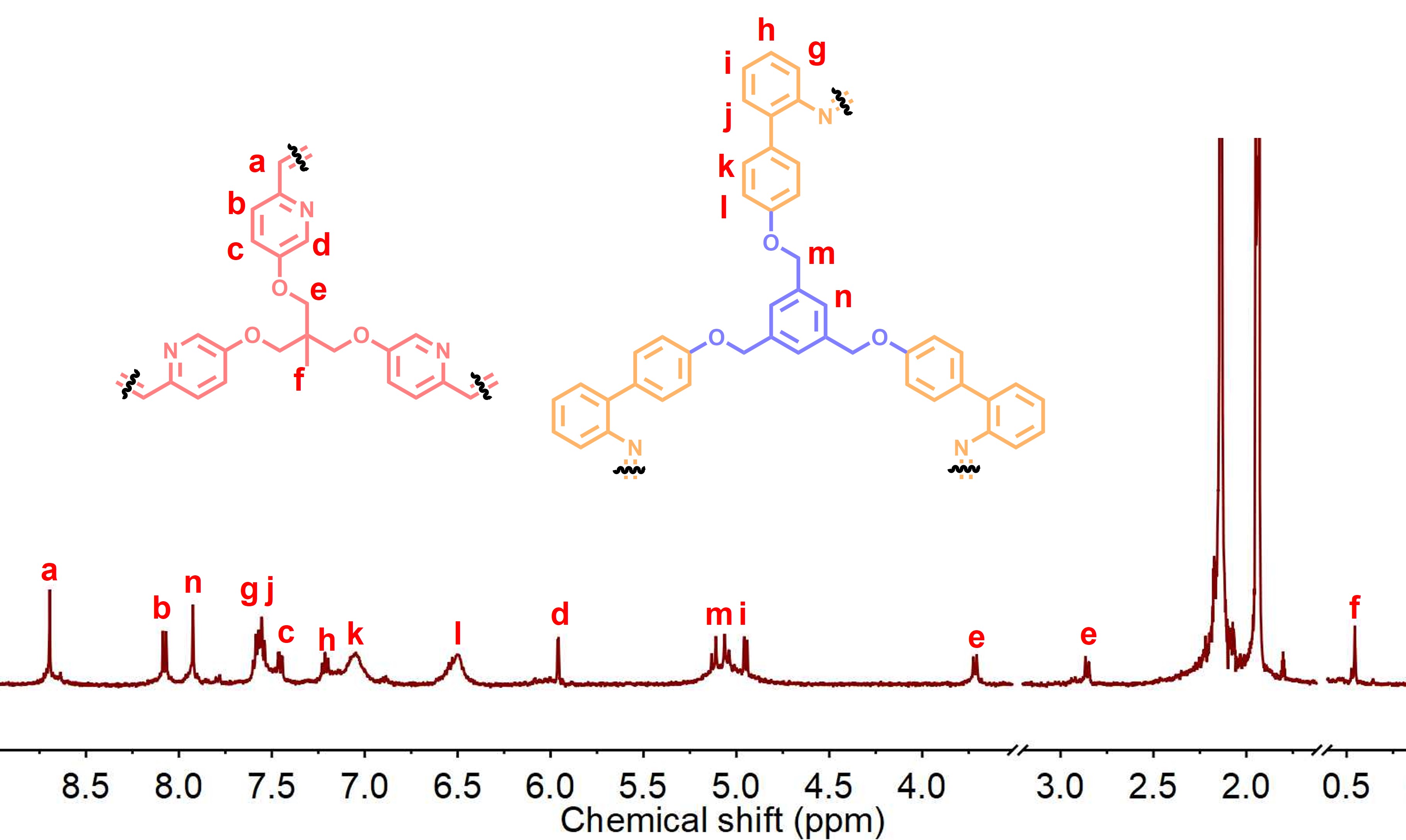


**Figure S16**. ^1^H NMR spectrum (500 MHz, 298 K, CD_3_CN) of **4**.


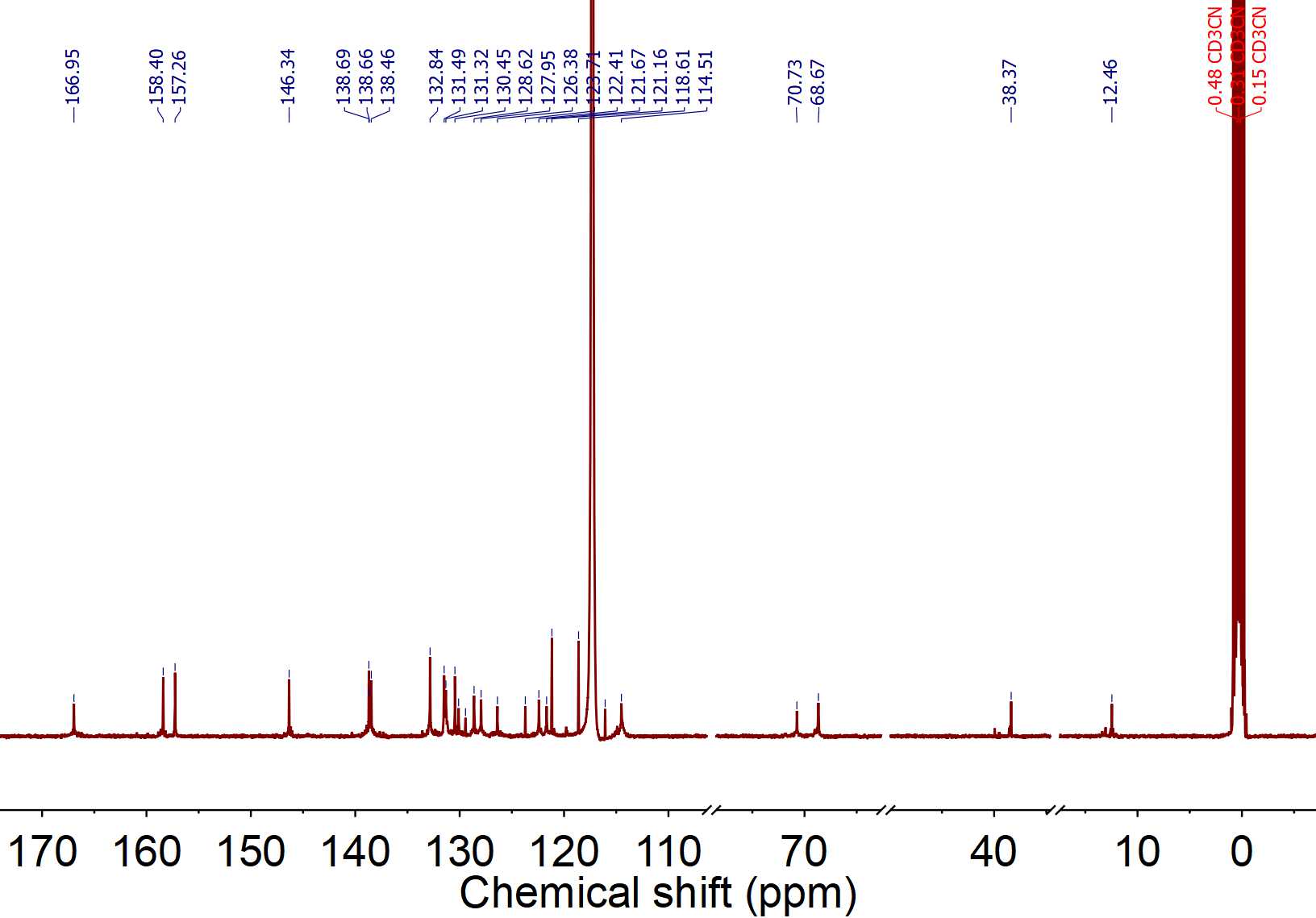


**Figure S17**. ^13^C NMR spectrum (500 MHz, 298 K, CD_3_CN) of **4**.


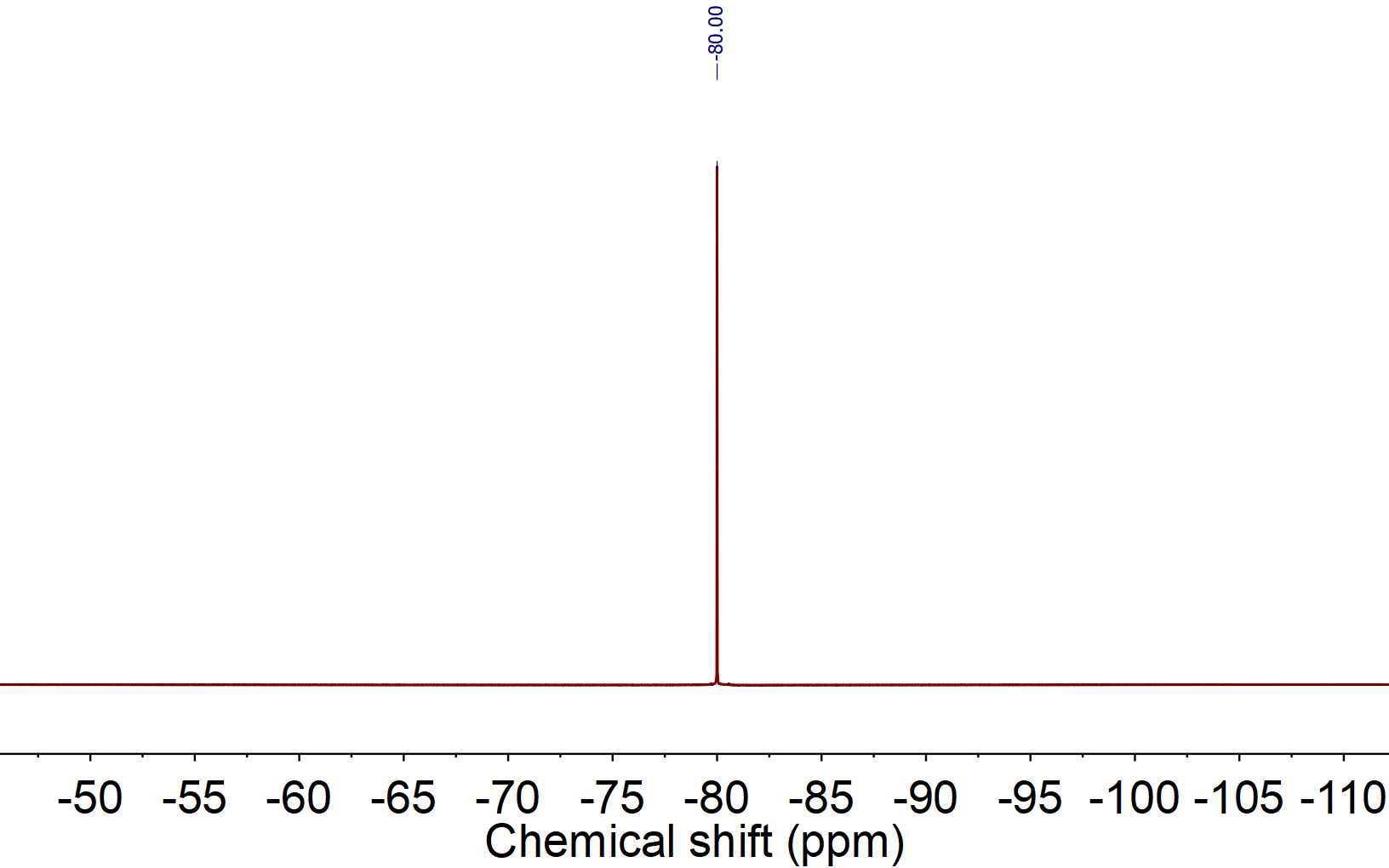


**Figure S18.** ^19^F NMR spectrum (376 MHz, 298 K, CD_3_CN) of **4**. δ –80.00 (s, CF_3_).


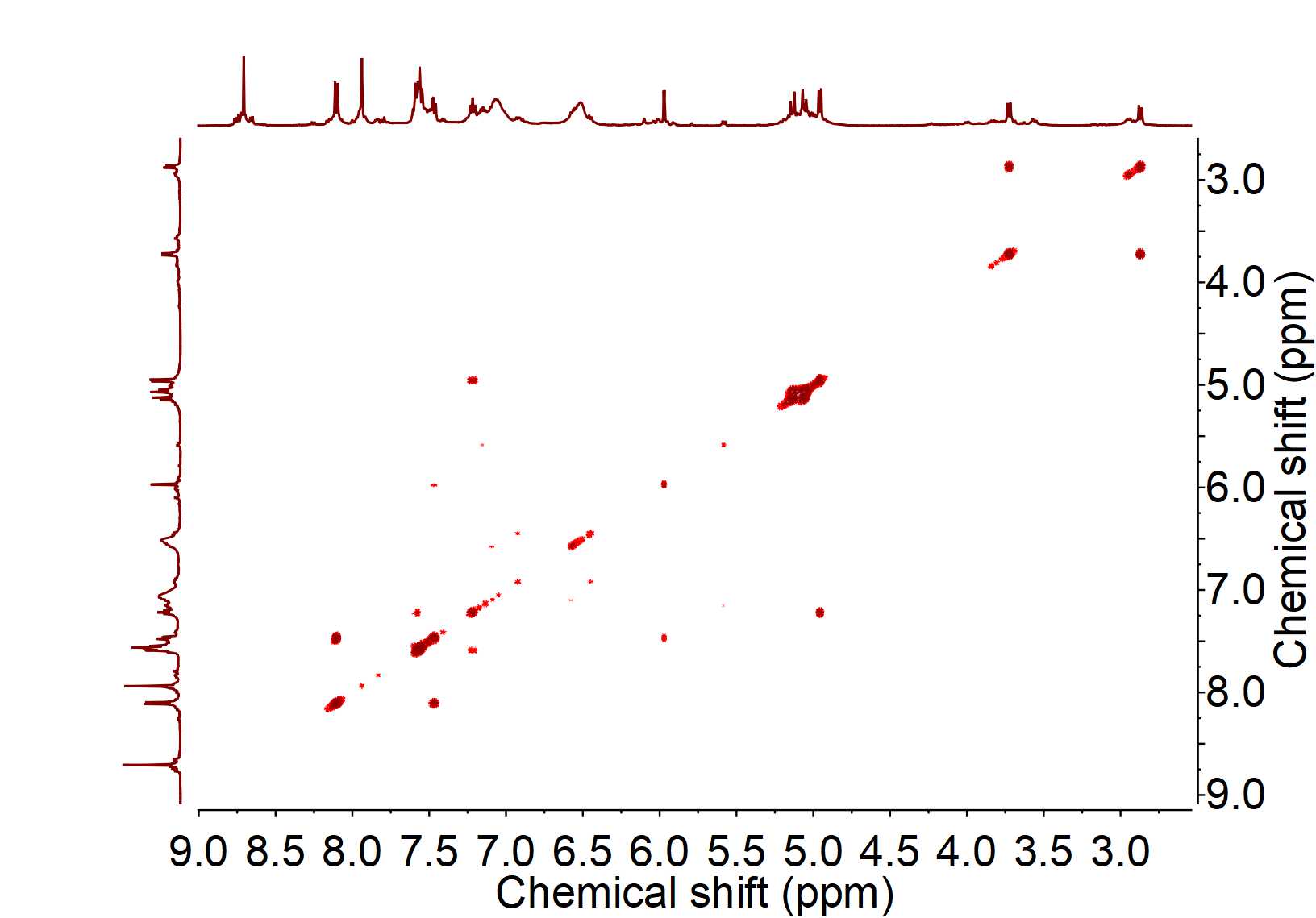


**Figure S19.** ^1^H–^1^H COSY spectrum (500 MHz, 298 K, CD_3_CN) of **4**.


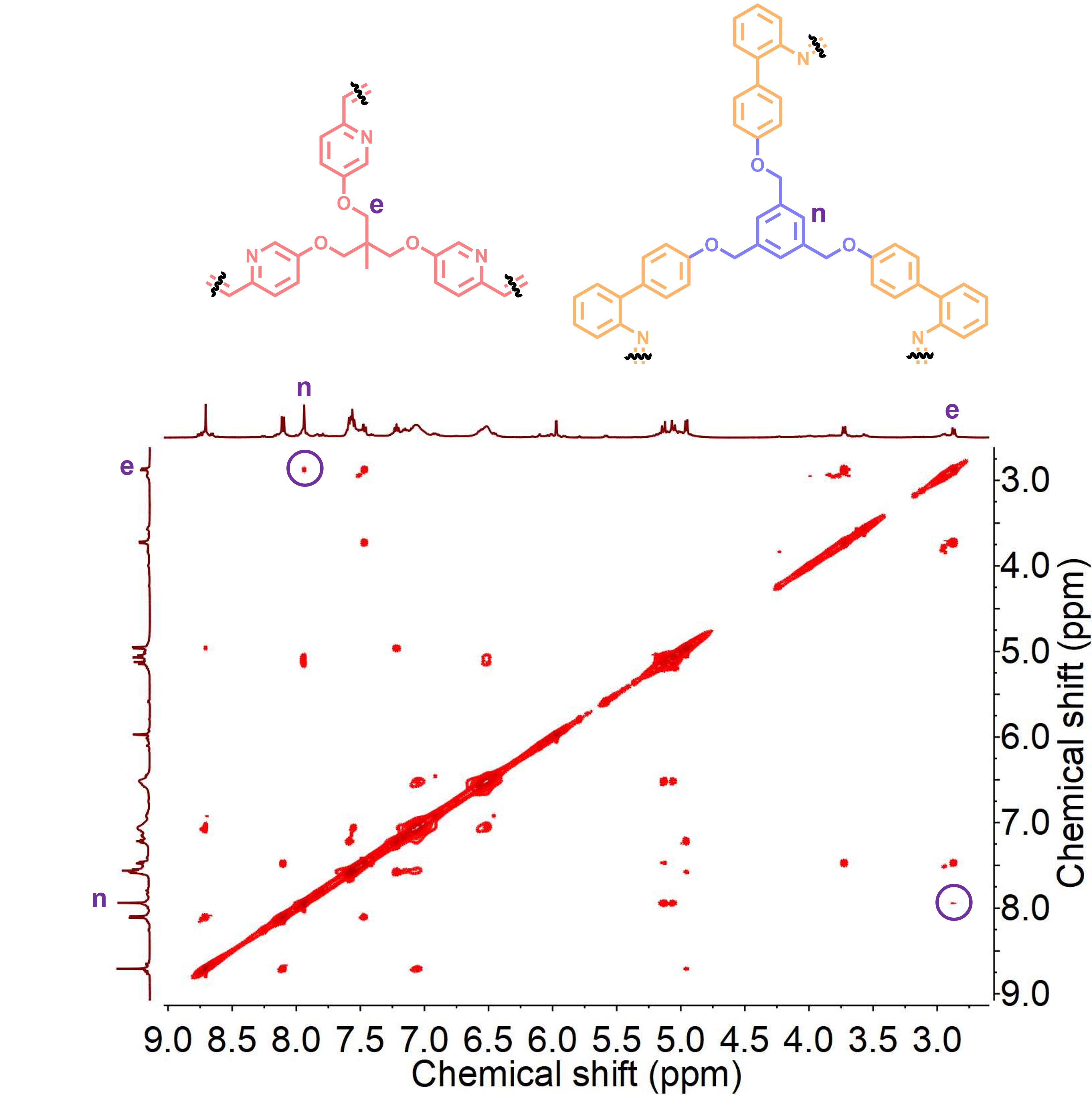


**Figure S20.** Partial ^1^H–^1^H NOESY spectrum (500 MHz, 298 K, CD_3_CN) of **4**.


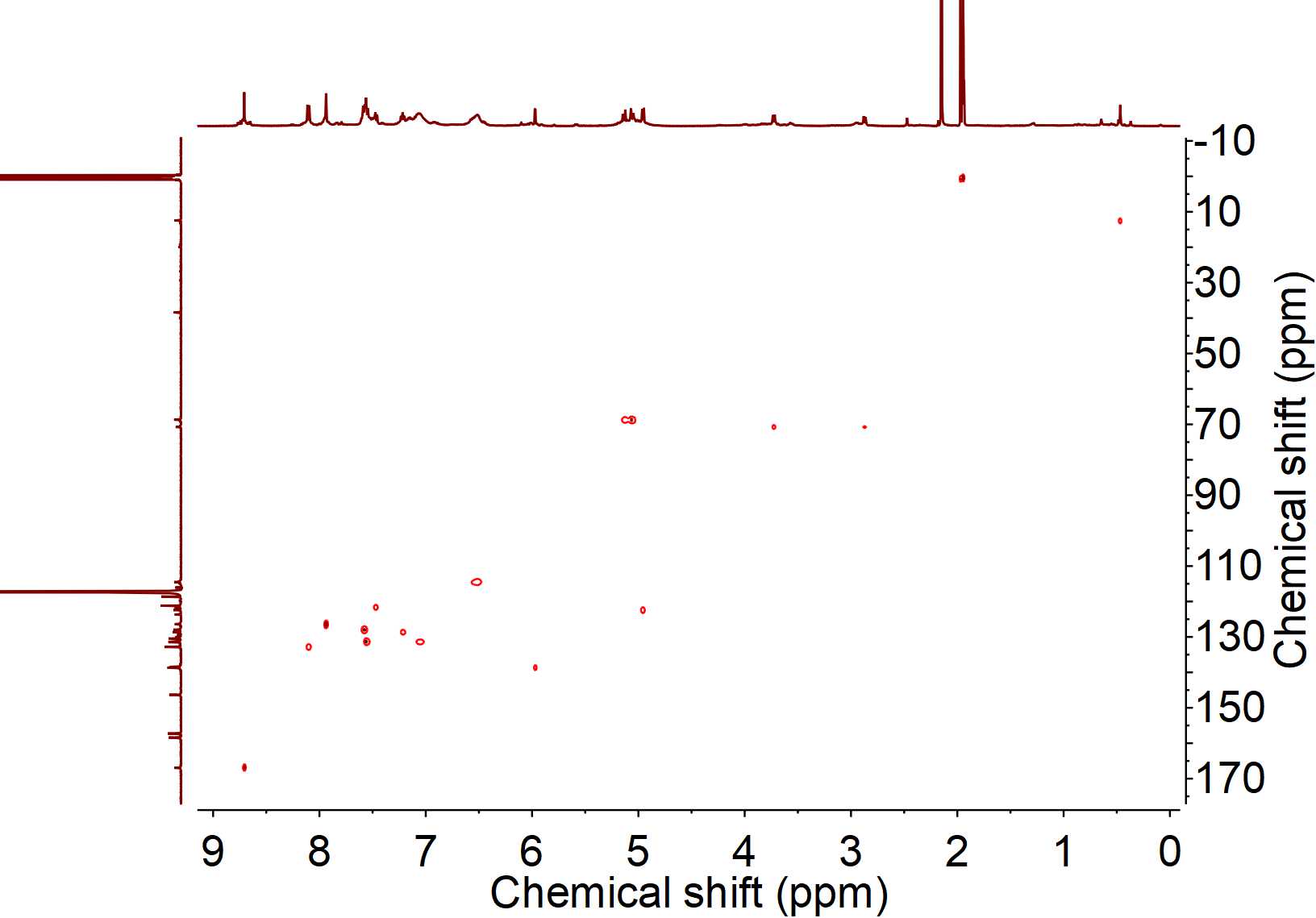


**Figure S21.** ^1^H–^13^C HSQC spectrum (500 MHz, 298 K, CD_3_CN) of **4**.


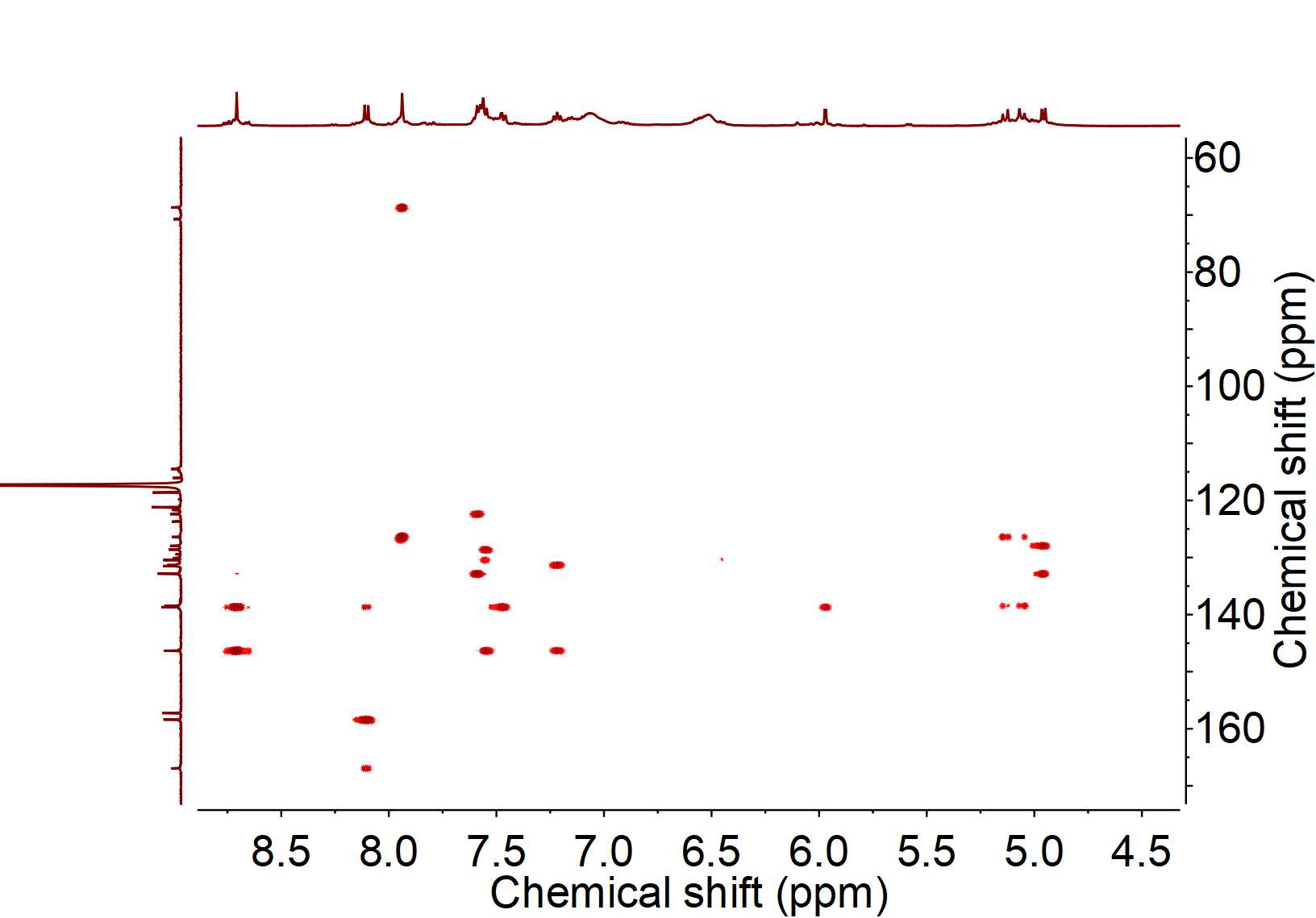


**Figure S22.** Partial ^1^H–^13^C HMBC spectrum (500 MHz, 298 K, CD_3_CN) of **4**.


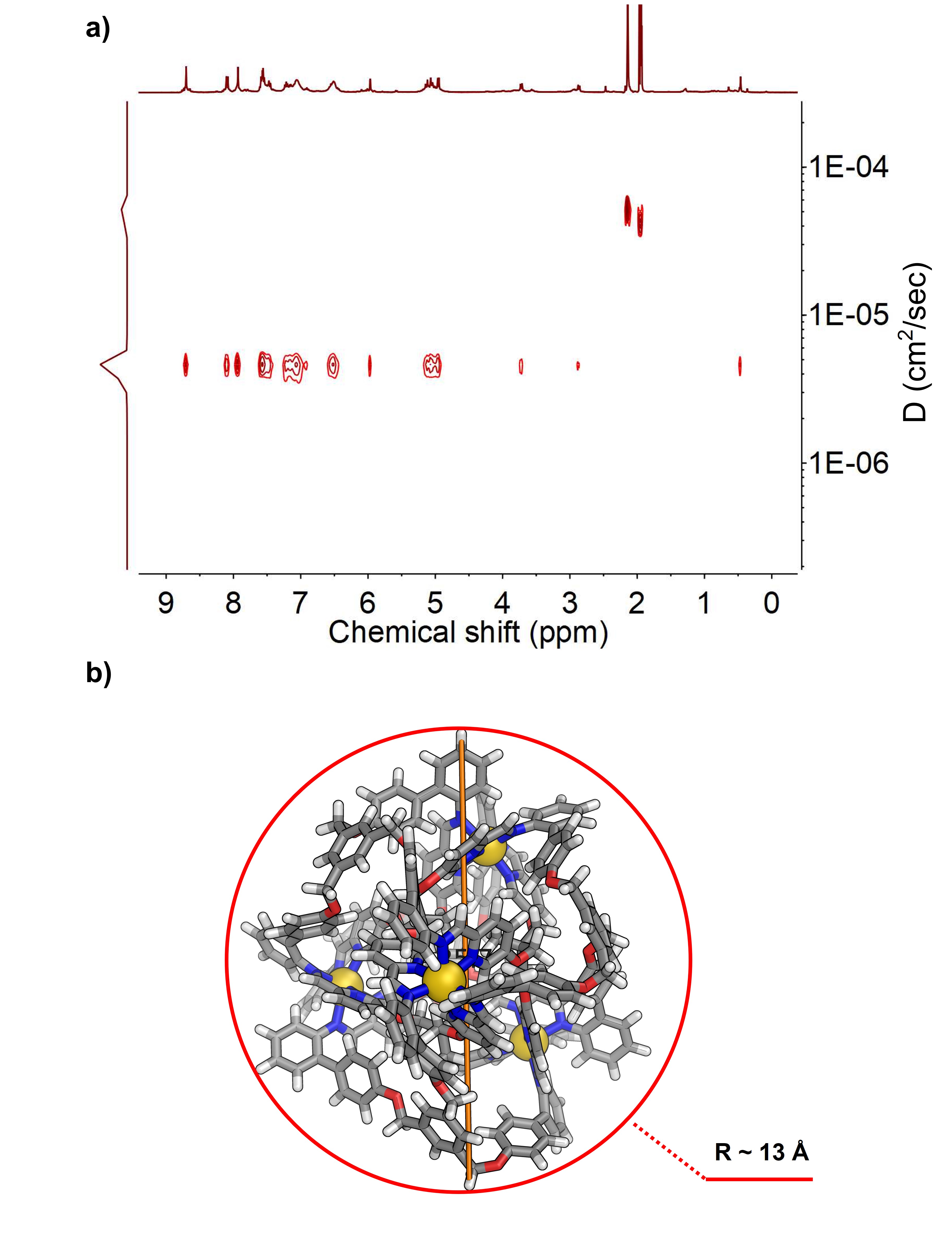


**Figure S23.** **a)** ^1^H DOSY spectrum (400 MHz, 298 K, CD_3_CN) of **4**. Diffusion coefficient: D = 4.63 × 10^–10^ m^2^ s^–1^, with solvodynamic radius of 14 Å; **b)** The radius of the modeled structure was measured to be ~13 Å, consistent with the DOSY result obtained for **4**. The reference distance was derived from the structure model, in which the maximum distance between two protons was measured and taken as the diameter of a spherical model for comparison.


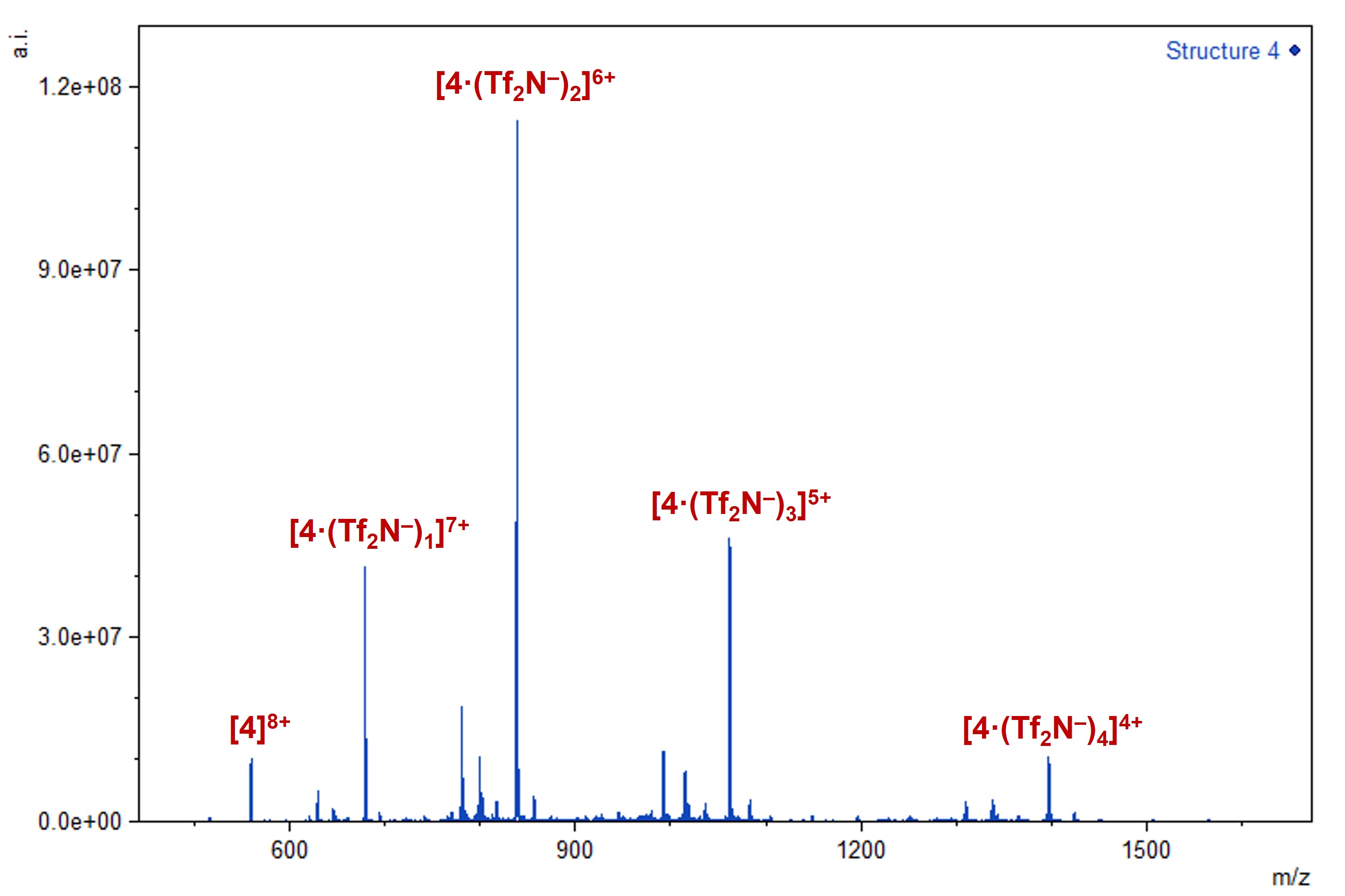


**Figure S24.** Low-resolution ESI-MS spectrum of **4**. *m*/*z* = 558.2 [**4**]^8+^, 678.0 [**4**·(Tf_2_N^–^)_1_]^7+^,837.7 [**4**·(Tf_2_N^–^)_2_]^6+^,1061.2 [**4**·(Tf_2_N^–^)_3_]^5+^,1396.5 [**4**·(Tf_2_N^–^)_4_]^4+^. Calculated peaks: *m*/*z* = 558.3 [**4**]^8+^, 678.1 [**4**·(Tf_2_N^–^)_1_]^7+^,837.8 [**4**·(Tf_2_N^–^)_2_]^6+^,1061.4 [**4**·(Tf_2_N^–^)_3_]^5+^,1396.7 [**4**·(Tf_2_N^–^)_4_]^4+^.


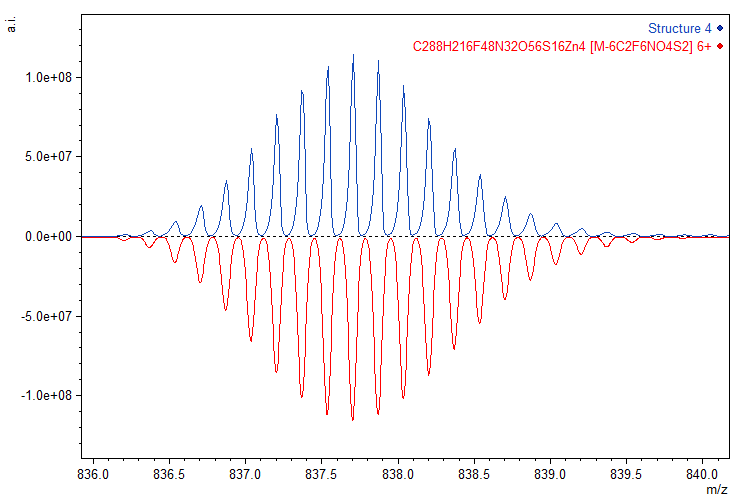


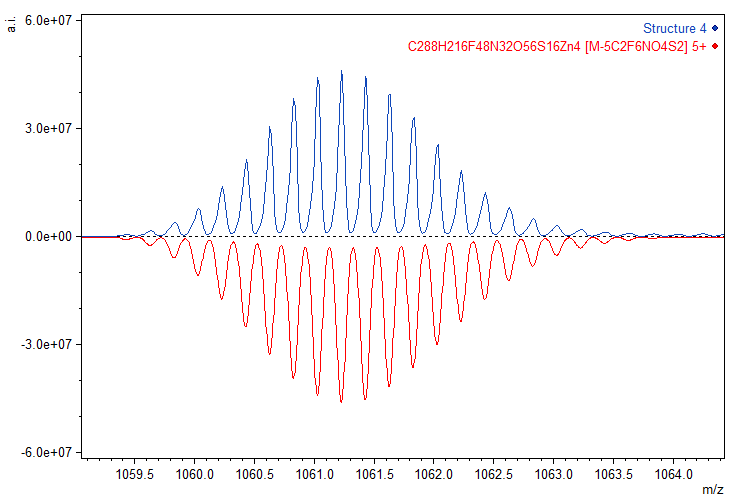


**Figure S25.** High-resolution ESI-mass spectrometry analysis of **4** showing the observed (top blue) and theoretical (bottom red) isotope patterns for the 6+ and 5+ peaks.

3.3 Self-assembly of tetrahedral frameworks **5**, **6** and TfO^–^⊂**7**

3.3.1 Self-assembly of **5** and TfO^–^⊂**7**

**5** ^9^ and TfO^–^⊂**7** ^2^ were constructed following previous literature. All spectroscopic data matched those previously reported.

3.3.2 Self-assembly of **6**


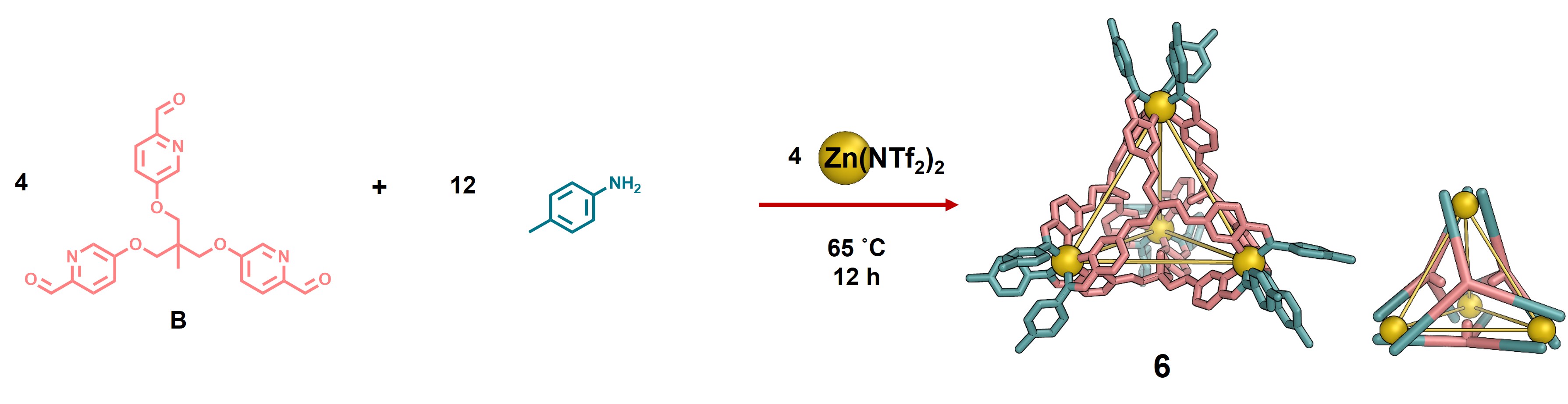


**Scheme S5.** Construction of **6**

Subcomponent **B** (5.0 mg, 11.5 µmol, 4 equiv.) reacted with *p*-toluidine (4.1 mg, 37.9 µmol, 13.2 equiv.) and zinc(II) bis(trifluoromethanesulfonimide) (7.2 mg, 11.5 µmol, 4 equiv.) in acetonitrile (2 mL) to produce **6**. The reaction mixture was heated and stirred under heating for 12 h at 65 ˚C. Then the mixture was concentrated to 0.2 mL and diethyl ether (15 mL) was added. The precipitate was collected by centrifugation and washed with excess diethyl ether to give **6** in 95 % yield.

**^1^H NMR (500 MHz, CD_3_CN) for 6** δ 8.52 (s, 12H), 8.22 (d, *J* = 8.7 Hz, 12H), 7.82 (dd, *J* = 8.8, 2.8 Hz, 12H), 7.27 (d, *J* = 2.7 Hz, 12H), 7.08 (d, *J* = 8.5 Hz, 24H), 6.28 (d, *J* = 8.4 Hz, 24H), 4.32 (t, *J* = 6.7 Hz, 24H), 2.34 (s, 36H), 1.18 (s, 12H).

**^13^C NMR (126 MHz, CD_3_CN) for 6** δ 162.9, 160.3, 144.9, 139.4, 139.3, 138.4, 132.5, 129.9, 123.0, 121.7, 119.9 (q, ^1^*J*_CF_ = 214.2 Hz, Tf_2_N^–^), 72.2, 40.3, 20.0, 13.0.

**ESI-MS**: *m*/*z*: 605.5 [**6**·(Tf_2_N^–^)_2_]^6+^,782.6 [**6**·(Tf_2_N^–^)_3_]^5+^,1048.2 [**6**·(Tf_2_N^–^)_4_]^4+^, 1490.9 [**6**·(Tf_2_N^–^)_5_]^3+^.


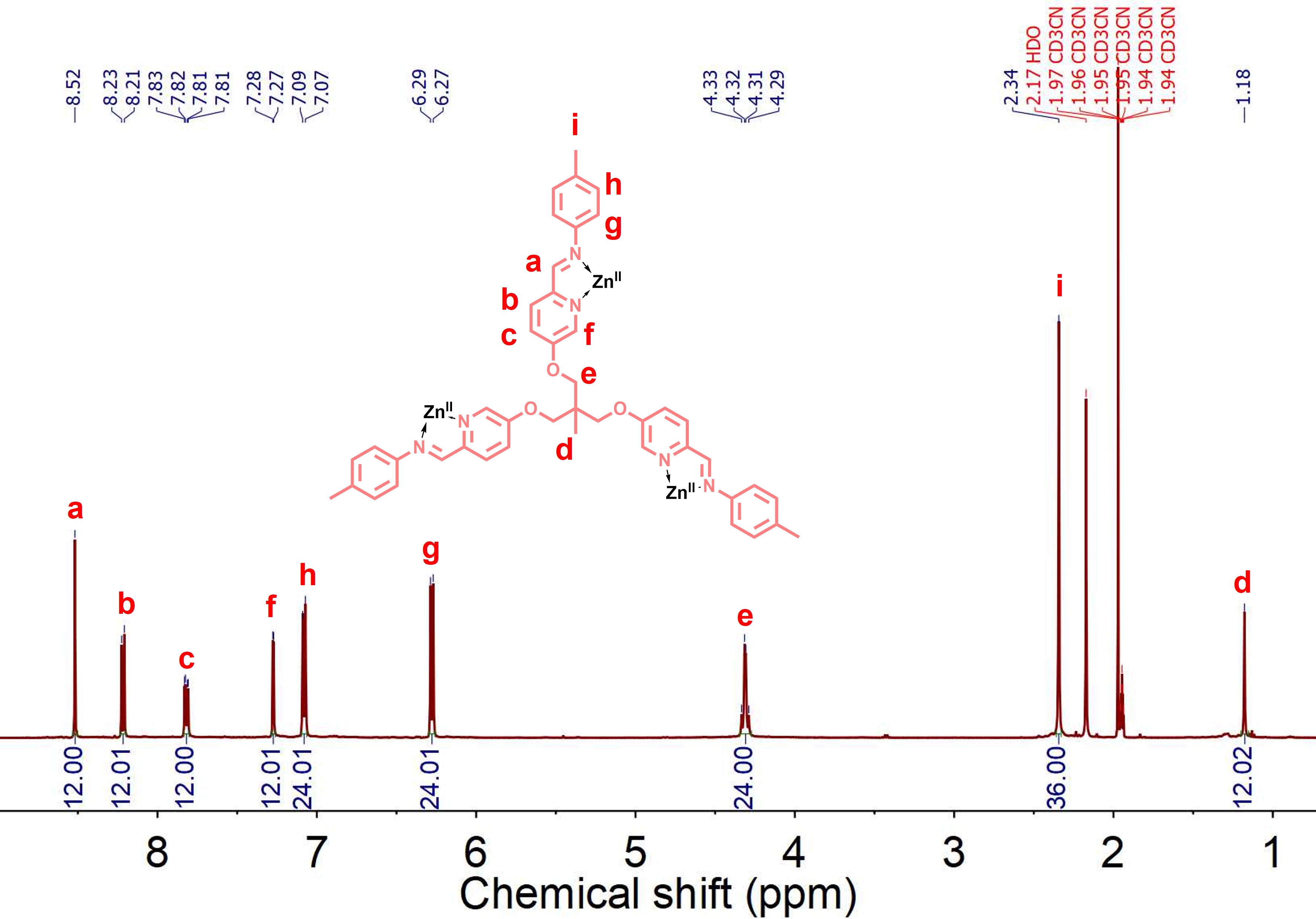


**Figure S26**. ^1^H NMR spectrum (500 MHz, 298 K, CD_3_CN) of **6**.


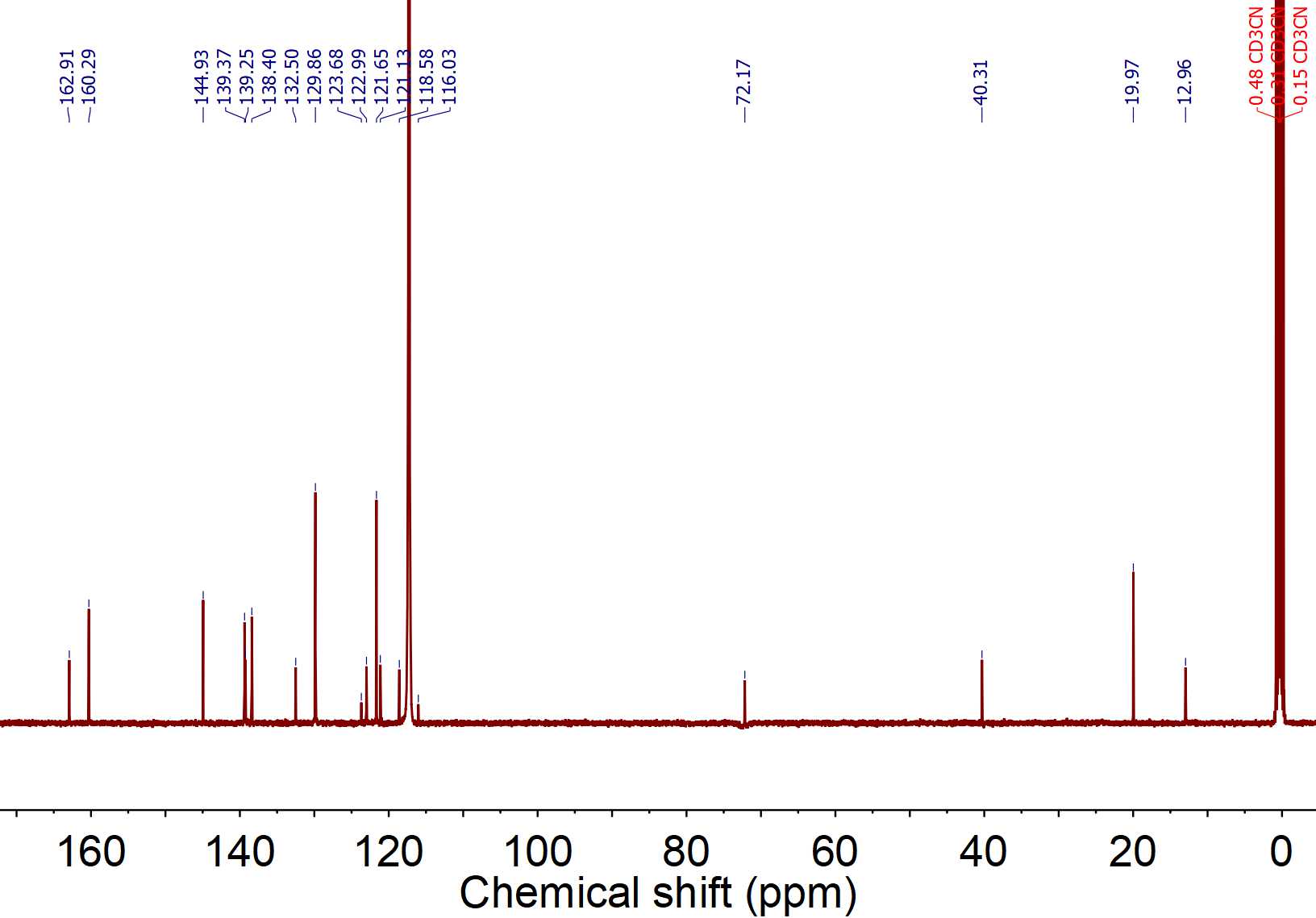


**Figure S27**. ^13^C NMR spectrum (500 MHz, 298 K, CD_3_CN) of **6**.


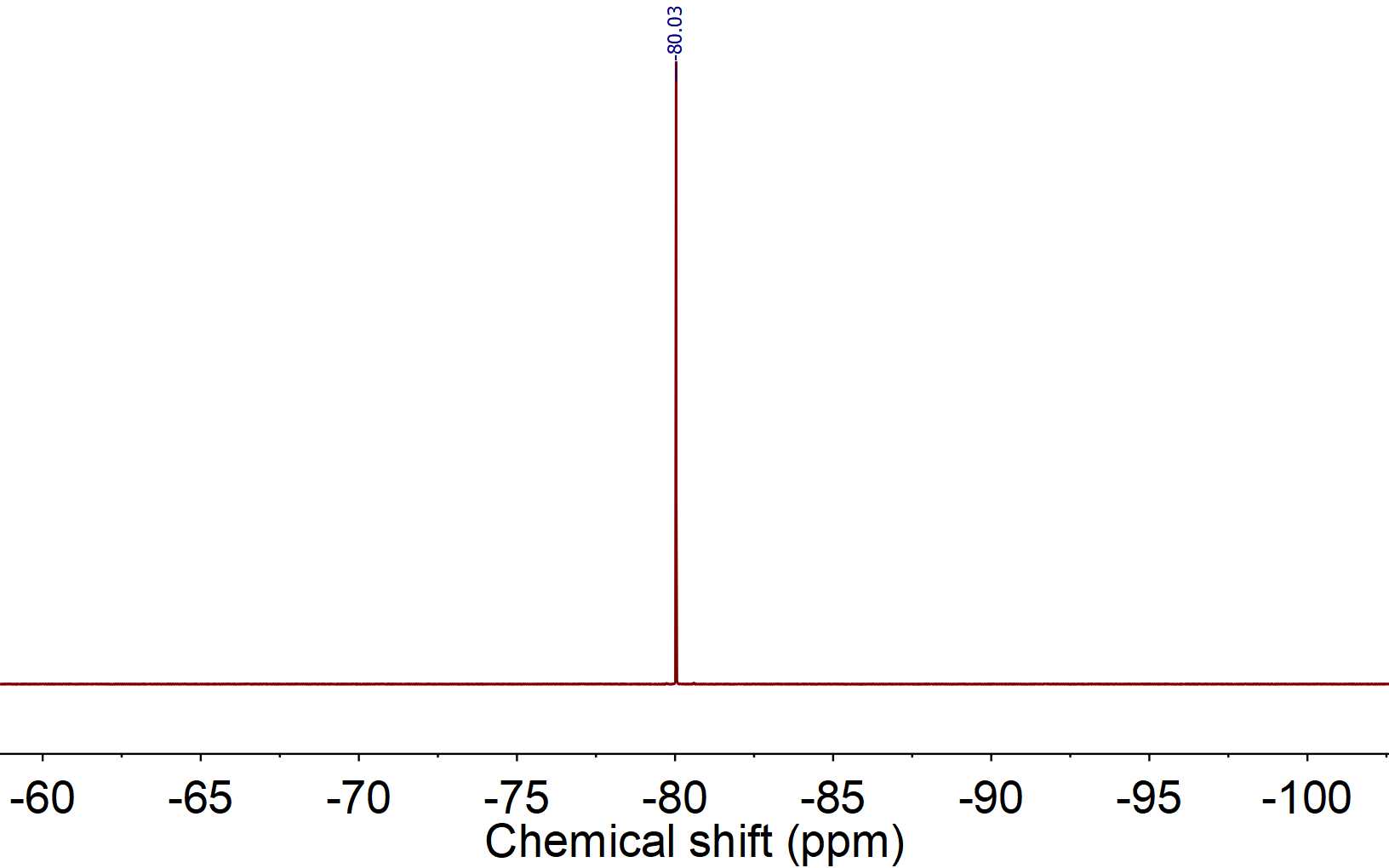


**Figure S28.** ^19^F NMR spectrum (376 MHz, 298 K, CD_3_CN) of **6**. δ –80.03 (s, CF_3_).


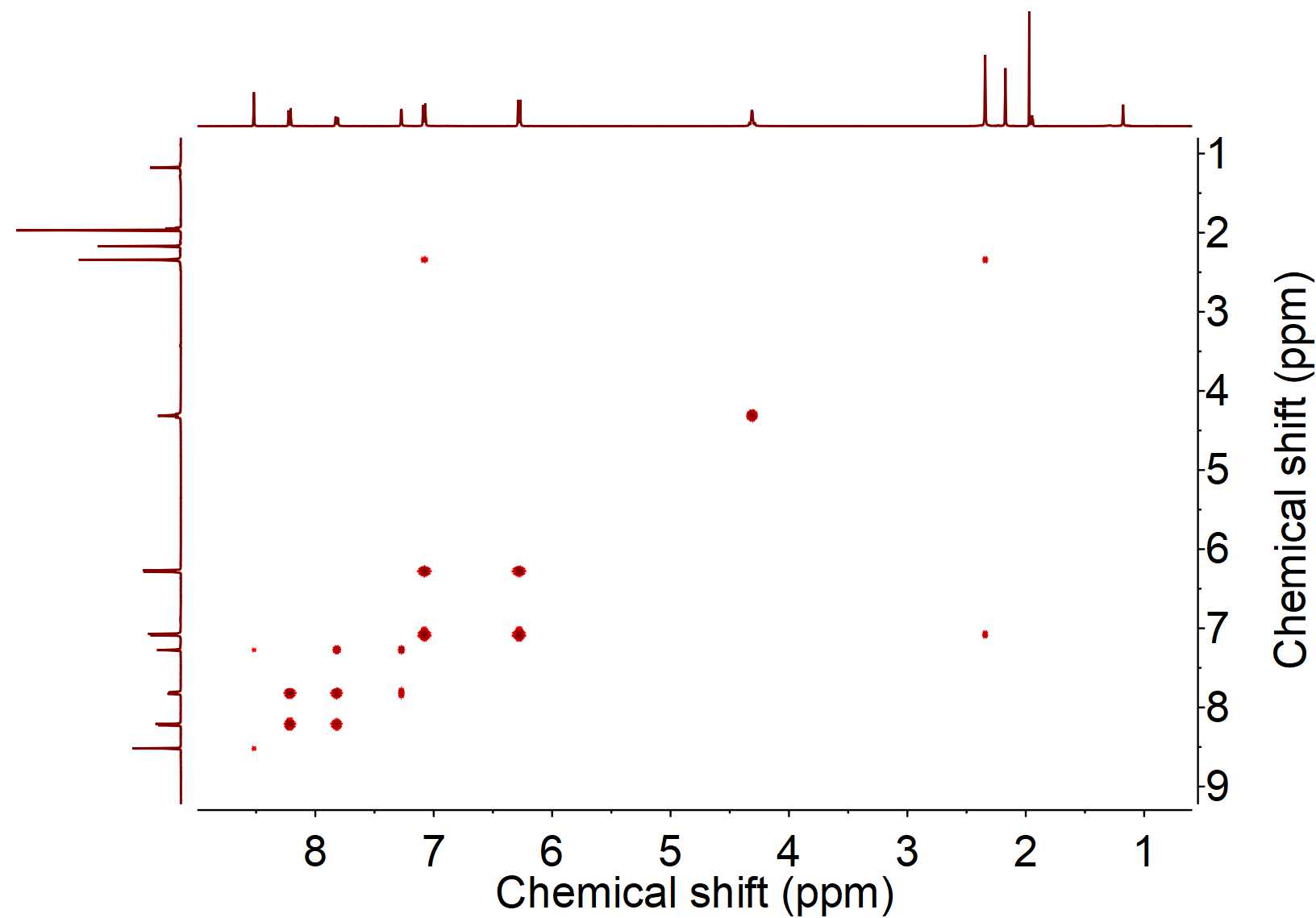


**Figure S29.** ^1^H–^1^H COSY spectrum (500 MHz, 298 K, CD_3_CN) of **6**.


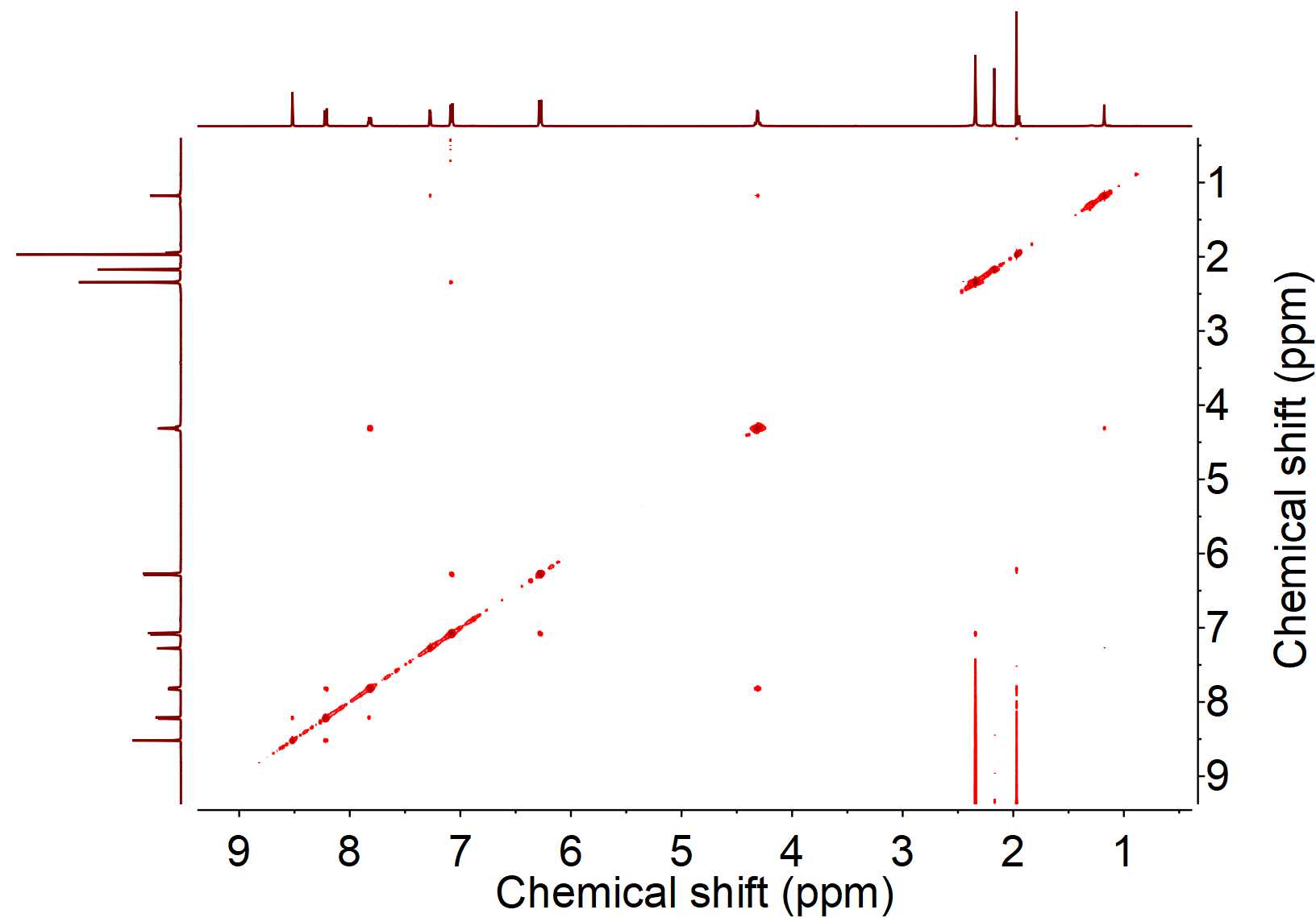


**Figure S30.** ^1^H–^1^H NOESY spectrum (500 MHz, 298 K, CD_3_CN) of **6**.


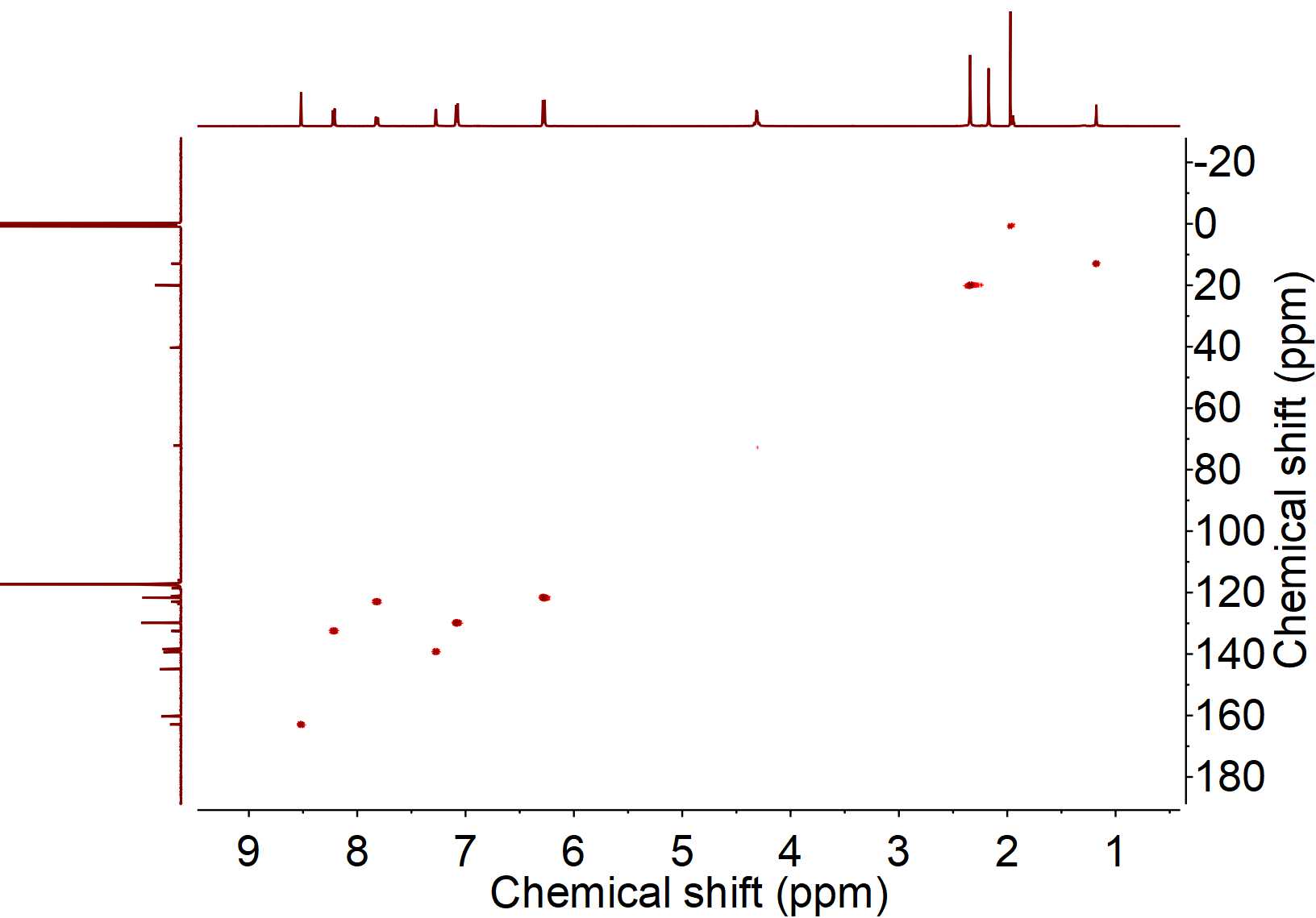


**Figure S31.** ^1^H–^13^C HSQC spectrum (500 MHz, 298 K, CD_3_CN) of **6**.


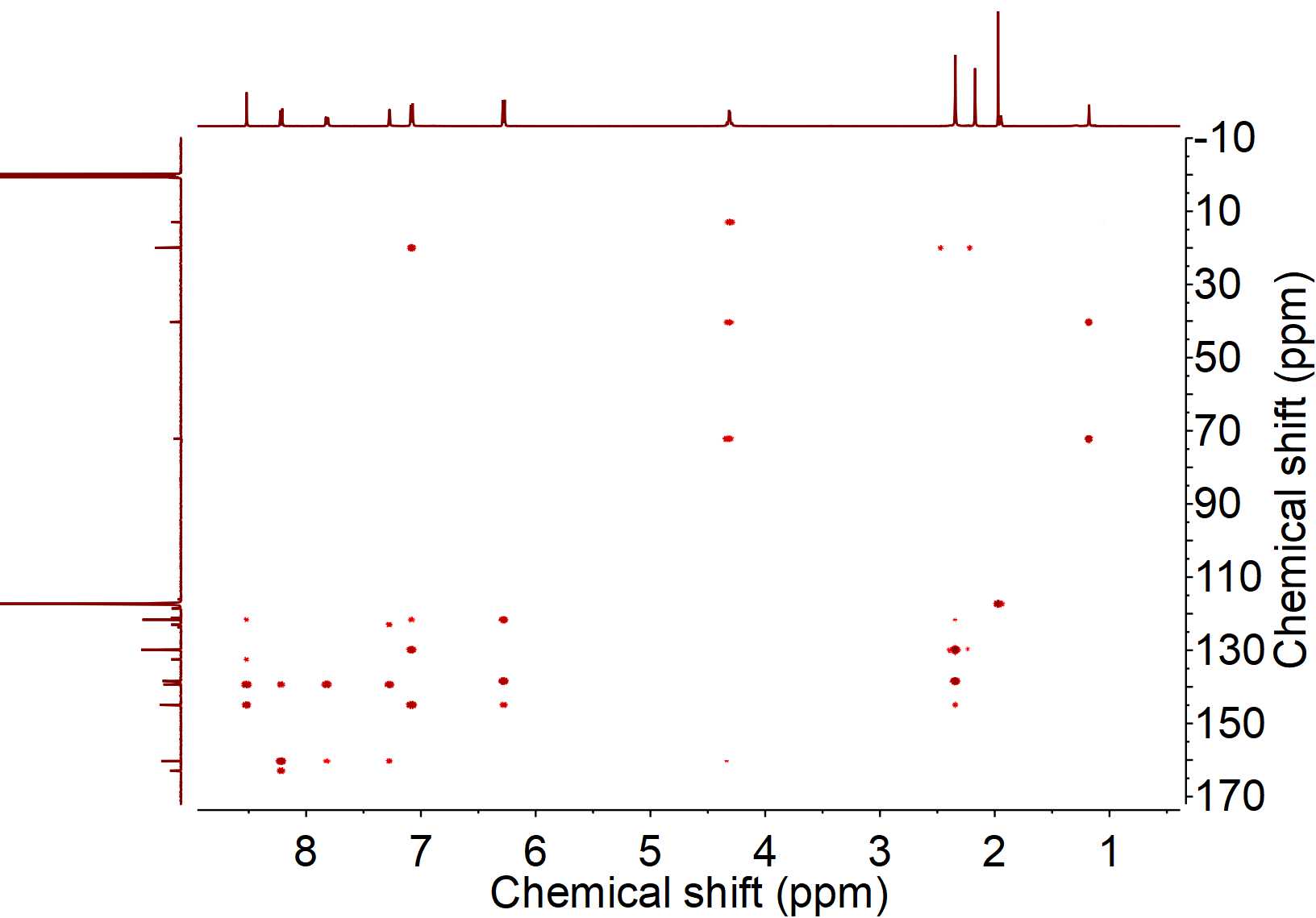


**Figure S32.** ^1^H–^13^C HMBC spectrum (500 MHz, 298 K, CD_3_CN) of **6**.


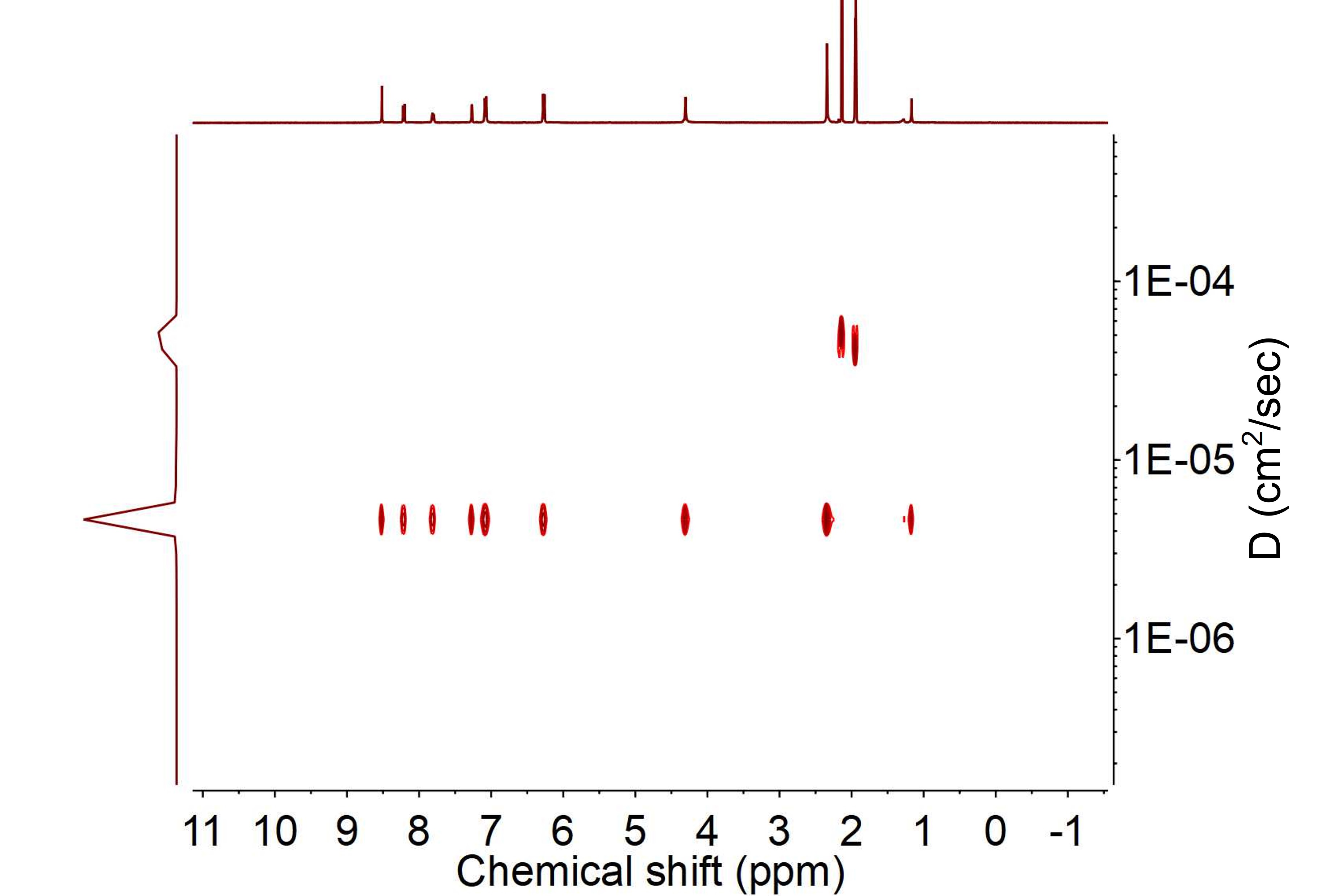


**Figure S33.** ^1^H DOSY spectrum (400 MHz, 298 K, CD_3_CN) of **6**. Diffusion coefficient: D = 4.68 × 10^–10^ m^2^·s^–1^, with solvodynamic radius of 14 Å.


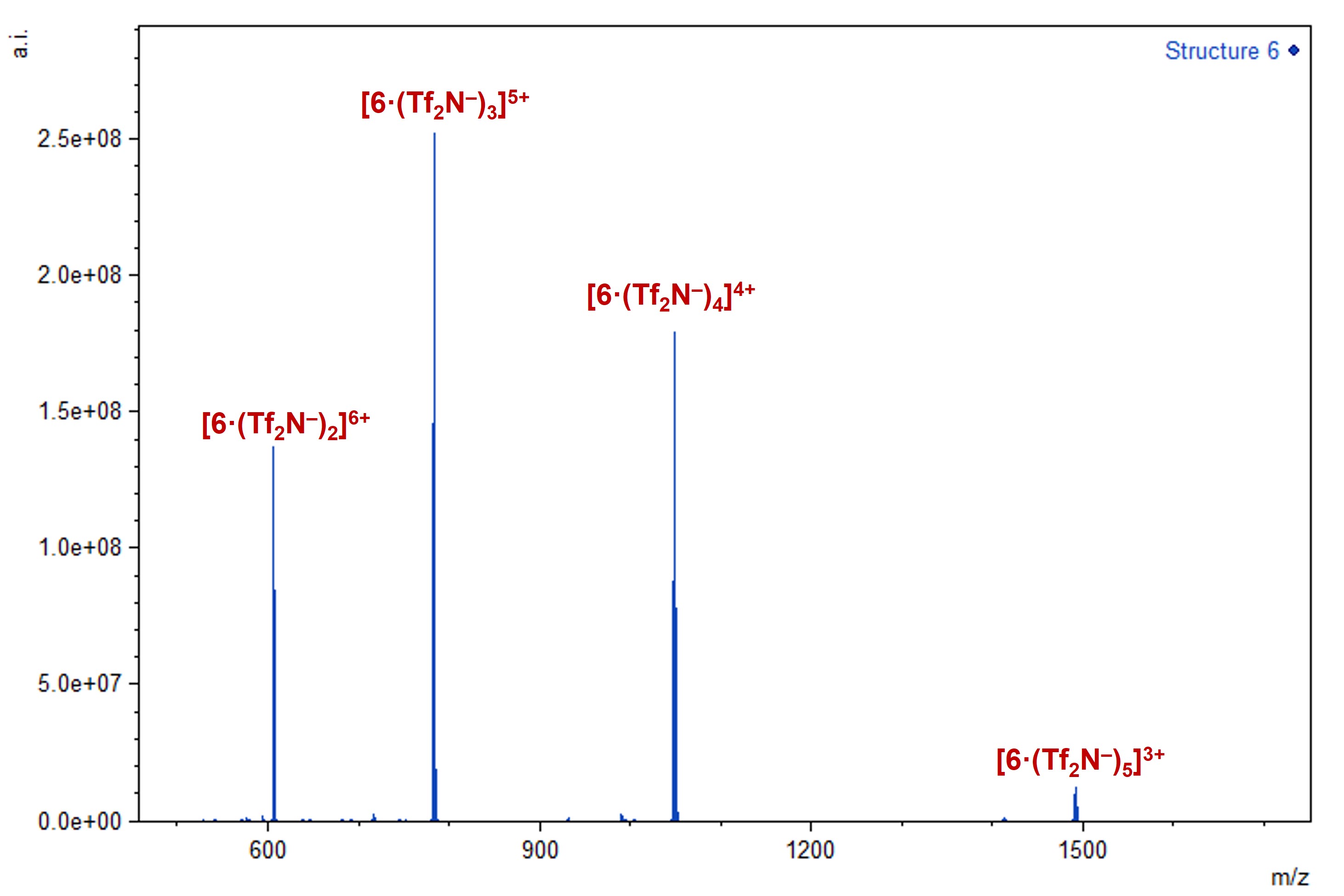


**Figure S34.** Low-resolution ESI-MS spectrum of **6**. *m*/*z* = 605.5 [**6**·(Tf_2_N^–^)_2_]^6+^,782.6 [**6**·(Tf_2_N^–^)_3_]^5+^,1048.2 [**6**·(Tf_2_N^–^)_4_]^4+^, 1490.9 [**6**·(Tf_2_N^–^)_5_]^3+^. Calculated peaks: *m*/*z* = 605.5 [**6**·(Tf_2_N^–^)_2_]^6+^,782.7 [**6**·(Tf_2_N^–^)_3_]^5+^,1048.4 [**6**·(Tf_2_N^–^)_4_]^4+^, 1491.2 [**6**·(Tf_2_N^–^)_5_]^3+^.


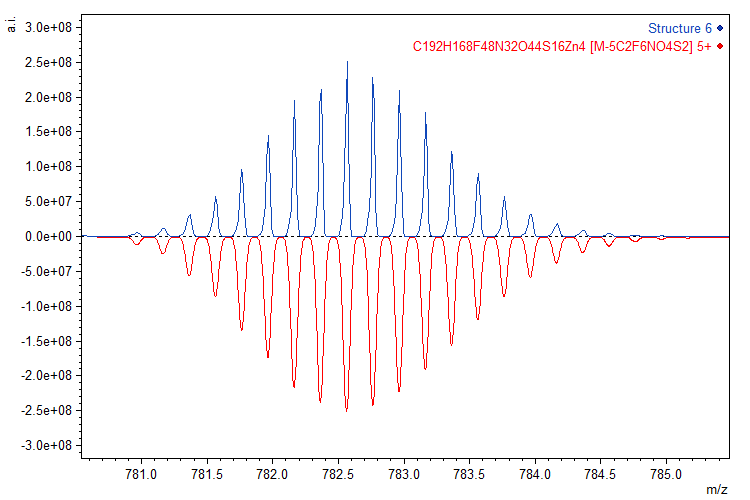


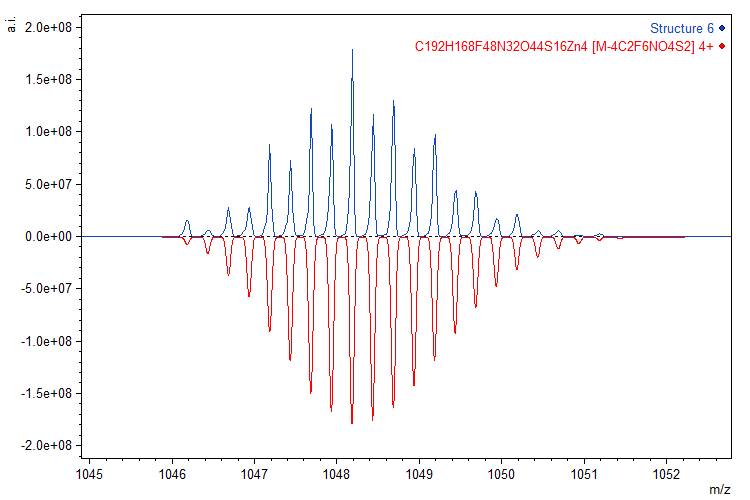


**Figure S35.** High-resolution ESI-mass spectrometry analysis of **6** showing the observed (top blue) and theoretical (bottom red) isotope patterns for the 5+ and 4+ peaks.

3.4 Self-assembly between Subcomponent **B** and **D**


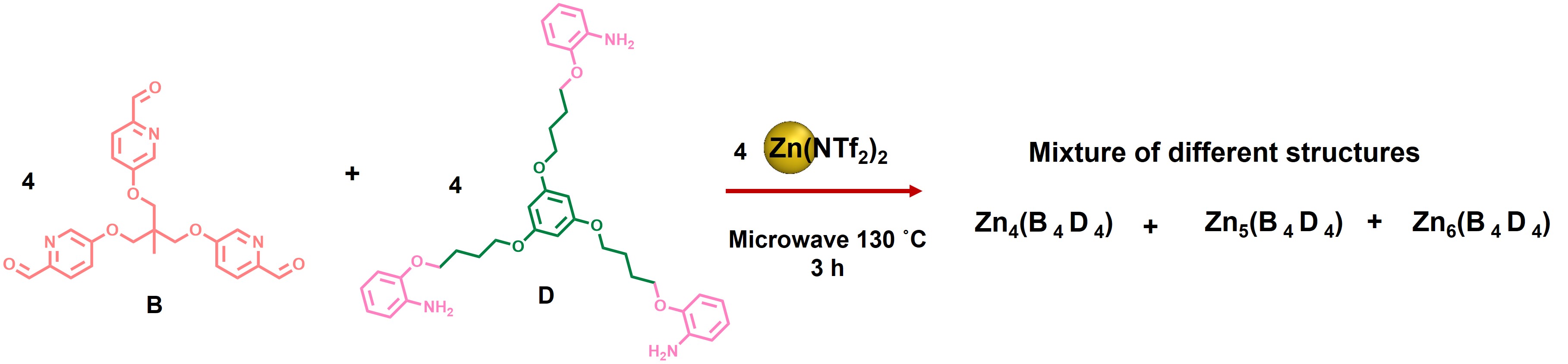


**Scheme S6.** Self-assembly between Subcomponent **B** and **D**

Subcomponent **B** (2.0 mg, 4.6 µmol, 4 equiv.) reacted with Subcomponent **D** (2.9 mg, 4.6 µmol, 4 equiv.) and zinc(II) bis(trifluoromethanesulfonimide) (2.9 mg, 4.6 µmol, 4 equiv.) in acetonitrile (2 mL) under microwave irradiation for 3 h at 130 ˚C. Then the mixture was concentrated to 0.4 mL and diethyl ether (14 mL) was added. The precipitate was collected by centrifugation and washed with excess diethyl ether to give the product mixture.


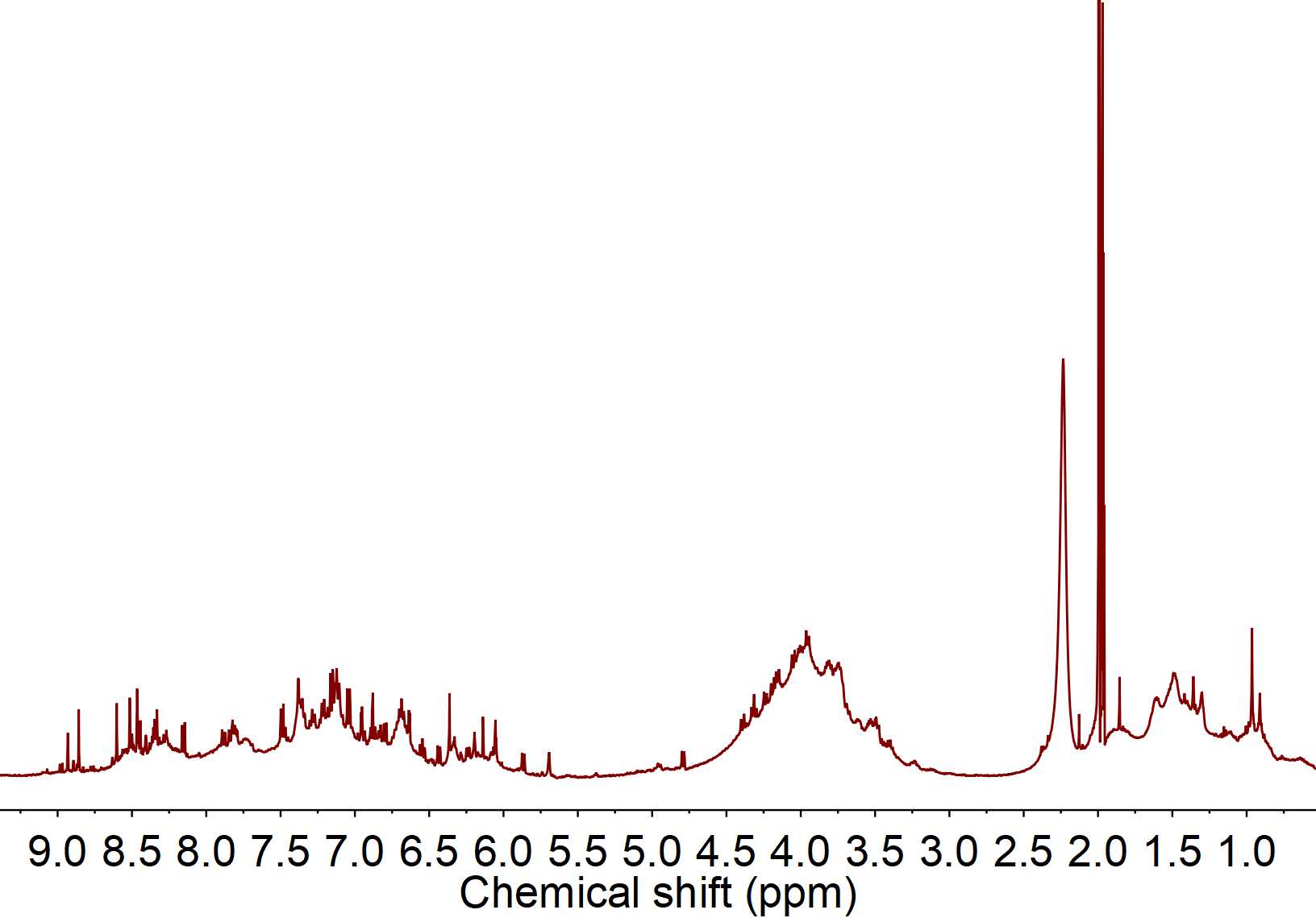


**Figure S36**. ^1^H NMR spectra (400 MHz, CD_3_CN) of self-assembly between Subcomponent **B** and **D** with Zn(Tf_2_N)_2_ shows many sharp and broad signals, indicating the formation of a mixture of different species.


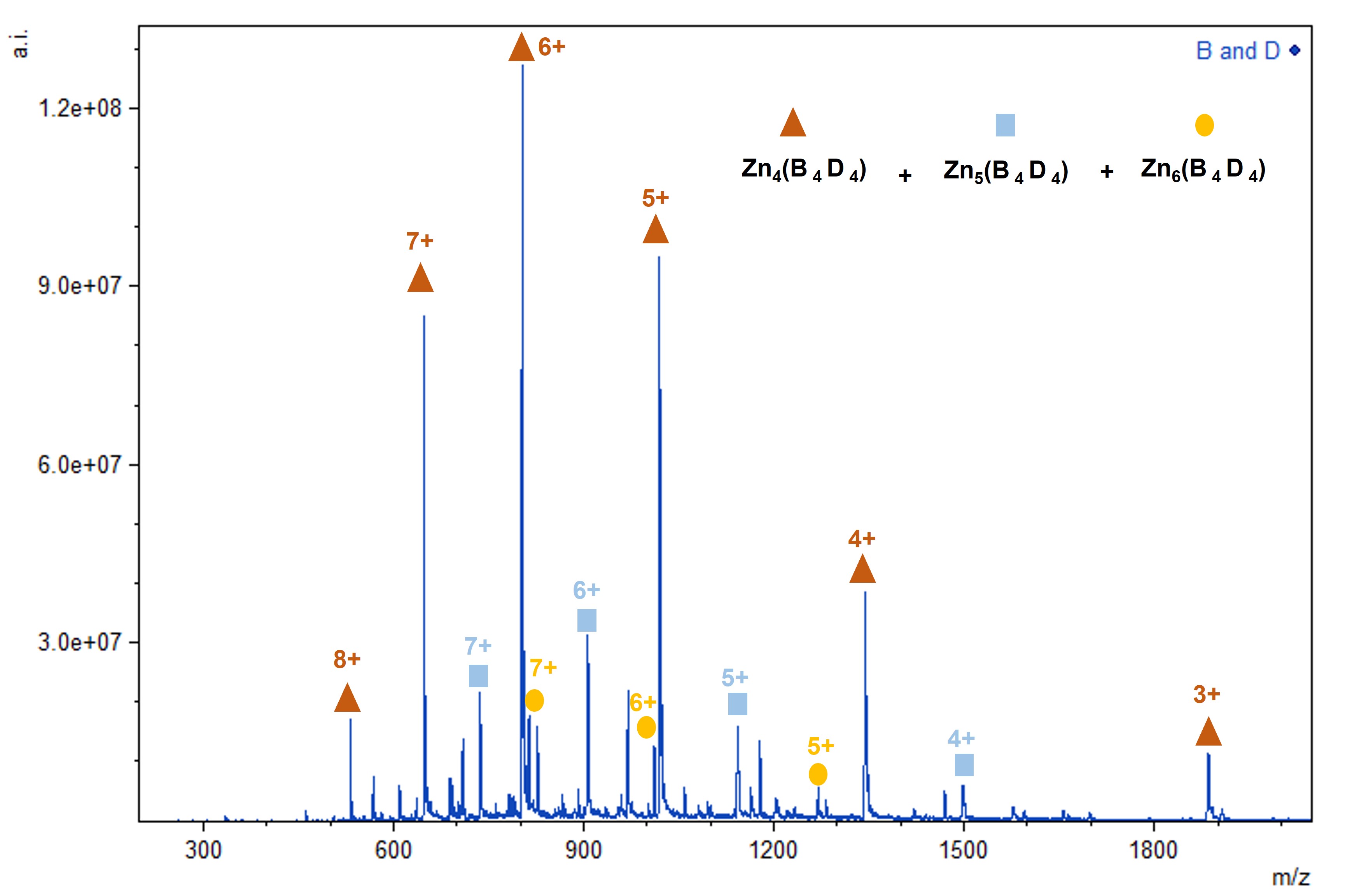


**Figure S37**. Low-resolution ESI-MS spectrum of the mixture product. Signals belonging to Zn_4_(B_4_D_4_), Zn_5_(B_4_D_4_) and Zn_6_(B_4_D_4_) were observed.

# **4. Structural transformations between 1 and 5**

### 4.1 Structural transformation from **1** to **5**


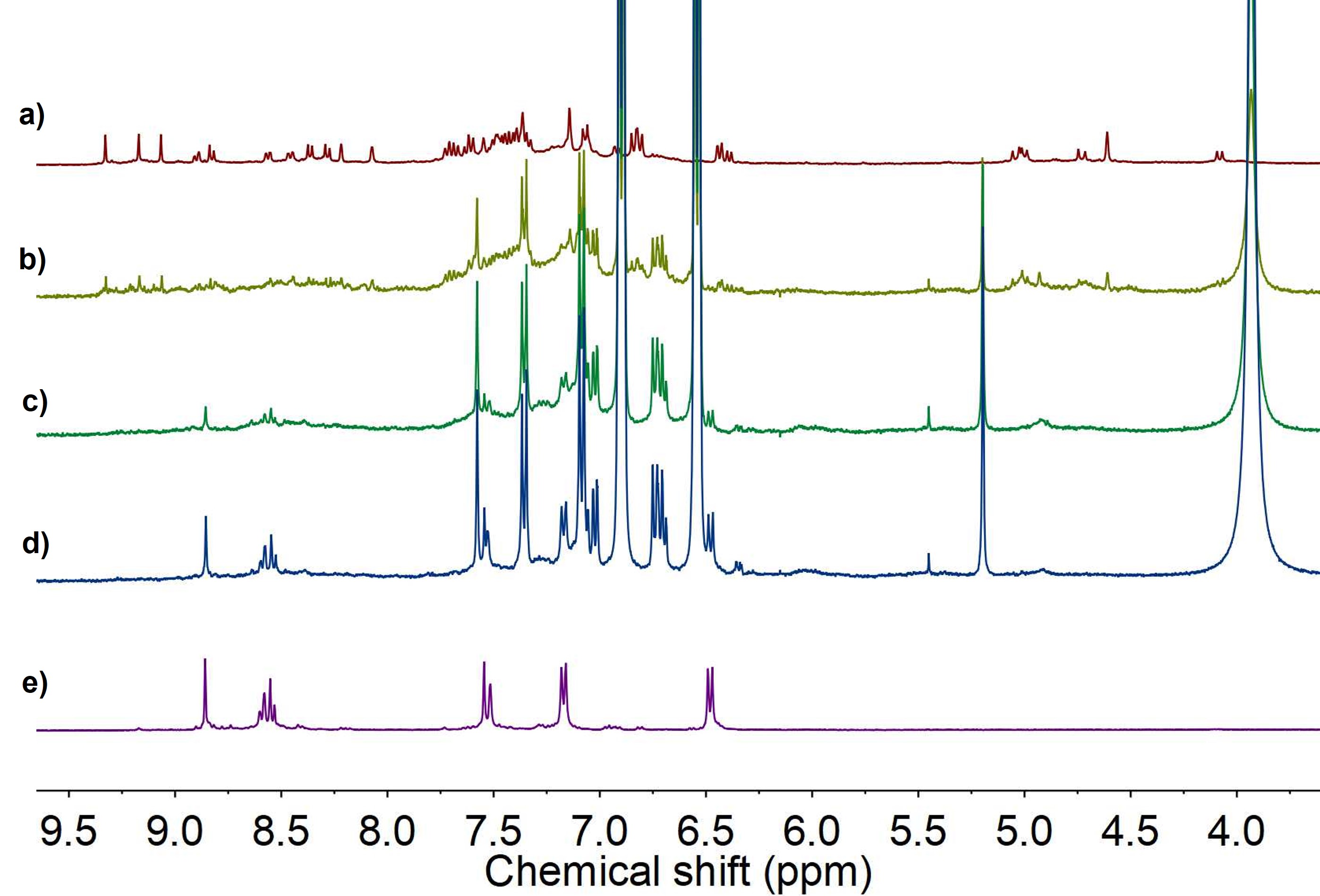


**Figure S38**. ^1^H NMR spectra (400 MHz, CD_3_CN) comparison of **a)** 0.5 mM **1**; **b)** 0.5 mM **1** with addition of 2 equiv. *p*-toluidine per imine; **c)** 0.5 mM **1** with addition of 4 equiv. *p*-toluidine per imine; **d)** 0.5 mM **1** with addition of 6 equiv. *p*-toluidine per imine; **e)** Standard cage **5** (0.5 mM). All of transformations in **b)**, **c)** and **d)** were carried out at 120 ˚C for 2 hours under microwave irradiation.

### 4.2 Structural transformation from **5** to **1**


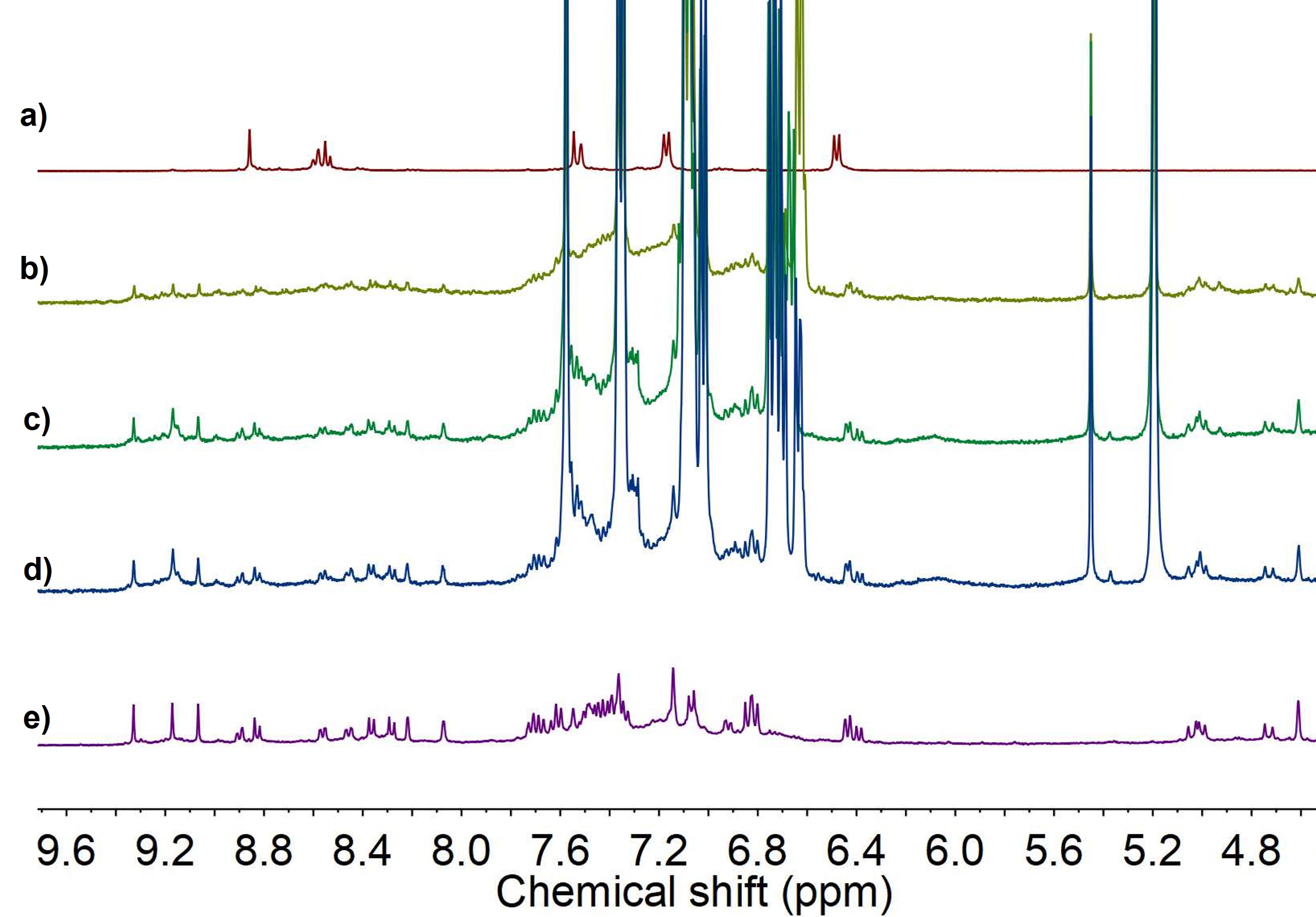


**Figure S39**. ^1^H NMR spectra (400 MHz, CD_3_CN) comparison of **a)** 0.5 mM **5**; **b)** 0.5 mM **5** with addition of 4 equiv. **C** per cage; **c)** 0.5 mM **5** with addition of 8 equiv. **C** per cage; **d)** 0.5 mM **5** with addition of 12 equiv. **C** per cage; **e)** Standard **1** (0.5 mM). All of transformations in **b)**, **c)** and **d)** were carried out at 120 ˚C for 2 hours under microwave irradiation.

# **5. Host-guest investigations of 1**

### 5.1 General methods

The host-guest chemistry of **1** andguests **G1**-**G4** was investigated as shown in scheme S7. Host-guest complexes were prepared on an NMR scale and characterized by ^1^H NMR spectroscopy. A solution of **1** in CD_3_CN (0.5 mM) was transferred to an NMR tube and guests **G1** and **G2** was gradually added as a stock solution in CD_3_CN. The NMR tube was shaken for 5 minutes after each addition, and then the ^1^H NMR spectrum was measured.


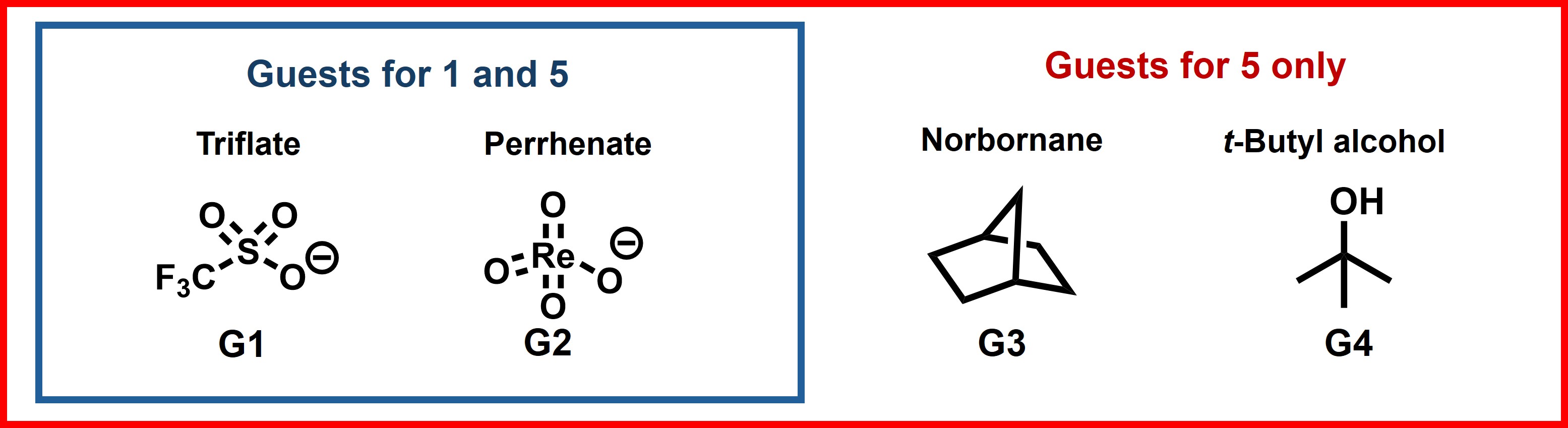


**Scheme S7.** Guests bound within **1** (deep blue framework) and **5** (red framework).

### 5.2 NMR spectra from host-guest investigations

5.2.1 Addition of **G3** and **G4** to **1**


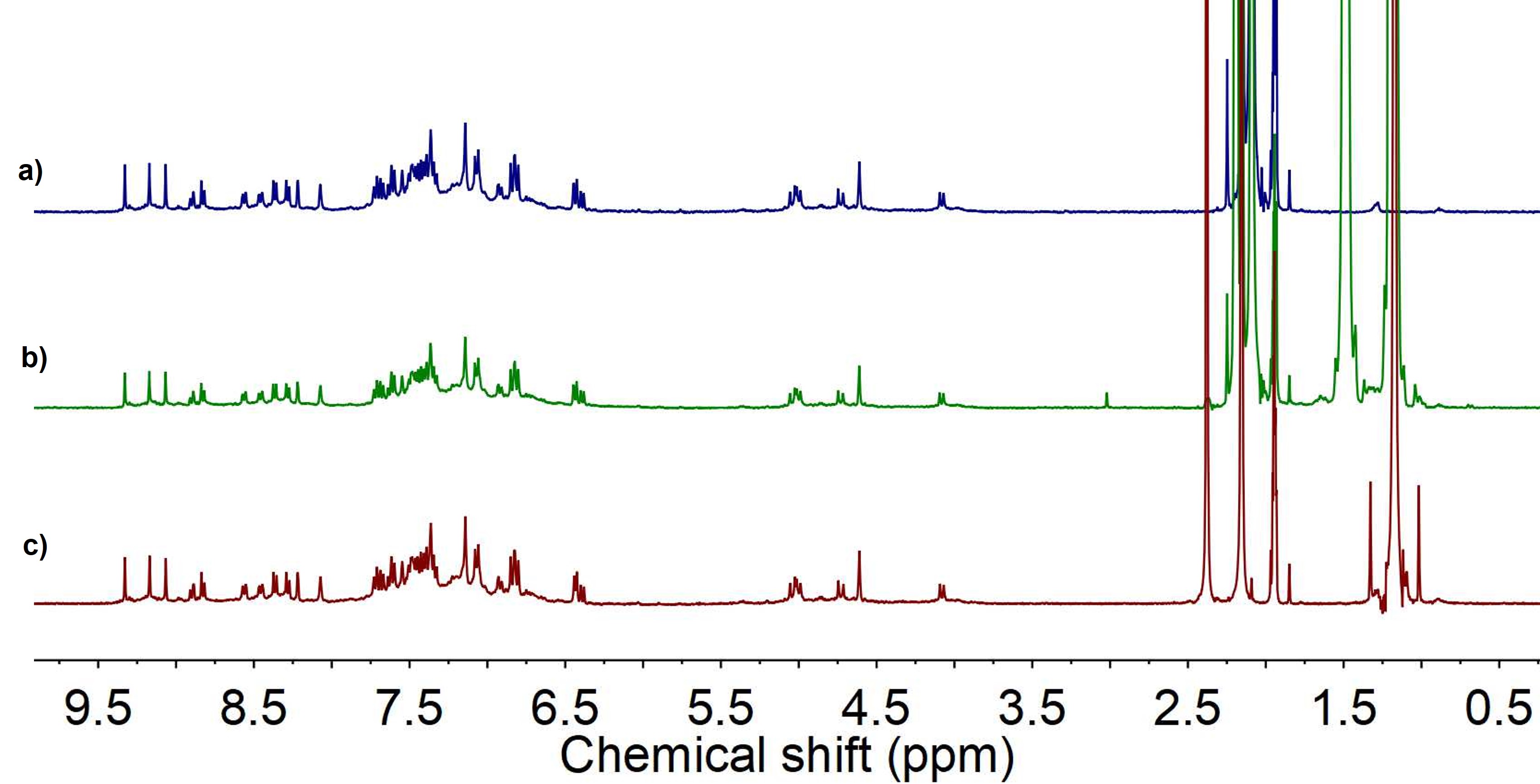


**Figure S40**.^1^H NMR spectra (400 MHz, CD_3_CN) of **a)** 0.5 mM **1**; **b)** 0.5 mM **1** with addition of 40 equiv. **G3**; **c)** 0.5 mM **1** with addition of 40 equiv. **G4**. No significant host-guest binding was observed.

5.2.2 NMR titrations of **G1** and **G2** with **1**


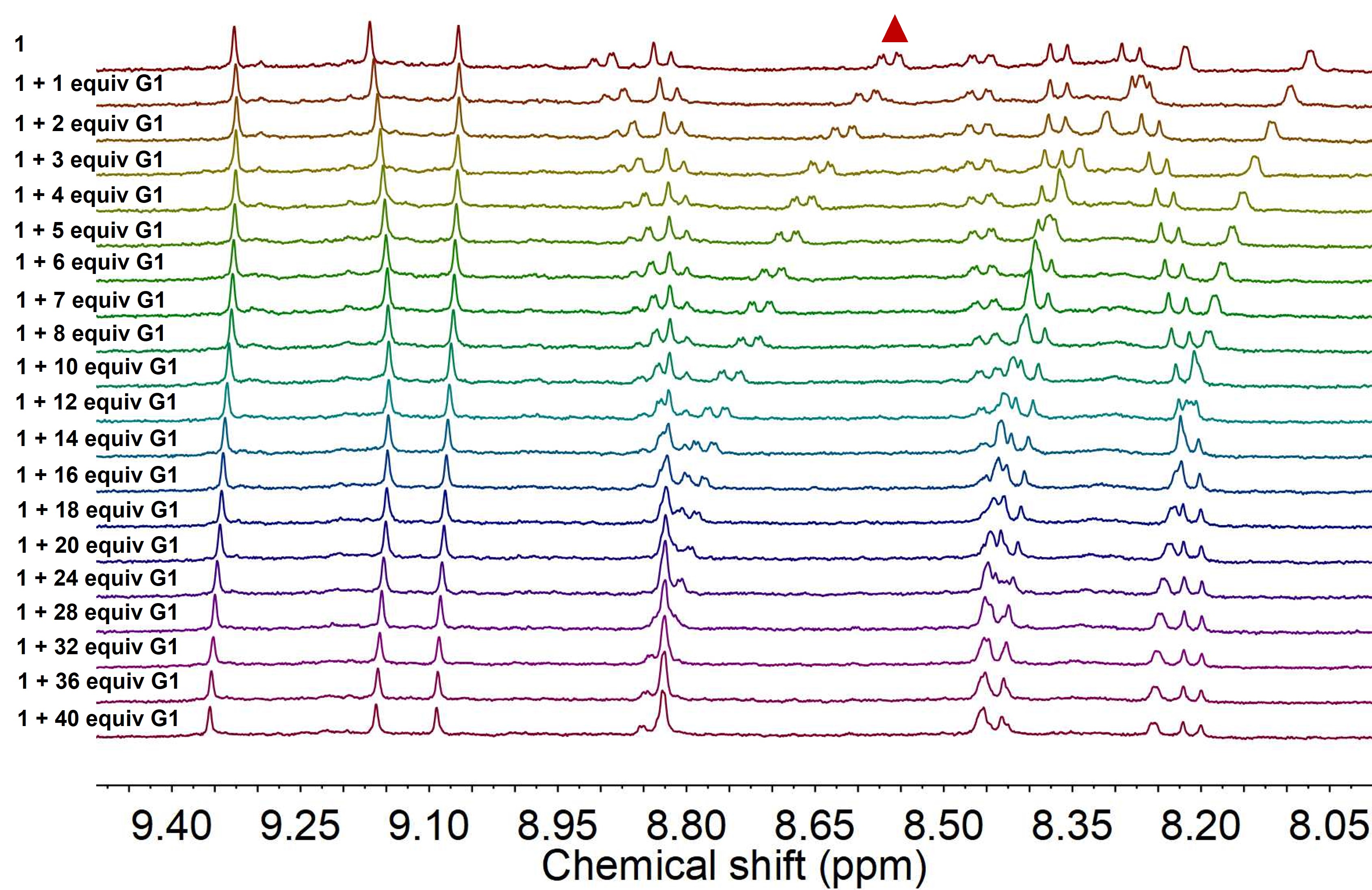


**Figure S41**. Partial ^1^H NMR spectra recorded during the titration (400 MHz, 298 K) of **G1** into a solution of **1** in CD_3_CN (equivalents of **G1** are labelled on the left).


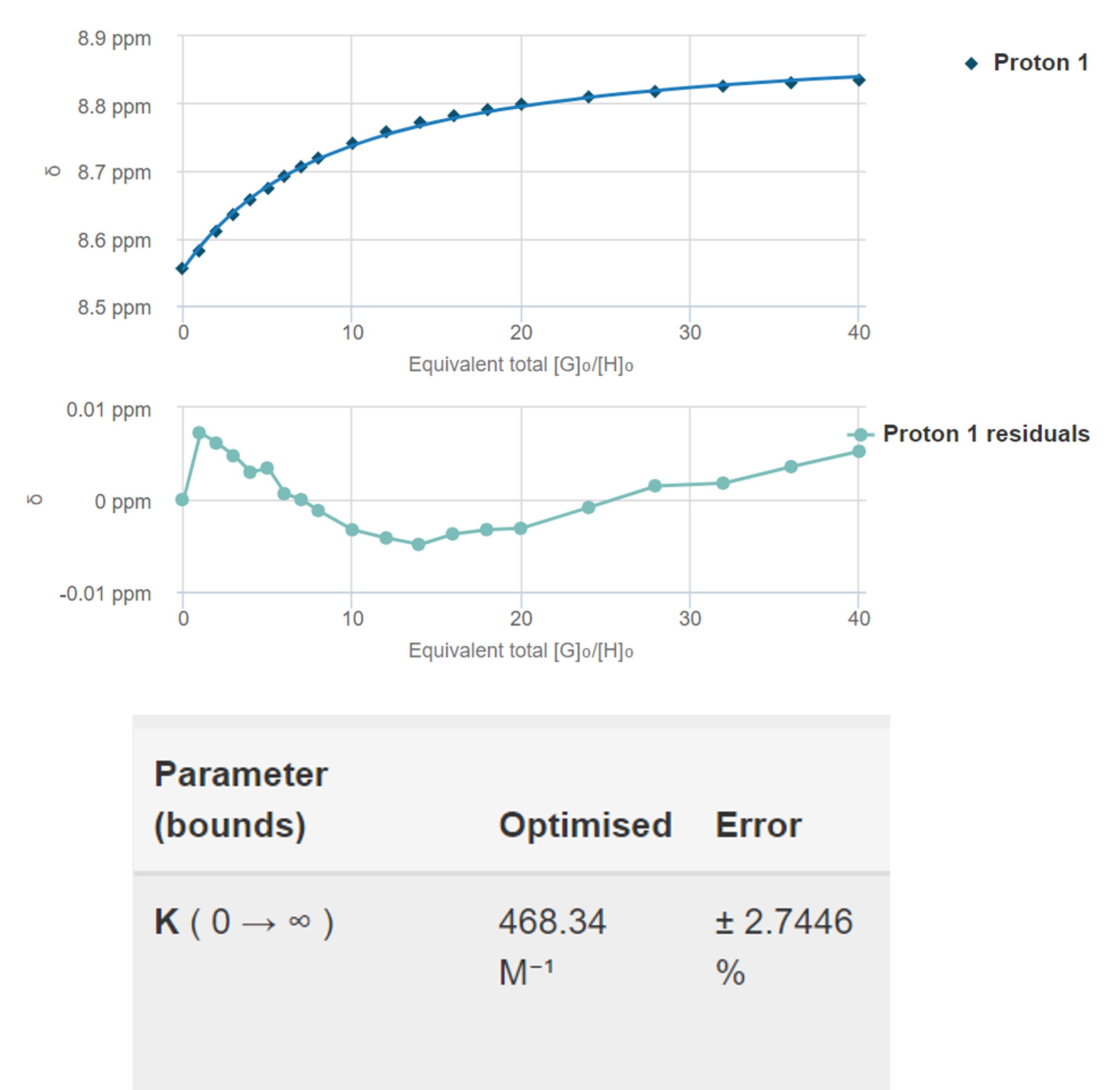


**Figure S42**. The NMR titration data fitted well to a 1:1 host-guest binding model with a binding constant of (4.7 ± 0.1) × 10^2^ M^-1.^ Attempts to fit the titration data to different binding models, such as 1:2, 1:3 and 1:4 produced worse fits than the 1:1 host-guest model.


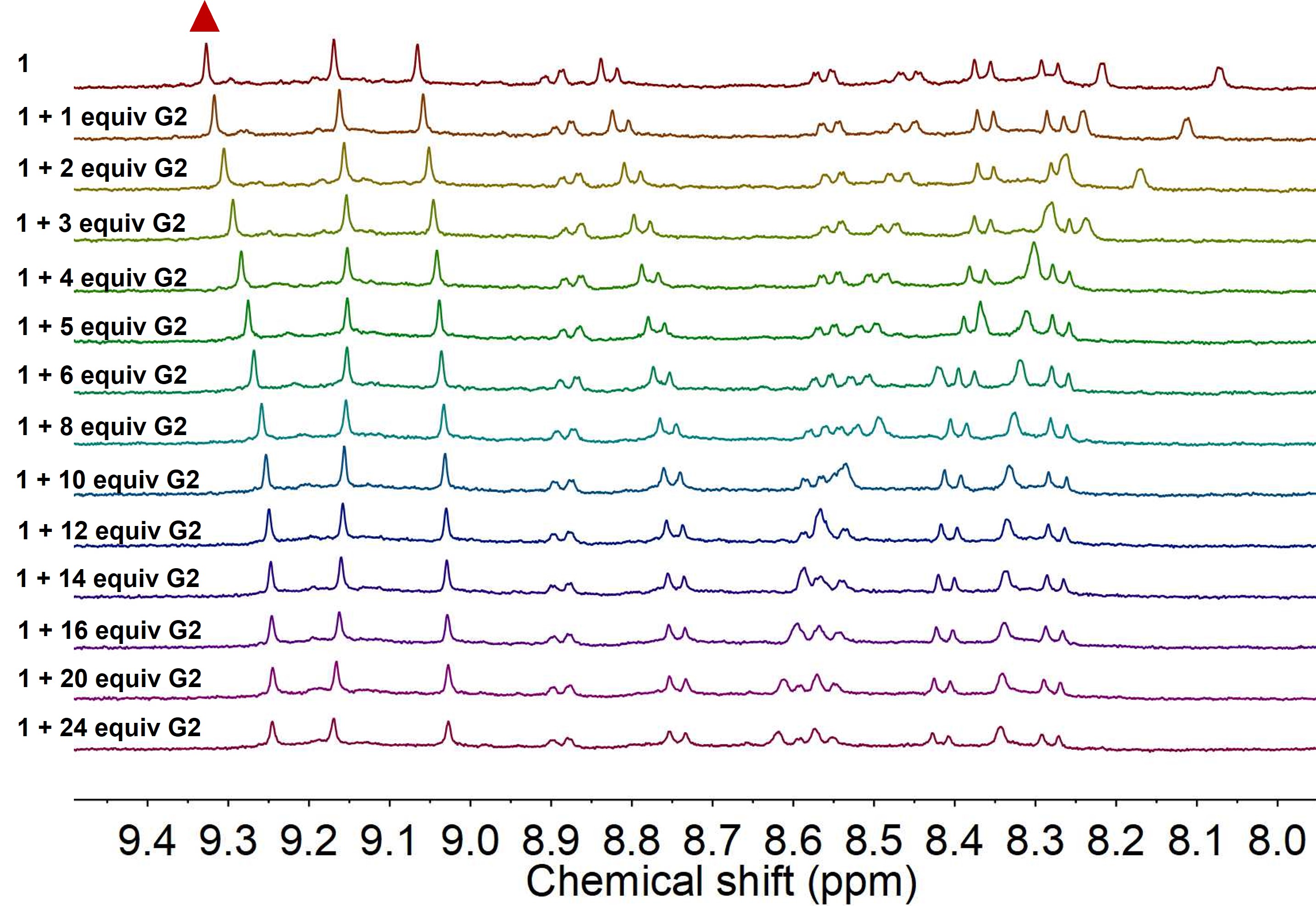


**Figure S43**. Partial ^1^H NMR spectra recorded during the titration (400 MHz, 298 K) of **G2** into a solution of **1** in CD_3_CN (equivalents of **G2** are labelled on the left).


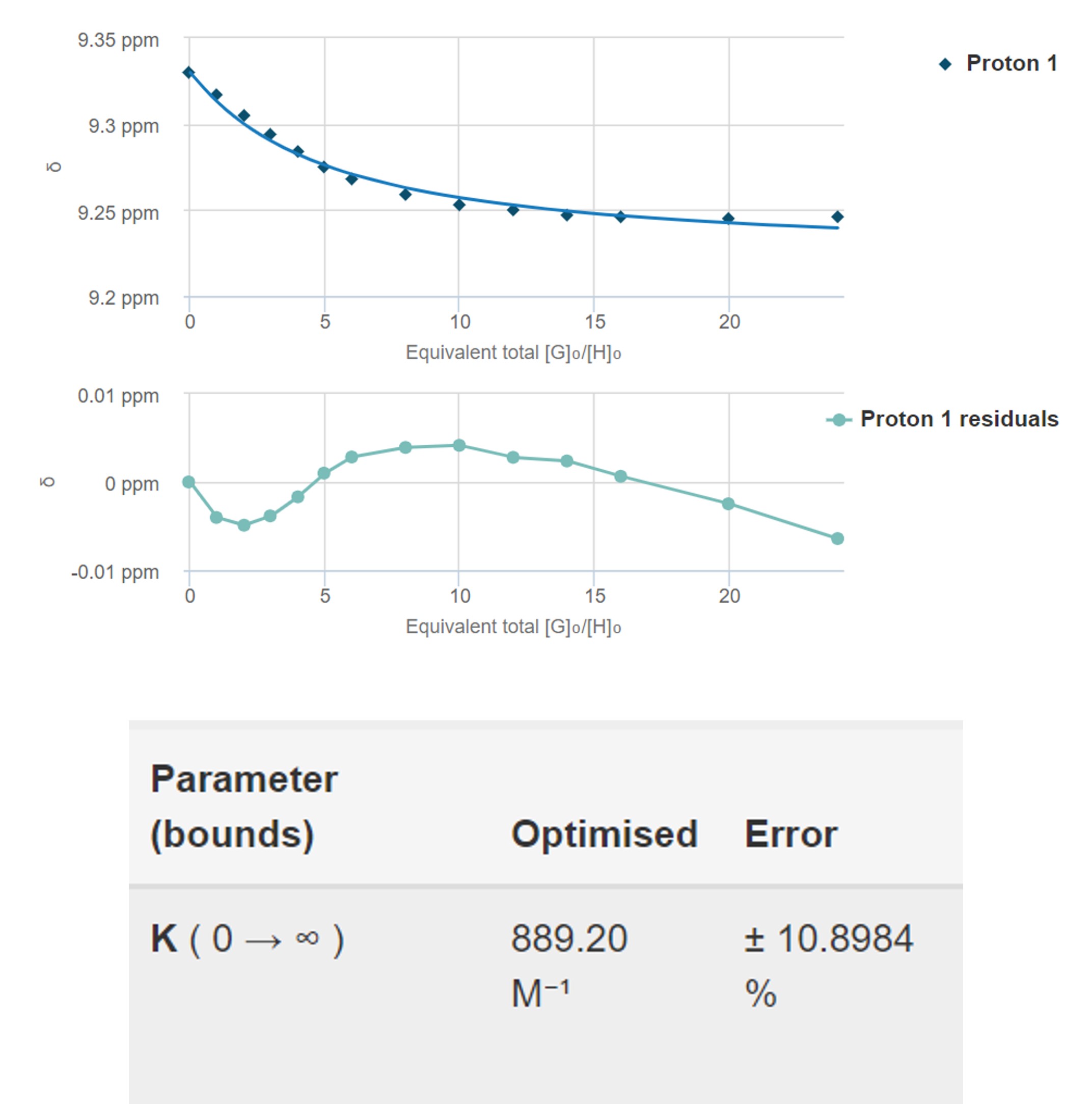


**Figure S44**. The NMR titration data fitted well to a 1:1 host-guest binding model with a binding constant of (8.9 ± 0.9) × 10^2^ M^-1.^ Attempts to fit the titration data to different binding models, such as 1:2, 1:3 and 1:4 produced worse fits than the 1:1 host-guest model.

# **6. Guest release and encapsulation induced by structural transformations between 1 and 5**

6.1 Release and encapsulation of **G3** via structural transformations between **1** and **5**


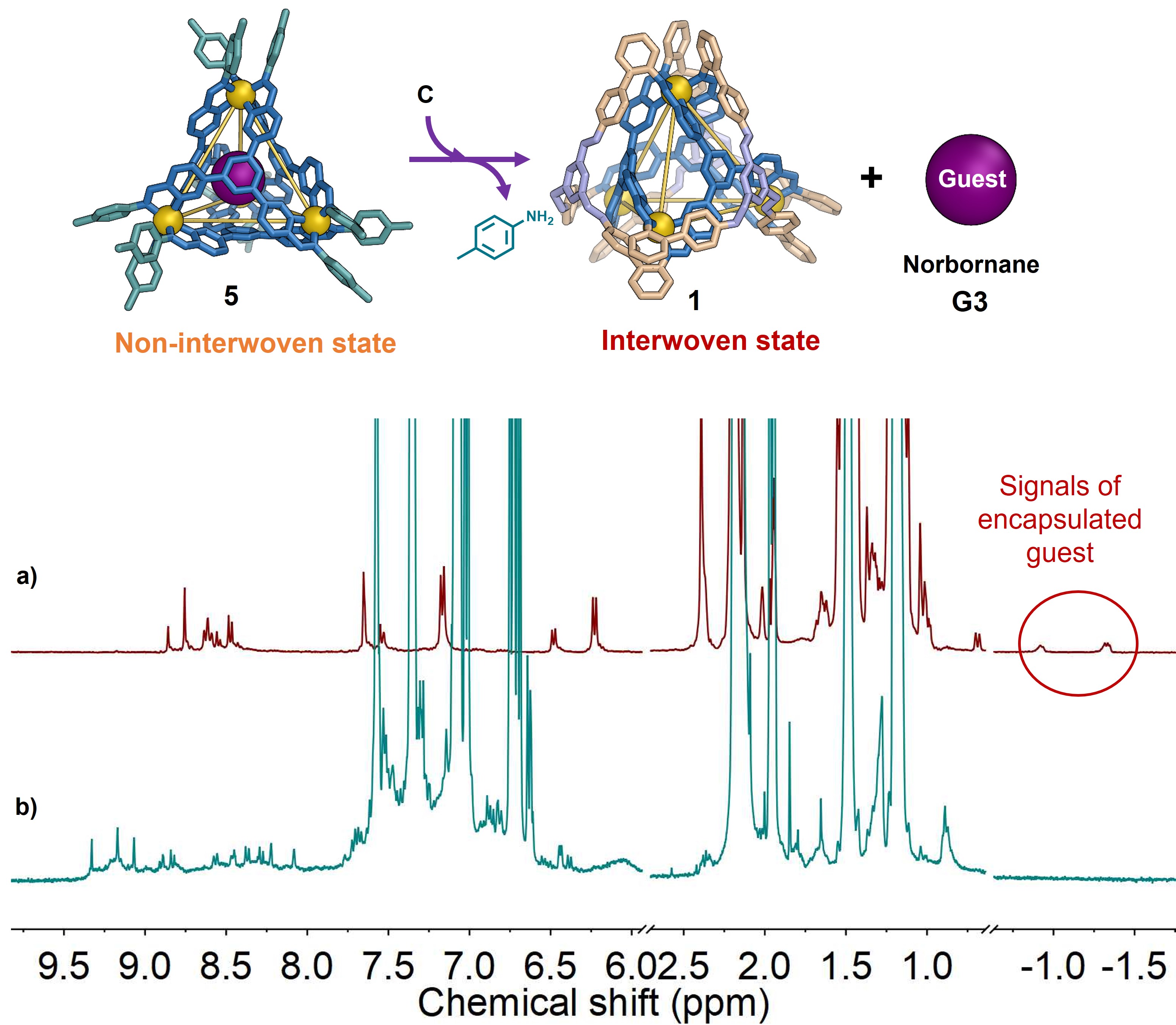


**Figure S45**. ^1^H NMR spectra (400 MHz, CD_3_CN) comparison of **a)** 0.5 mM **G3⊂5** (20 mM **G3)**; **b)** A 0.5 mM solution of **G3⊂5** with the addition of 15 equiv. of **C** per cage following reaction under microwave irradiation at 120 °C for 2 hours, resulting in formation of **1** with the release of **G3**.


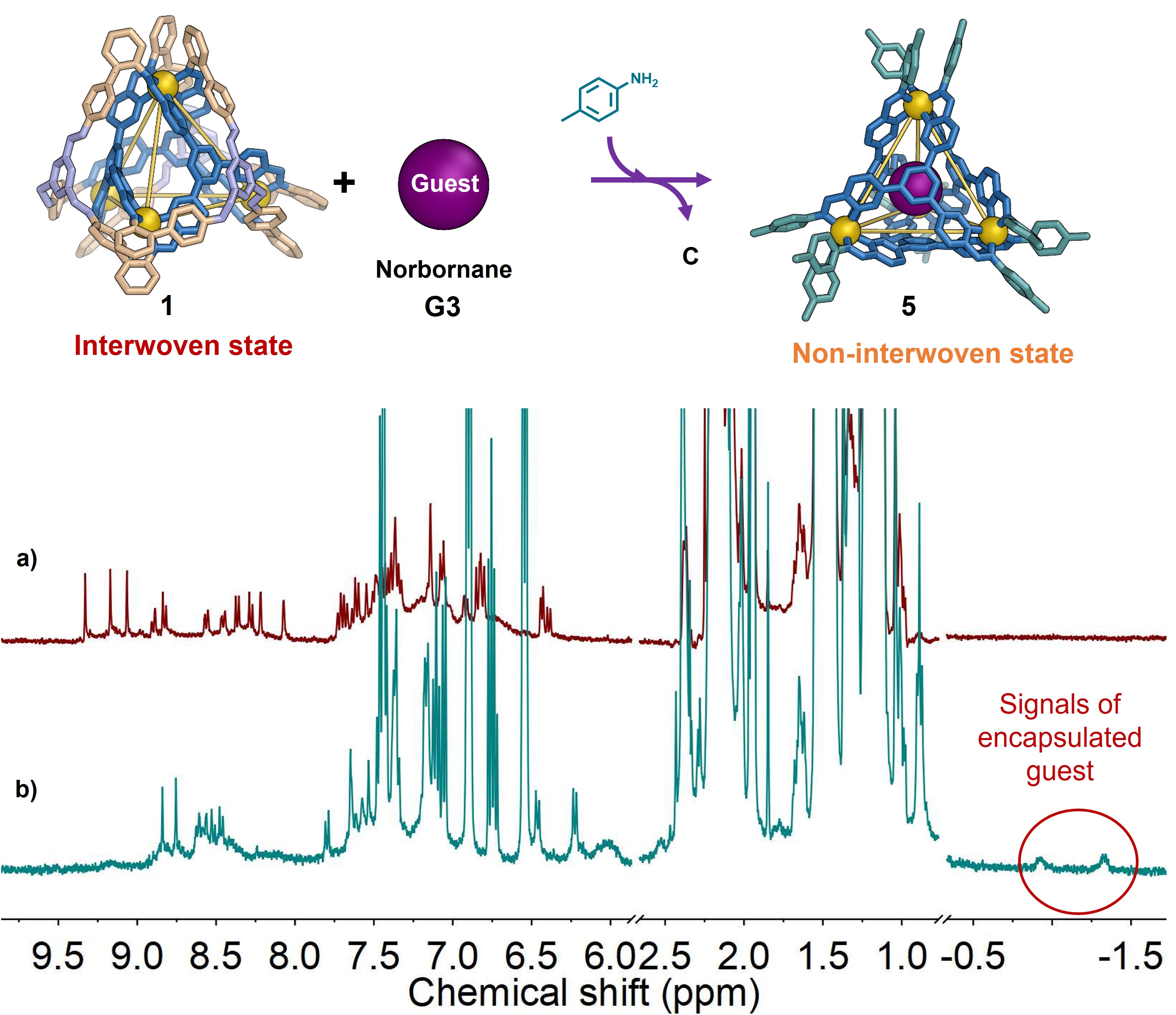


**Figure S46**. ^1^H NMR spectra (400 MHz, CD_3_CN) comparison of **a)** 0.5 mM **1** with 40 equiv. **G3**; **b)** with the addition of 6 equiv. *p*-toluidine per imine following reaction under microwave irradiation at 120 °C for 2 hours, resulting in formation of **5** with encapsulation of **G3**.


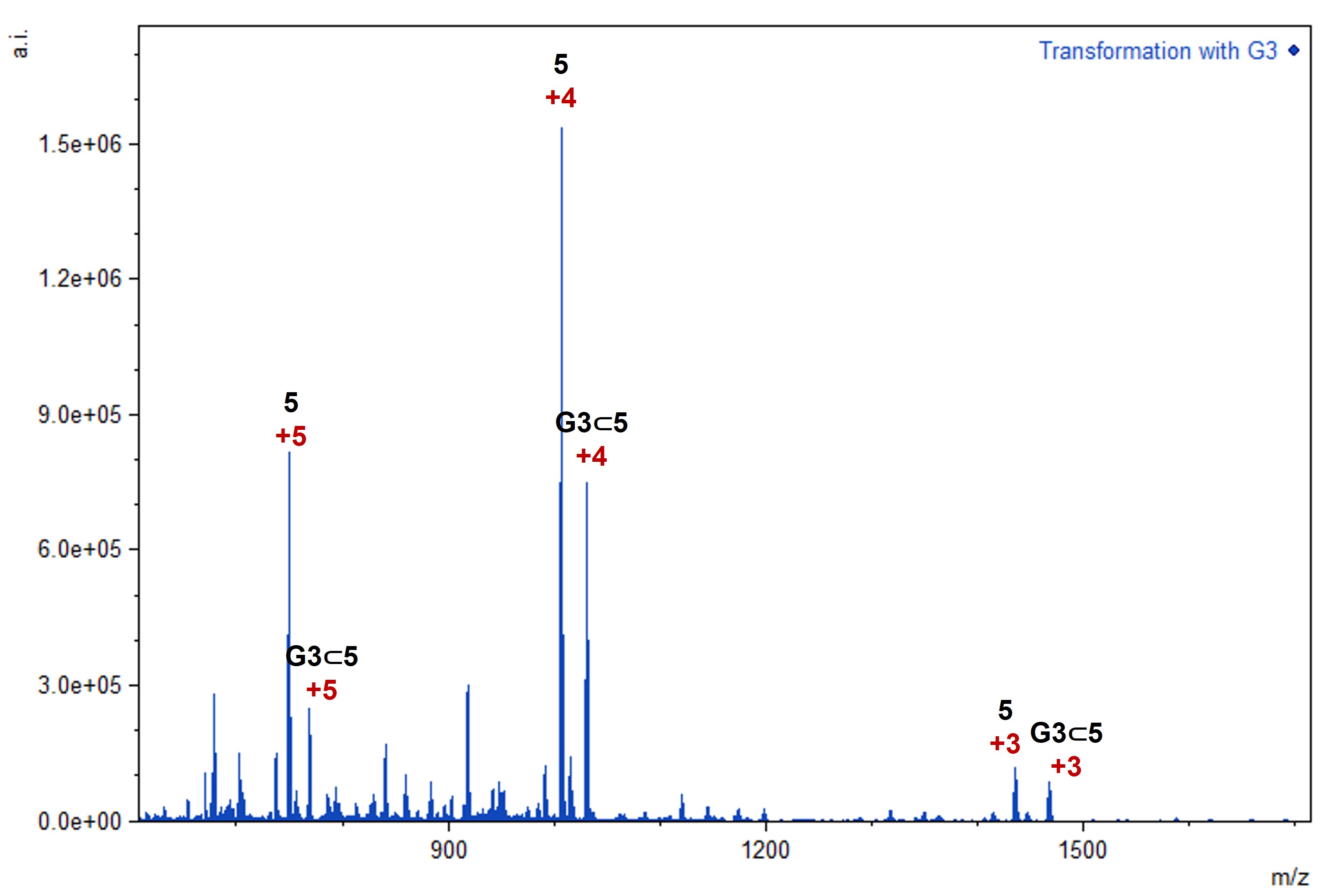


**Figure S47**. ESI-MS analysis of the product from **Figure S46 b)**.

6.2 Release and encapsulation of **G4** via structural transformations between **1** and **5**


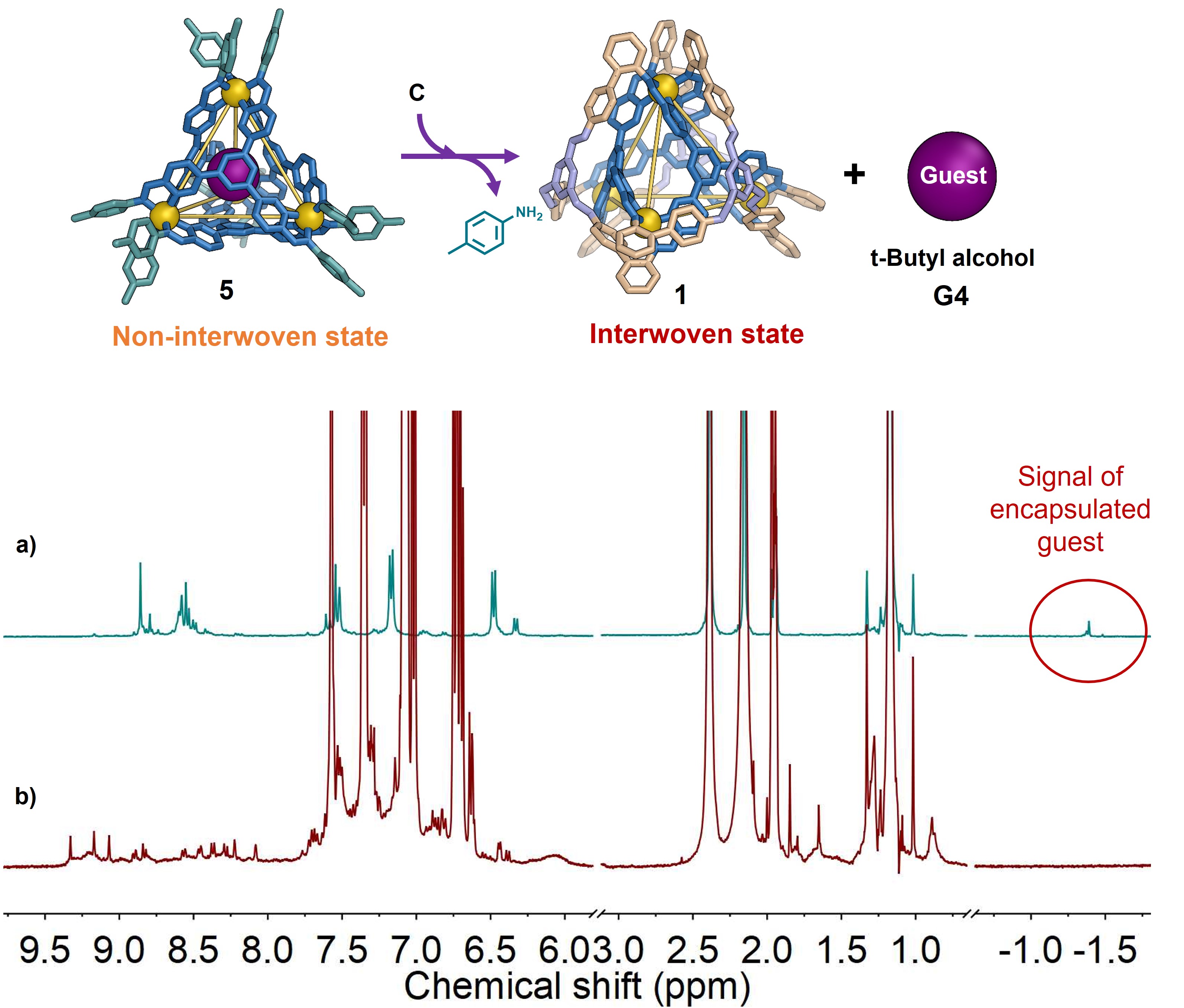


**Figure S48**. ^1^H NMR spectra (400 MHz, CD_3_CN) comparison of **a)** 0.5 mM **G4⊂5**; **b)** A 0.5 mM solution of **G4⊂5** with the addition of 15 equiv. of **C** per cage following reaction under microwave irradiation at 120 °C for 2 hours, resulting in formation of **1** with the release of **G4**.


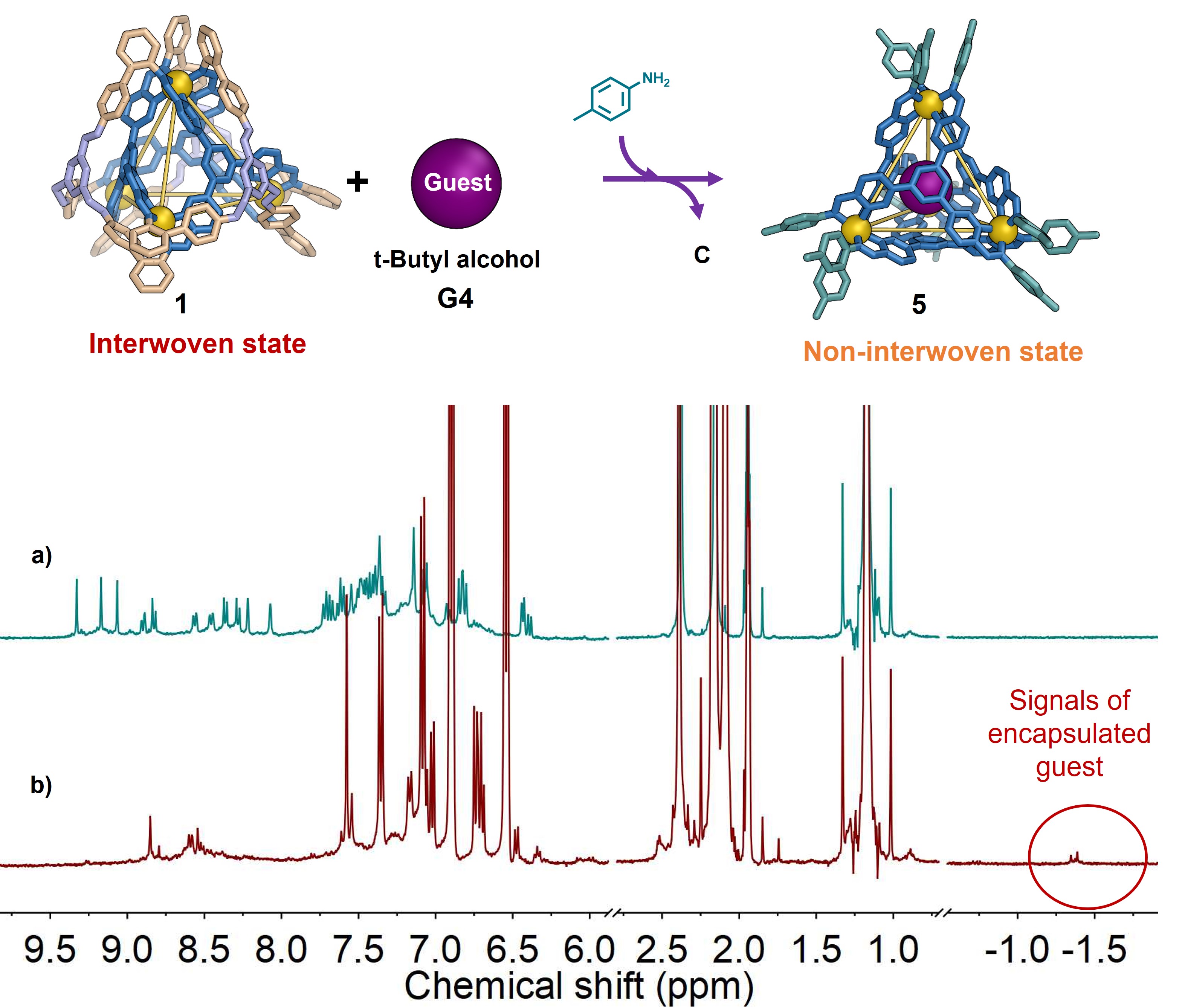


**Figure S49**. ^1^H NMR spectra (400 MHz, CD_3_CN) comparison of **a)** 0.5 mM **1** with 40 equiv. **G4**; **b)** with the addition of 6 equiv. *p*-toluidine per imine following reaction under microwave irradiation at 120 °C for 2 hours, resulting in formation of **5** with encapsulation of **G4**.


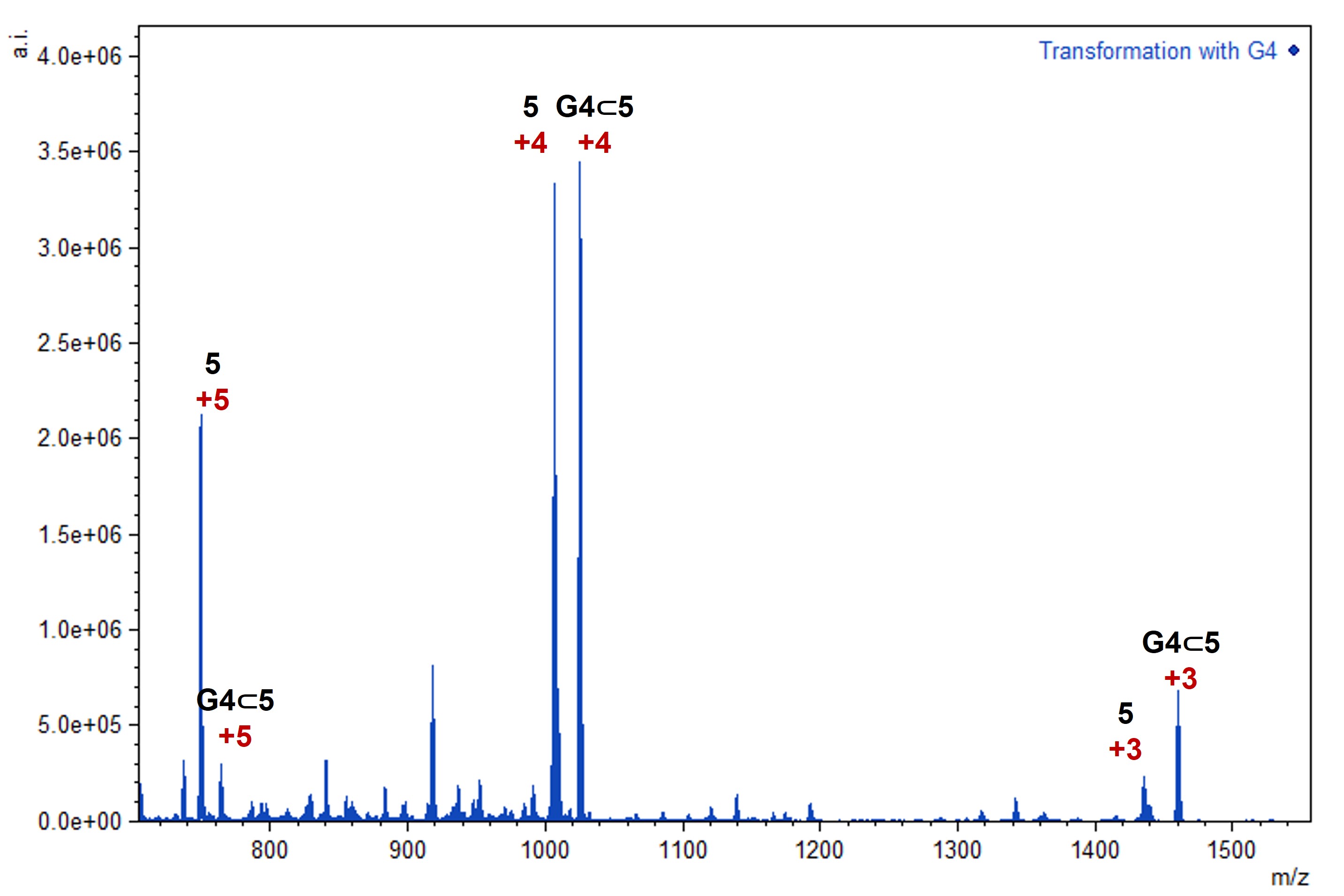


**Figure S50**. ESI-MS analysis of the product from **Figure S49 b)**.

# **7. Structural transformation of 4 and 6**

### 7.1 Triflate induced ligand reorientation in **6**


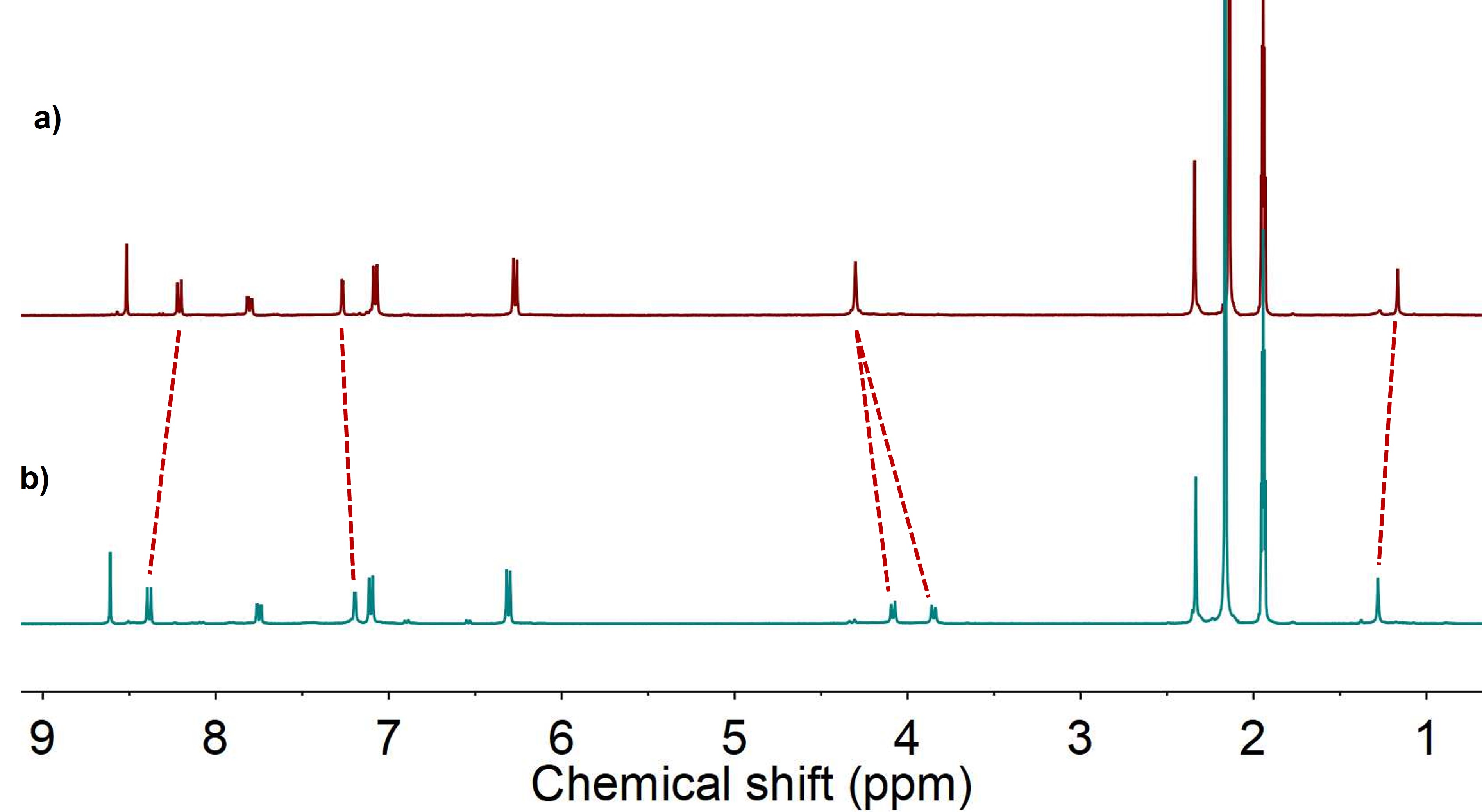


**Figure S51**. ^1^H NMR spectra (400 MHz, CD_3_CN) comparison of **a)** 0.4 mM **6**; **b)** 0.4 mM solution of **6** following the addition of 8 equiv. of KOTf and heating at 50 °C for 1 hour. Red dashed lines show chemical shifts in the NMR spectrum indicating outward reorientation of the methyl groups to facilitate guest encapsulation, forming TfO^–^⊂**7**.


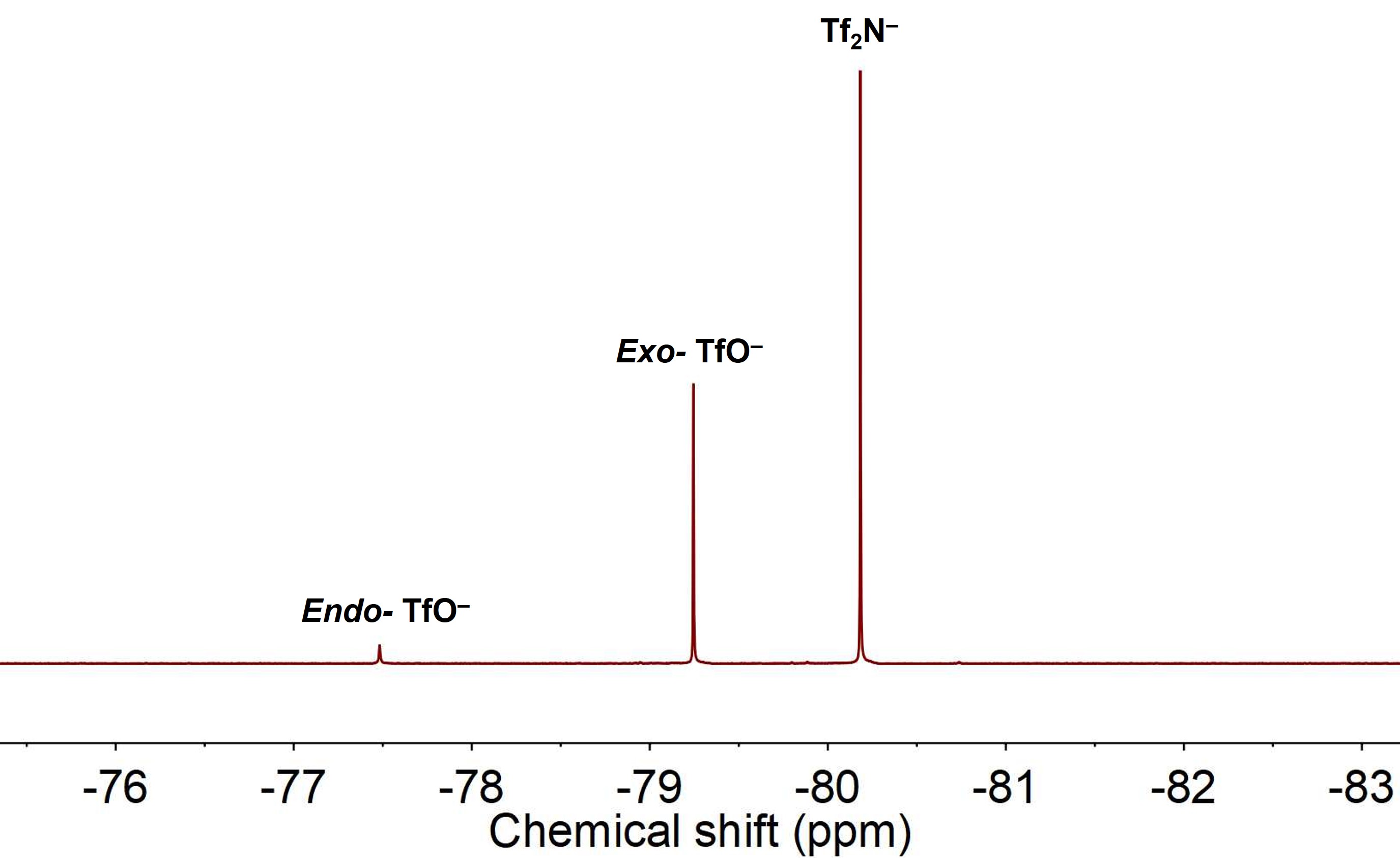


**Figure S52** ^19^F NMR spectra (376 MHz, 298 K, CD_3_CN) of 0.4 mM solution of **6** following the addition of 8 equiv. of KOTf and heating at 50 °C for 1 hour. Observation of the *endo-*binding signal of TfO^–^ indicates formation of TfO^–^⊂**7**.

### 7.2 Addition of triflate to **4**.

The exterior crosslinkers prevent ligands reorientation in **4**.


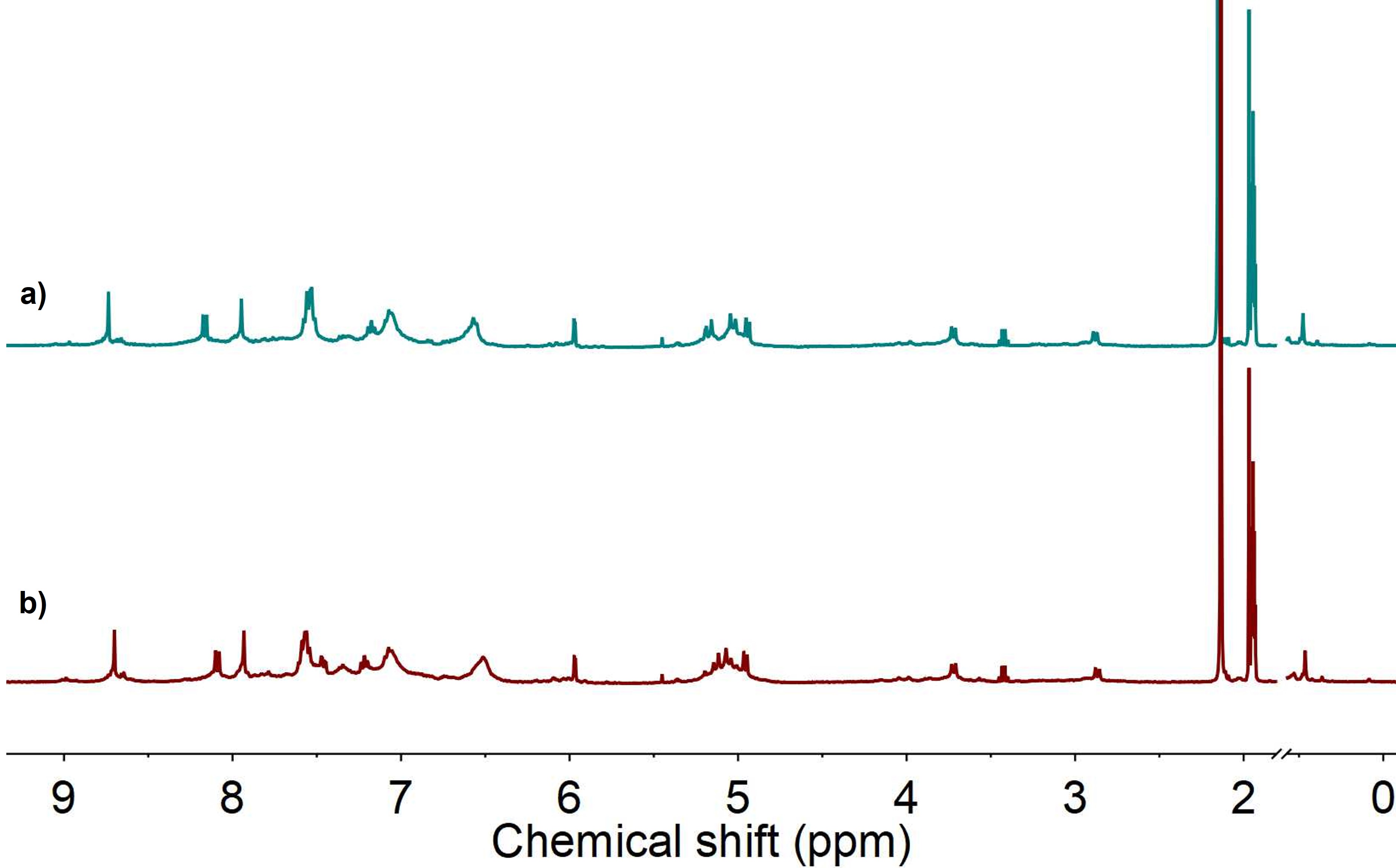


**Figure S53**. ^1^H NMR spectra (400 MHz, CD_3_CN) comparison of **a)** 0.4 mM **4**; **b)** 0.4 mM solution of **4** following the addition of 16 equiv. of KOTf and heating at 50 °C for 12 hours. No significant shift in the methyl proton signals was observed, indicating the absence of interior binding of TfO^–^.


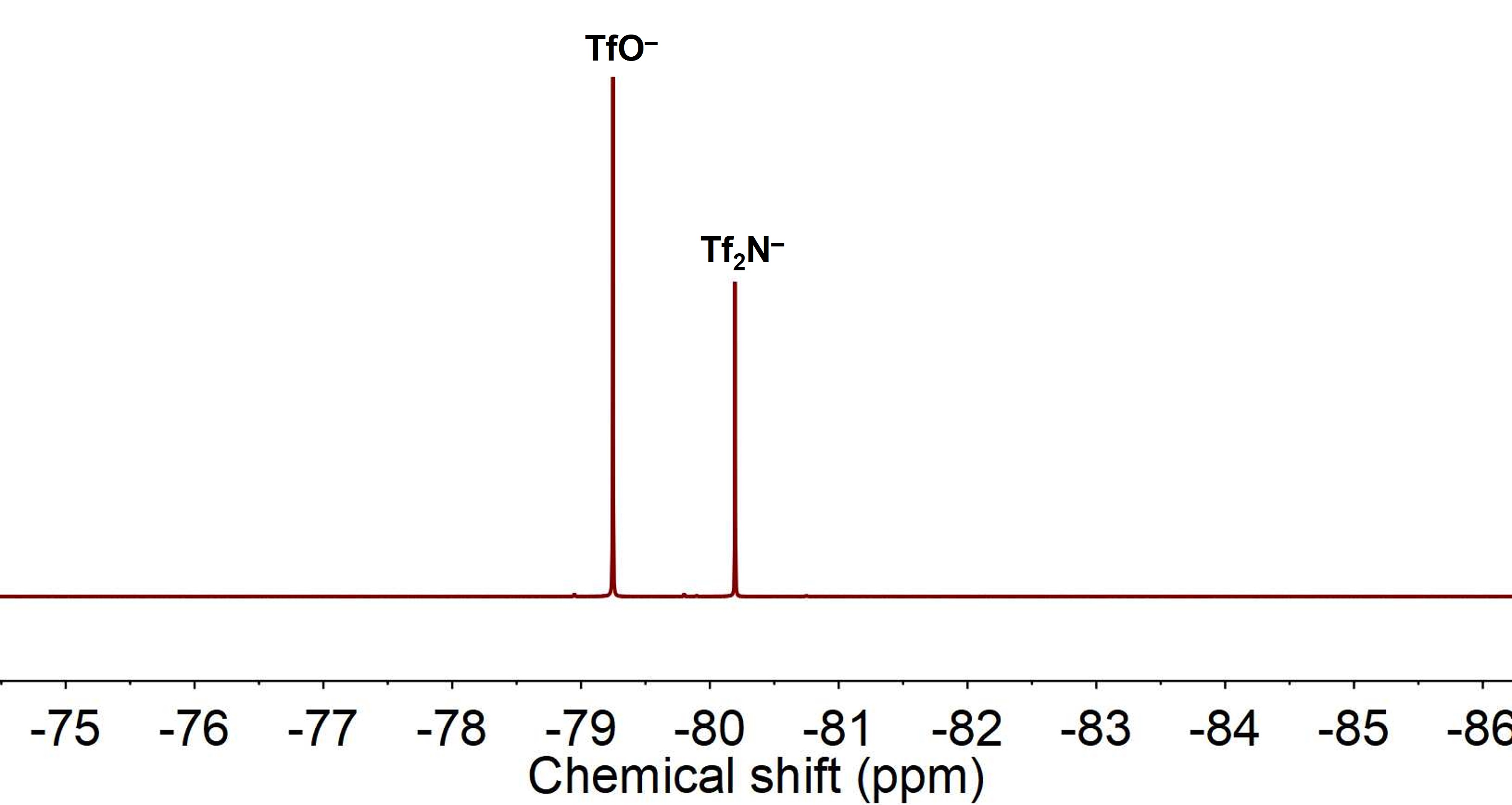


**Figure S54.** ^19^F NMR spectrum (376 MHz, 298 K, CD_3_CN) of0.4 mM solution of **4** following the addition of 16 equiv. of KOTf and heating at 50 °C for 12 hours. No interior binding signals of TfO^–^ were observed.

# **8.** Self-assembly between Subcomponents A, B, C and *p*-toluidine


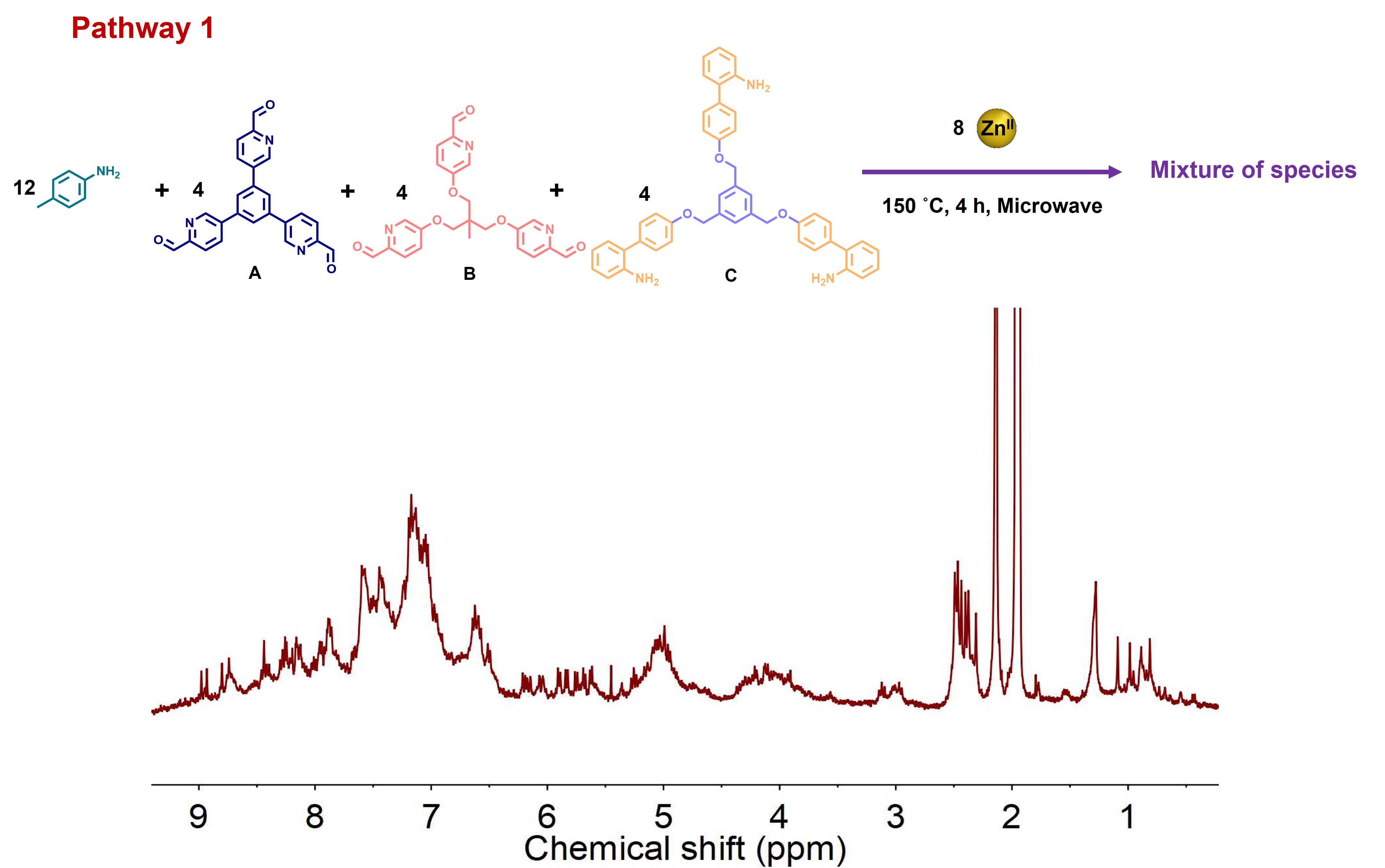


**Figure S55**. ^1^H NMR spectrum (400 MHz, CD_3_CN) of the product mixture from Figure 4 Pathway 1.


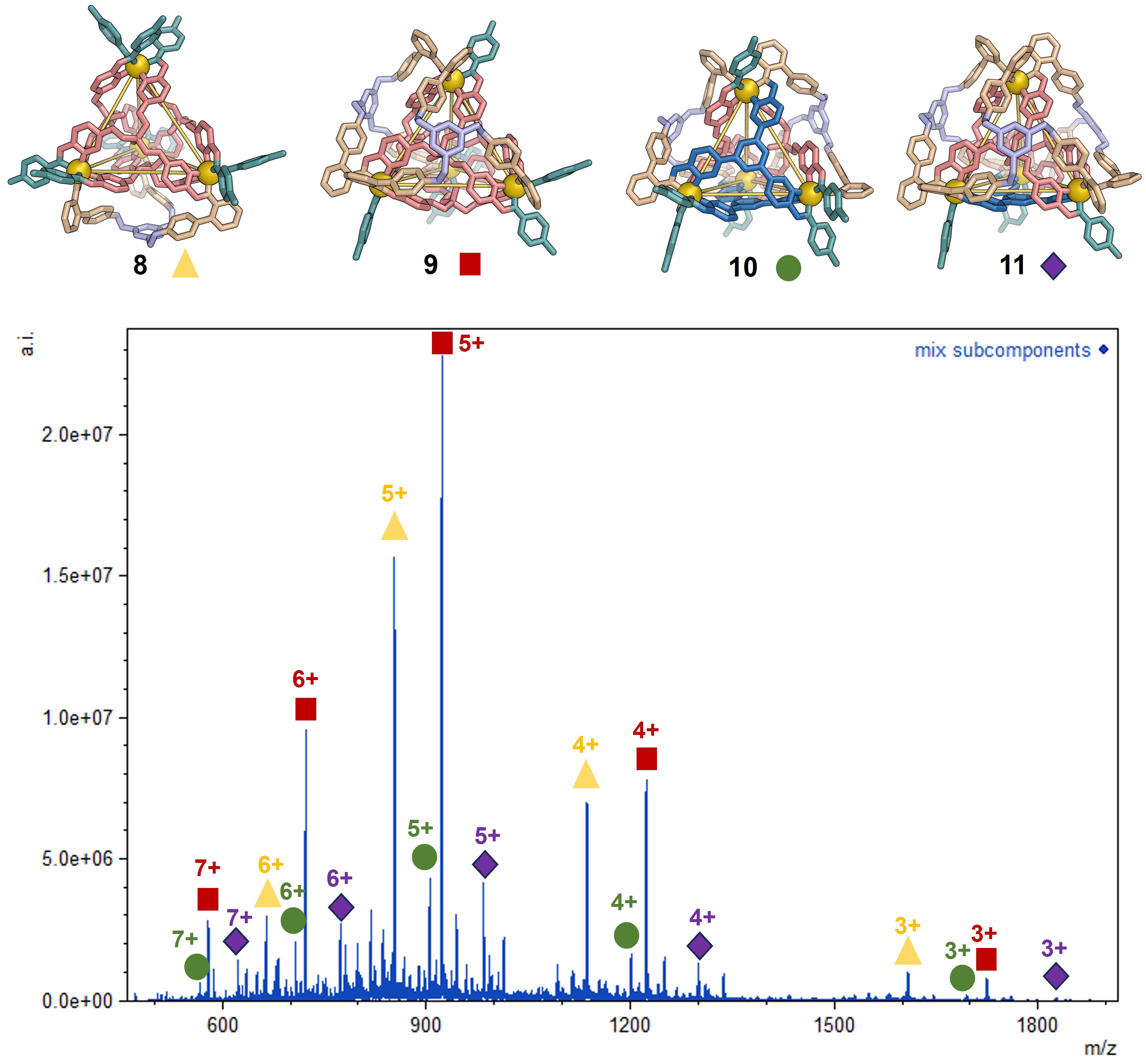


**Figure S56**. ESI-MS spectrum of the product mixture from Figure 4 Pathway 1. Signals belonging to possible structures **8**, **9**, **10**, **11** were observed. The formula of these structures (remove 8 Tf_2_N^–^) are listed as follows: **8** Zn_4_C_248_H_420_N_24_O_21_; **9** Zn_4_C_224_H_384_N_24_O_18_; **10** Zn_4_C_226_H_384_N_24_O_12_; **11** Zn_4_C_249_H_420_N_24_O_18_.


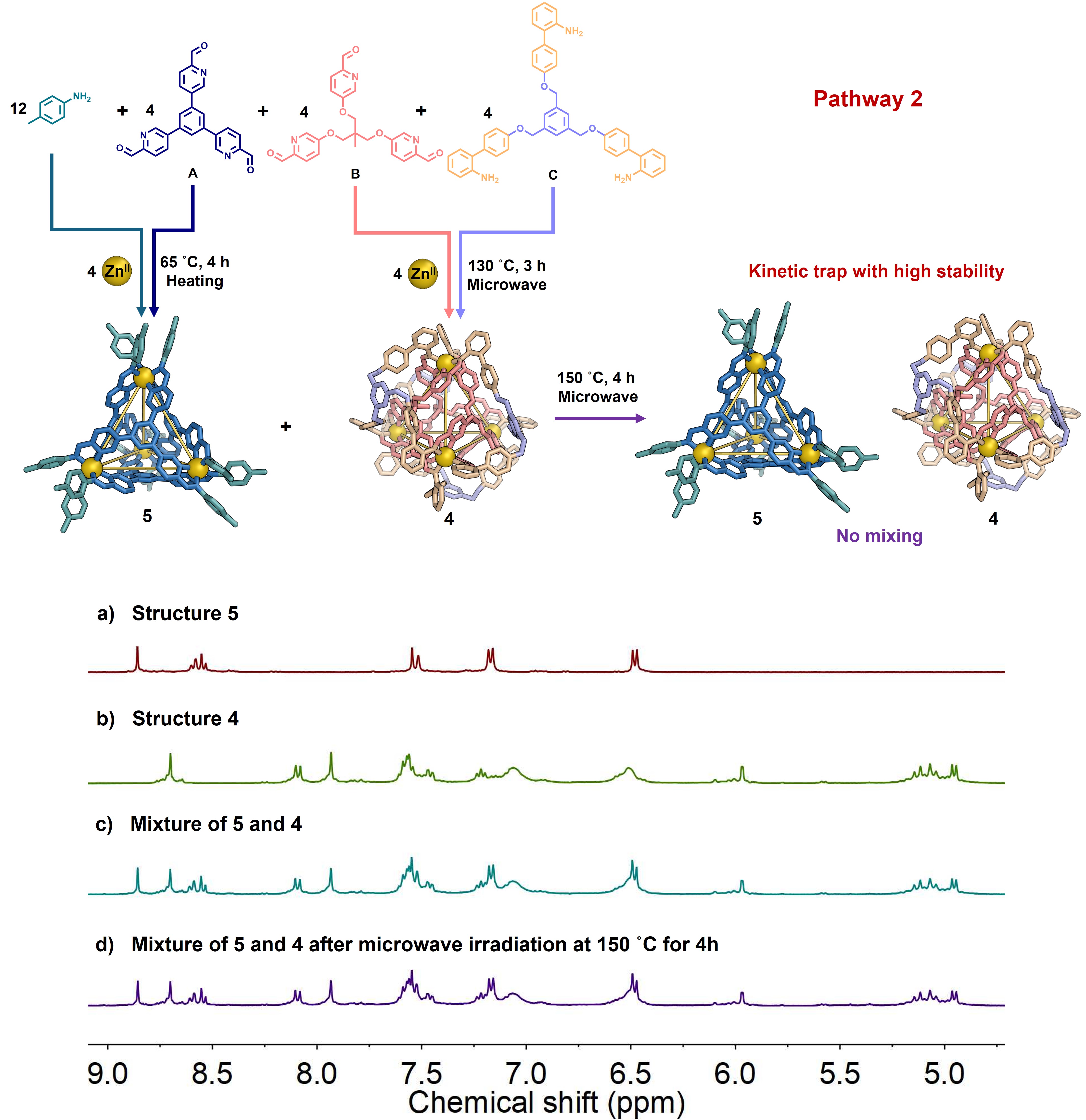


**Figure S57**. Aromatic region the ^1^H NMR spectra (400 MHz, CD_3_CN) of Figure 4 Pathway 2. **a)** Pure cage **5**; **b)** Pure cage **4**; **c)** Mixture of 0.5 mM **5** and 0.5 mM **4**; **d)** The mixture in **c)** was heated under microwave irradiation at 150 °C for 4 hours. No product mixing was observed.


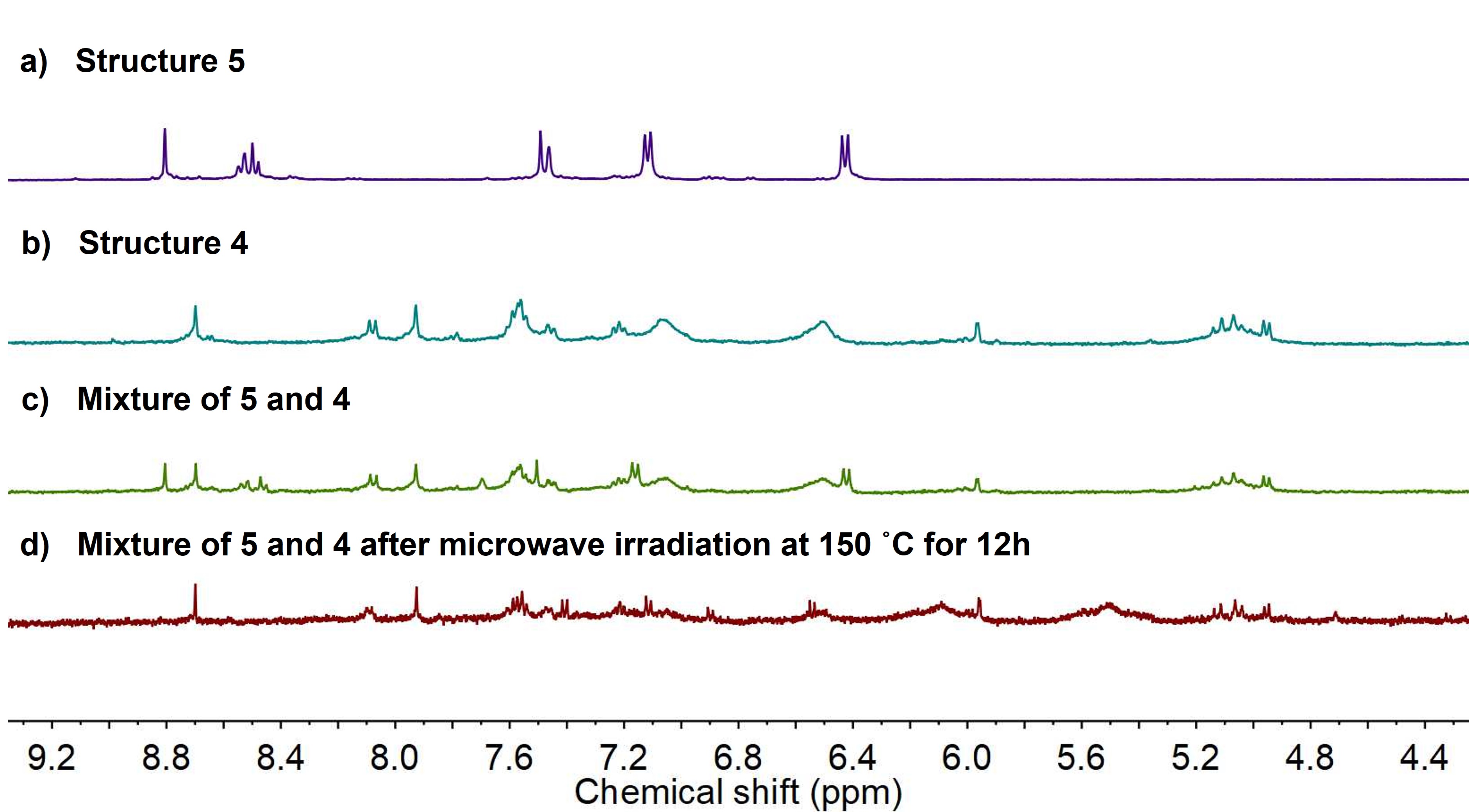


**Figure S58**. Aromatic region of the ^1^H NMR spectra (400 MHz, CD_3_CN) of **a)** Pure cage **5**; **b)** Pure cage **4**; **c)** Mixture of 0.5 mM **5** and 0.5 mM **4**; **d)** The mixture in **c)** after heating under microwave irradiation to 150 °C for 12 hours, where **5** had decomposed.


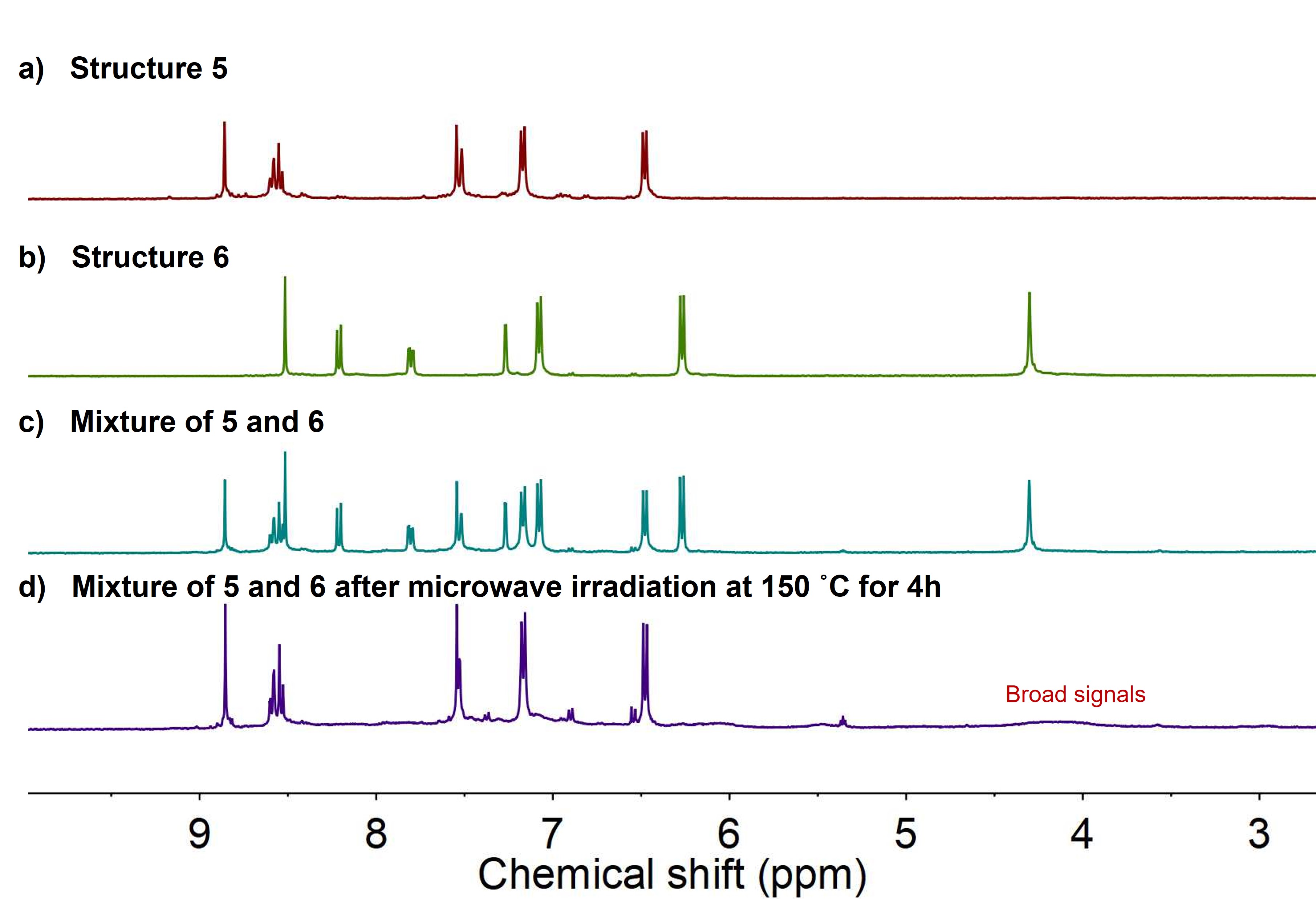


**Figure S59**. Partial ^1^H NMR spectra (400 MHz, CD_3_CN) of **a)** Pure cage **5**; **b)** Pure cage **6**; **c)** Mixture of 0.5 mM **5** and 0.5 mM **6**; **d)** The mixture in **c)** was heated under microwave irradiation at 150 °C for 4 hours. The disappearance of **6** and the appearance of broad signals indicate the decomposition of **6** during the reaction.


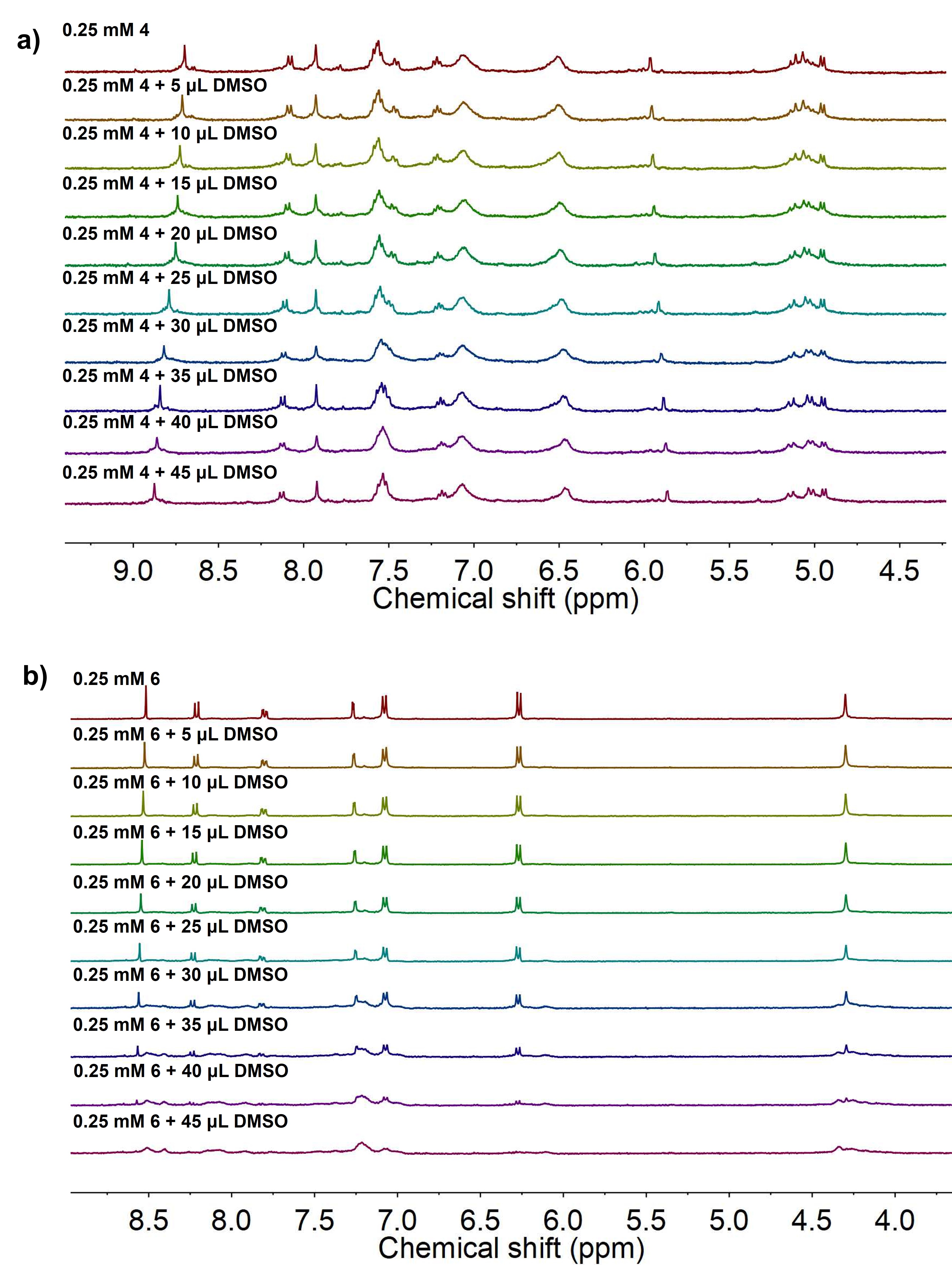


**Figure S60**. Partial ^1^H NMR spectra of 0.25 mM a) **4** and b) **6** following the addition of progressively larger amounts of *d_6_*-DMSO (400 MHz, CD_3_CN, 298 K). the addition of 45 μL of deuterated DMSO to **6** led to decomposition, whereas its knotted counterpart **4** remained intact under the same conditions.

# **9. X-ray Crystallography**

Data were collected at Beamline I19 of Diamond Light Source employing silicon double crystal monochromated synchrotron radiation (0.6889 Å) with ω and *ψ* scans at 100(2) K.^10^ Data integration and reduction were undertaken with Xia2.^11–13^ Subsequent computations were carried out using the WinGX-32 graphical user interface.^14^ Absorption corrections using spherical harmonics were applied to the data using DIALS.^12,15^ The structures were solved by direct methods using SHELXT^16^ then refined and extended with SHELXL.^17^In general, non-hydrogen atoms with occupancies greater than 0.5 were refined anisotropically. Carbon-bound hydrogen atoms were included in idealized positions and refined using a riding model. Disorder was modelled using standard crystallographic methods including constraints, restraints and rigid bodies where necessary. Crystallographic data along with specific details pertaining to the refinement follow. Crystallographic data have been deposited with the CCDC (2477423-2477424).

Structure **1**

Formula C223H144F48N26O41S16Zn4, *M* 5530.07, Trigonal, space group R -3 c :H (#167), *a* 23.46240(10), *b* 23.46240(10), *c* 167.2974(9) Å, *γ *120º, *V* 79756.2(8) Å3, *D*c 1.382 g cm-3, *Z* 12, crystal size 0.15 by 0.10 by 0.10 mm, color yellow, habit prism, temperature 100(2) Kelvin, *λ*(Synchrotron) 0.6889 Å, *μ*(Synchrotron) 0.535 mm-1, *T*(Analytical)min,max 0.9265540506525894, 1.0, 2*θ*max 40.29, *hkl* range -23 23, -23 23, -164 164, *N* 89127, *N*ind 9245(*R*merge 0.0323), *N*obs 5076(I > 2σ(I)), *N*var 881, residuals* *R*1(*F*) 0.1326, *wR*2(*F*2) 0.4706, GoF(all) 1.143, Δ*ρ*min,max -0.504, 1.517 e- Å-3.

**R*1 = ||*F*o| - |*F*c||/|*F*o| for *F*o > 2σ(*F*o); *wR*2 = (w(*F*o2 - *F*c2)2/(w*F*c2)2)1/2 all reflections, w=1/[2(*F*o2)+(0.3427P)2] where P=(*F*o2+2*F*c2)/3

*Specific refinement details:*

The crystals with composition [Zn_4_L]·8NTf_2_[+ solvent] were grown by diffusion of diethyl ether into an acetonitrile solution of the complex. The crystals employed immediately lost solvent after removal from the mother liquor and rapid handling prior to flash cooling in liquid nitrogen was required to collect data. Despite these measures and the use of synchrotron radiation few reflections at greater than 1.0 Å resolution were observed and the data were trimmed accordingly. The diffraction was broad, and the quality of the integration is less than ideal hence the values of the R1, wR and wR2 factors are larger than for typical small molecule structures. Nevertheless, the quality of the data is far more than sufficient to establish the connectivity of the structure. The asymmetric unit was found to contain one third of a Zn_4_L assembly and associated counterions.

Due to the limited resolution of the data and high degree of thermal motion throughout the structure, bond length and angle restraints were required in order to obtain a reasonable model for some of the organic parts of the structure. The GRADE program^18^ was thus employed, using the GRADE Web Server,^19^ to generate a full set of bond distance and angle restraints (DFIX, DANG, FLAT) for the organic ligands as required. Thermal parameter restraints (SIMU, RIGU) were also applied to all atoms except for zinc to facilitate anisotropic refinement. Even with these restraints the thermal parameters of both the main residue and the anions remain higher than ideal.

One of the triflimide anions was modelled as disordered around a *C*_3_ axis. Bond length restraints were applied to all anions and the disordered triflimide was mostly refined isotropically. The occupancy of the other located triflimide anion was allowed to freely refine. There are some remaining electron density peaks close to the anions but no further discrete positions for their disorder could be resolved. The remaining anions (4.5 per Zn_4_L assembly, included as triflimide in the formula) and solvent within the lattice were significantly disordered and despite numerous attempts at modeling, including with rigid bodies no satisfactory model for the electron-density associated with them could be found. Consequently, the SQUEEZE^20^ function of PLATON^21^ was employed to remove the contribution of the electron density associated with these remaining anions and further highly disordered solvent, which gave a potential solvent accessible void of 27819 Å^3^ per unit cell (a total of approximately 9973 electrons). The diffuse solvent molecules could not be assigned to acetonitrile or diethyl ether and were also not included in the formula. Consequently, the molecular weight and density given above are underestimated.

CheckCIF gives two A and one B level alert. These alerts all result from the limited resolution of the data (low sine(theta_max)/wavelength, low bond precision) and poor diffraction properties of the crystals (high wR2 value, low bond precision) as described above.


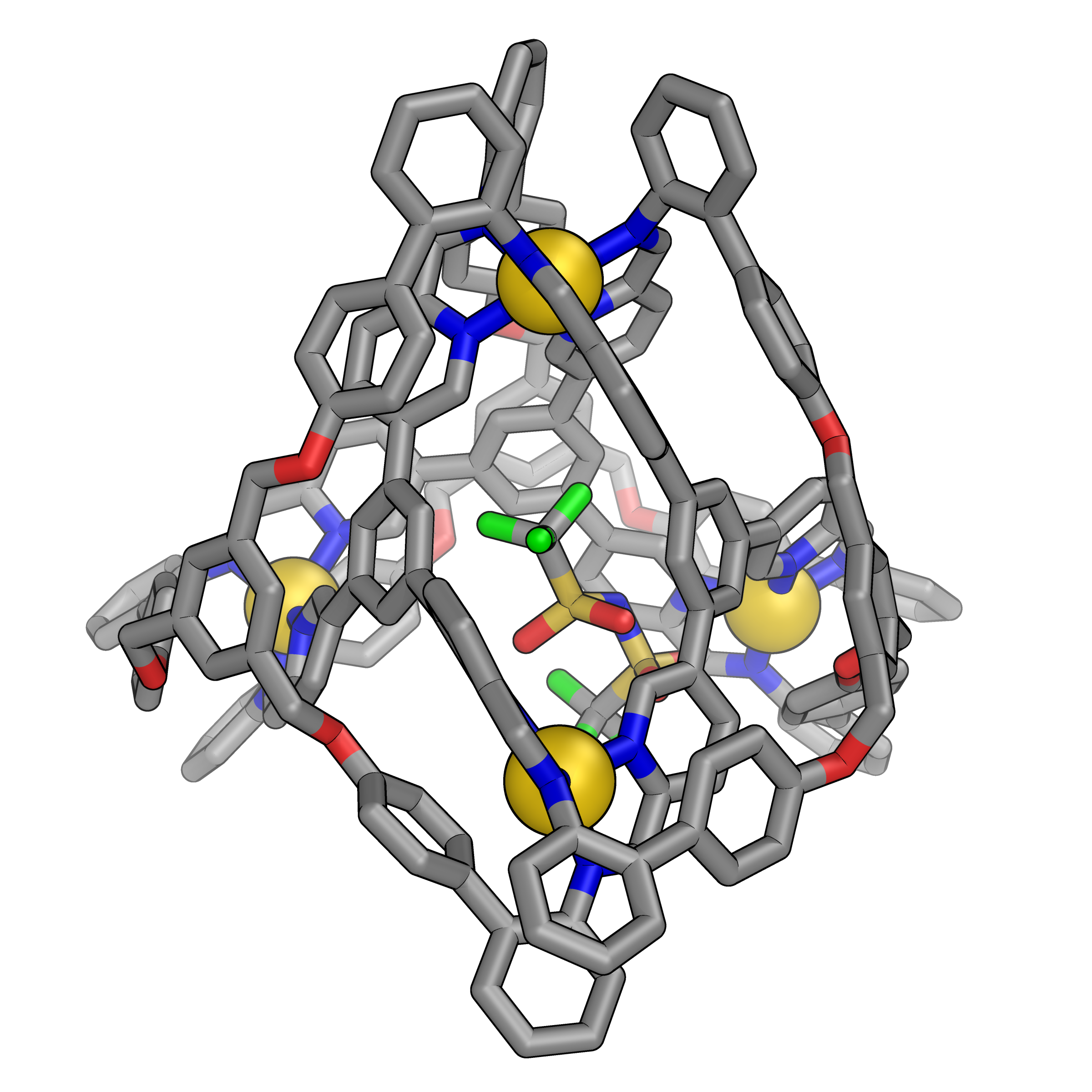


**Figure S61**. Crystal structure of Tf_2_N^–^⊂**1**. (Zn: yellow, N: blue, O: red, C: gray, F: green). Disorder, hydrogen atoms, non-encapsulated counterions and solvents are omitted for clarity.


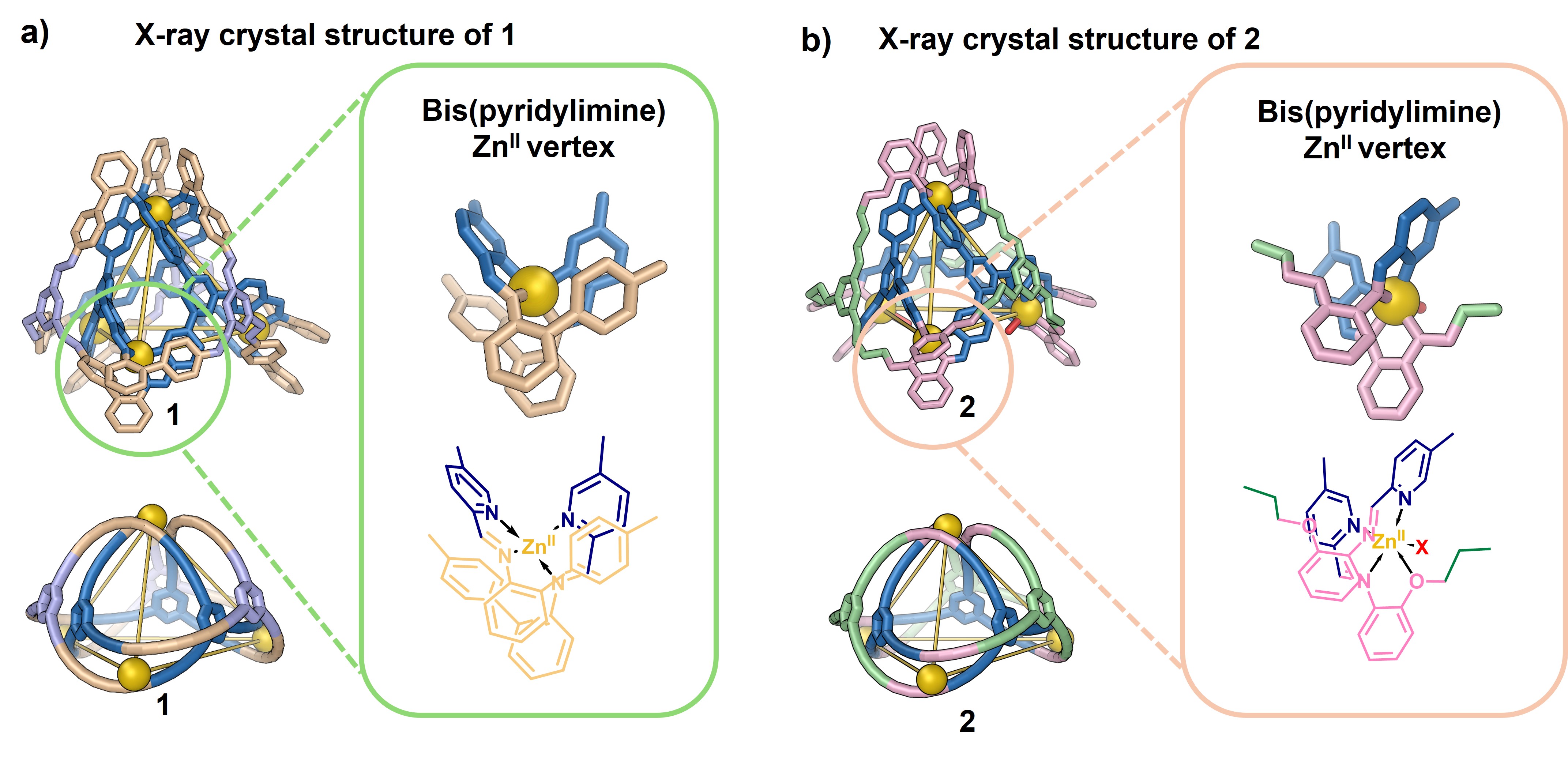


**Figure S62**. Comparison of the crystal structures of perplexanes **1** and **2**^3^. a) **1** features three distorted tetrahedral bis(pyridylimine) vertices, whereas b) **2** comprises three octahedral bis(pyridylimine) vertices accompanied by an additional counterion (indicated as a red ‘X’ in the expanded view).


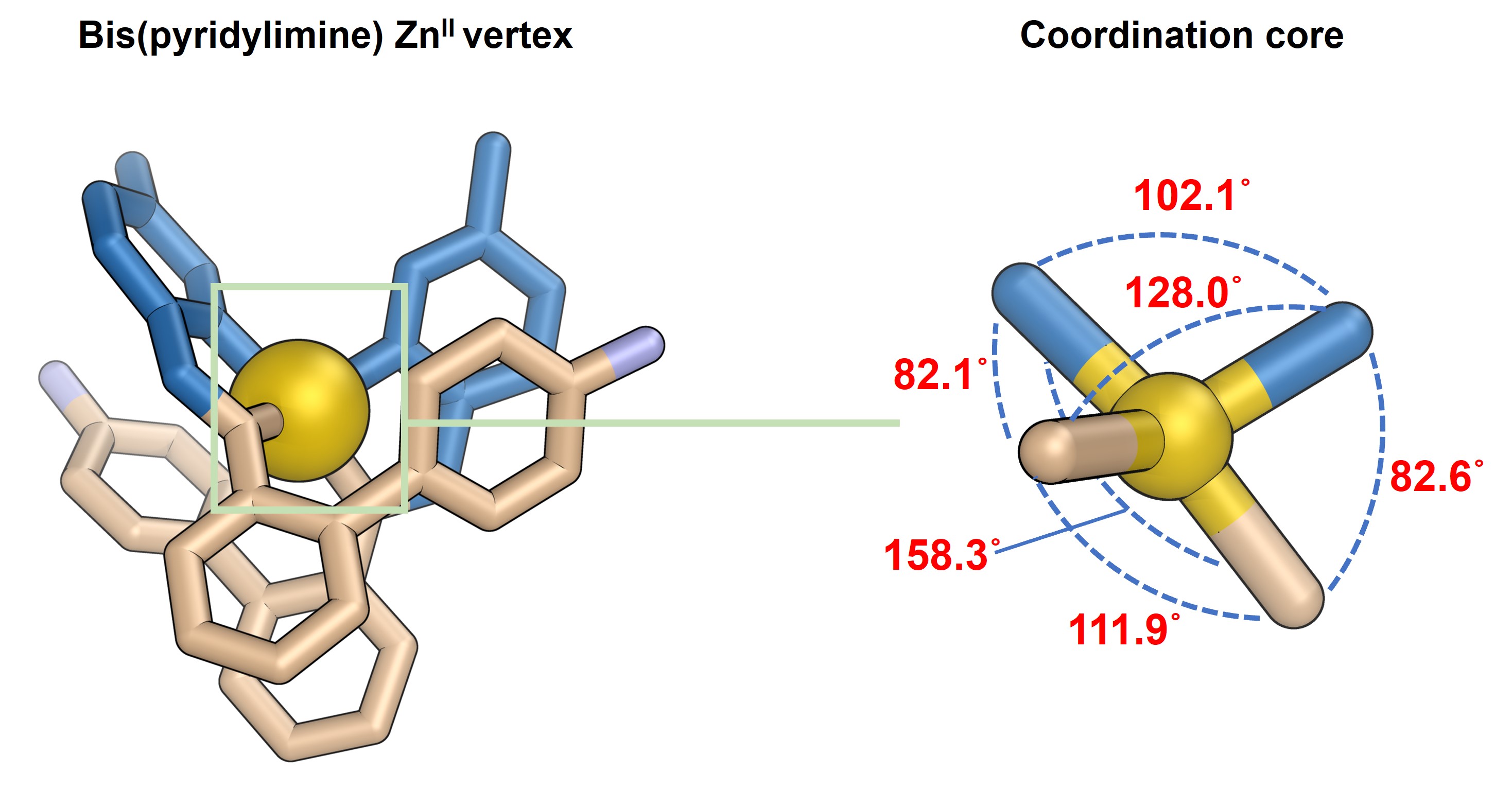


**Figure S63.** Measurements of six N- Zn^II^-N angles of bis(pyridylimine) Zn^II^ vertex.

The bond angle variance^22^ (σ^2^) was calculated as follows:

$$\sigma^{2}=\frac{1}{5}\sum_{i=1}^{6} {(\theta_{i}-109.47^{\circ})}^{2}\approx852{deg}^{2}$$

The calculated σ^2^ value of 852 deg^2^ reflects a highly distorted tetrahedral coordination environment.


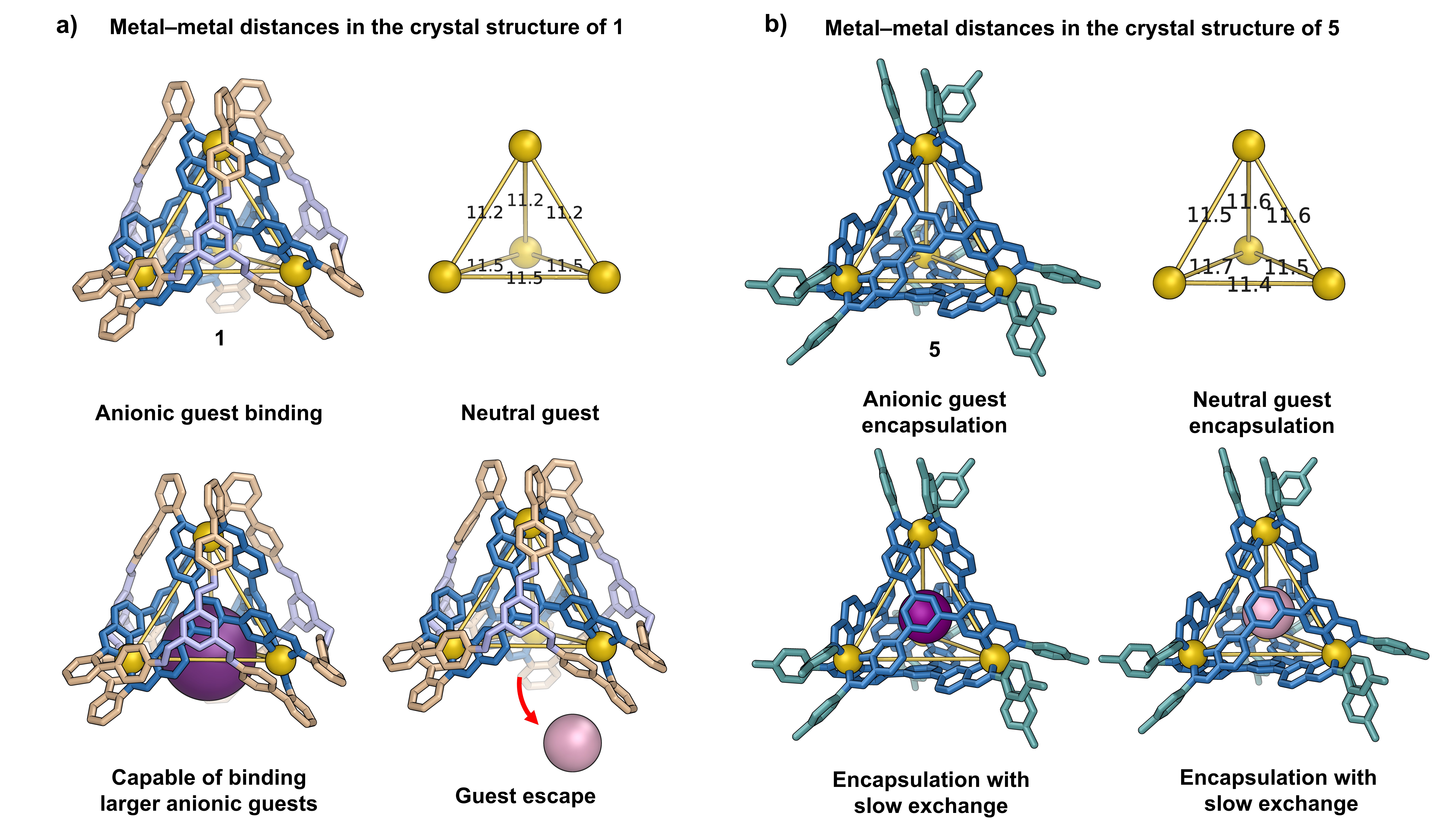


**Figure S64.** Size comparison and schematic representation of guest binding in a) **1** and b) **5**. Metal–metal distances are given in Å.

Structure **6: [Zn_4_L_4_]·8NTf_2_·5.75MeCN** [+ solvent]

Formula C203.50H185.25F48N37.75O44S16Zn4, *M* 5550.07, Monoclinic, space group P 21/c (#14), *a* 26.3514(2), *b* 40.1967(2), *c* 26.01710(10) Å, *β *116.2740(10), *V* 24711.2(3) Å3, *D*c 1.492 g cm-3, *Z* 4, crystal size 0.080 by 0.080 by 0.050 mm, color colorless, habit block, temperature 100(2) Kelvin, *λ*(Synchrotron) 0.6889 Å, *μ*(Synchrotron) 0.578 mm-1, *T*(Analytical)min,max 0.9791792438258899, 1.0, 2*θ*max 45.00, *hkl* range -29 28, -44 44, -28 27, *N* 158676, *N*ind 35063(*R*merge 0.0606), *N*obs 20088(I > 2σ(I)), *N*var 3752, residuals* *R*1(*F*) 0.0952, *wR*2(*F*2) 0.2844, GoF(all) 0.983, Δ*ρ*min,max -0.685, 1.226 e- Å-3.

**R*1 = ||*F*o| - |*F*c||/|*F*o| for *F*o > 2σ(*F*o); *wR*2 = (w(*F*o2 - *F*c2)2/(w*F*c2)2)1/2 all reflections, w=1/[2(*F*o2)+(0.1969P)2] where P=(*F*o2+2*F*c2)/3

*Specific refinement details:*

The crystals of with composition [Zn_4_L_4_]·8NTf_2_·5.75MeCN[+ solvent] were grown by diffusion of diethyl ether into an acetonitrile solution of the complex. Rapid handling prior to flash cooling in liquid nitrogen and the use of synchrotron radiation enabled data to be collected to ca. 0.9 Å resolution. The asymmetric unit was found to contain one complete Zn_4_L_4_ assembly and associated counterions.

Due to the limited resolution, thermal parameter restraints (SIMU, RIGU) were applied to all atoms except for zinc. Two of the outer toluidine groups were modelled as disordered over two positions and the aromatic rings of some toluidine groups were modelled as rigid groups (AFIX 66).

The anions within the structure show evidence of substantial disorder. Six of the triflimide anions were modelled as disordered over two or three locations. Some lower occupancy disordered atoms were modelled with isotropic thermal parameters and bond length and thermal parameter restraints were applied to facilitate realistic modeling of all disordered triflimideanions. There is evidence of further disorder for some of the triflimide anions which could not be resolved due to the limited resolution and complexity of the disorder. Bond length restraints were also applied to some solvent molecules and low occupancy solvents were refined isotropically. The hydrogen atoms of one low occupancy acetonitrile molecule could not be located in the electron density map and were therefore not included in the model.

CheckCIF gives one B alert resulting from the limited resolution of the data.

# **10.** References

[1] M. P. Sibi, G. Petrovic, Enantioselective Radical Reactions: The use of metal triflimides as lewis acids. *Tetrahedron Asymmetry* **2003**, *14* (19), 2879–2882.

[2] J. Mosquera, T. K. Ronson, J. R. Nitschke, Subcomponent flexibility enables conversion between *D*_4_-symmetric Cd^II^_8_L_8_ and *T*-symmetric Cd^II^_4_L_4_ assemblies. *J. Am. Chem. Soc.* **2016**, *138* (6), 1812–1815.

[3] Y. Yang, T. K. Ronson, P. C. P. Teeuwen, S. Zucchelli, A. W. Heard, P. Posocco, D. J. Wales, J. R. Nitschke, Synthesis of covalently linked knotted cage frameworks. *Nat. Synth.* **2025**, DOI: 10.1038/s44160-025-00822-7.

[4] OPTIM: A program for geometry optimisation and pathway calculations. <http://www-wales.ch.cam.ac.uk/software.html>.

[5] P. A. Wesołowski, D. J. Wales, P. Pracht, Multilevel framework for analysis of protein folding involving disulfide bond formation. *J. Phys. Chem. B* **2024**, *128*, 3145–3156.

[6] C. Bannwarth, S. Ehlert, S. Grimme, GFN2-XTB—An accurate and broadly parametrized self-consistent tight-binding quantum chemical method with multipole electrostatics and density-dependent dispersion contributions. *J Chem Theory Comput* **2019**, *15* (3), 1652–1671.

[7] M. Bursch, H. Neugebauer, S. Grimme, Structure optimisation of large transition‐metal complexes with extended tight‐binding methods. *Angew. Chem. Int. Ed.* **2019**, *58*, 11078–11087.

[8] N. Marchand, P. Lienard, H.-U. Siehl, H. Izato, *Applications of Molecular Simulation Software SCIGRESS in Industry and University*; 2014; Vol. 50.

[9] A. M. Castilla, T. K. Ronson, J. R. Nitschke, Sequence-dependent guest release triggered by orthogonal chemical signals. *J. Am. Chem. Soc.* **2016**, *138* (7), 2342–2351.

[10] D. R. Allan, H. Nowell, S. A. Barnett, M. R. Warren, A. Wilcox, J. Christensen, L. K. Saunders, A. Peach, M. T. Hooper, L. Zaja, S. Patel, L. Cahill, R. Marshall, S. Trimnell, A. J. Foster, T. Bates, S. Lay, M. A. Williams, P. V. Hathaway, G. Winter, M. Gerstel, R. W. Wooley, A novel dual air-bearing fixed-χ diffractometer for small-molecule single-crystal X-ray diffraction on beamline I19 at diamond light source. *Crystals* **2017**, *7* (11), 336.

[11] P. Evans, Scaling and assessment of data quality. In *Acta Crystallographica Section D: Biological Crystallography*; International Union of Crystallography, **2006** (62), 72–82.

[12] G. Winter, D. G. Waterman, J. M. Parkhurst, A. S. Brewster, R. J. Gildea, M. Gerstel, L. Fuentes-Montero, M. Vollmar, T. Michels-Clark, I. D. Young, N. K. Sauter, G. Evans, DIALS: Implementation and evaluation of a new integration package. *Acta Crystallogr. D Struct. Biol.* **2018**, *74* (2), 85–97.

[13] G. Winter, Xia2: An expert system for macromolecular crystallography data reduction. *J. Appl. Crystallogr.* **2010**, *43* (1), 186–190.

[14] L. J. Farrugia, WinGX and ORTEP for Windows: An update. *J Appl Crystallogr* **2012**, *45* (4), 849–854.

[15] J. Beilsten-Edmands, G. Winter, R. Gildea, J. Parkhurst, D. Waterman, G. Evans, Scaling diffraction data in the *DIALS* software package: Algorithms and new approaches for multi-crystal scaling. *Acta Crystallogr D Struct Biol* **2020**, *76* (4), 385–399.

[16] G. M. Sheldrick, SHELXT - Integrated Space-Group and Crystal-Structure Determination. *Acta Crystallogr. A* **2015**, *71* (1), 3–8.

[17] G. M. Sheldrick, Crystal structure refinement with *SHELXL*. *Acta Crystallogr. C Struct. Chem.* **2015**, *71* (1), 3–8.

[18] G. Bricogne, E. Blanc, M. Brandle, C. Flensburg, P. Keller, W. Paciorek, P. Roversi, A. Sharff, O. S. Smart, C. Vonrhein, T. O. Womack, *BUSTER. 2.11.2 Ed.; Global Phasing Ltd.: Cambridge, United Kingdom, 2011.*

[19] O. S. Smart, T. O. Womack, Grade Web Server. Global Phasing Ltd.: 2014.

[20] P. Van Der Sluis, A. L. Spek, BYPASS: An effective method for the refinement of crystal structures containing disordered solvent regions. *Acta Crystallogr. A* **1990**, *46* (3), 194–201.

[21] A. L. Spek, *PLATON: A Multipurpose Crystallographic Tool*; Utrecht University: Utrecht, The Netherlands, 2008.

[22] Z. Qi, K. Zhang, X. Zhao, N. Zhang, S.-L. Li, X.-M. Zhang, Promoting structural distortion to enhance the crystal field strength of Mn(II) in tetrahedral bromide for near-unity yellow emission. *Chem. Commun.* **2024**, *60*, 12880.
